# Robust and resource-optimal dynamic pattern formation of Min proteins in vivo

**DOI:** 10.1038/s41567-025-02878-w

**Published:** 2025-05-05

**Authors:** Ziyuan Ren, Henrik Weyer, Michael Sandler, Laeschkir Würthner, Haochen Fu, Chanin B. Tangtartharakul, Dongyang Li, Cindy Sou, Daniel Villarreal, Judy E. Kim, Erwin Frey, Suckjoon Jun

**Affiliations:** 1 Department of Physics, University of California San Diego, La Jolla, CA USA; 2 Arnold Sommerfeld Center for Theoretical Physics and Center for NanoScience, Ludwig-Maximilians-Universität München, Munich, Germany; 3 Department of Chemistry and Biochemistry, University of California San Diego, La Jolla, CA USA; 4 Max Planck School Matter to Life, Munich, Germany; 5 Present Address: Developmental Biology Unit, European Molecular Biology Laboratory, Heidelberg, Germany

**Keywords:** Biophysics, Microbiology

## Abstract

The Min protein system prevents abnormal cell division in bacteria by forming oscillatory patterns between cell poles. However, predicting the protein concentrations at which oscillations start and whether cells can maintain them under physiological perturbations remains challenging. Here we show that dynamic pattern formation is robust across a wide range of Min protein levels and variations in the growth physiology using genetically engineered *Escherichia coli* strains. We modulate the expression of *minCD* and *minE* under fast- and slow-growth conditions and build a MinD versus MinE phase diagram that reveals dynamic patterns, including travelling and standing waves. We found that the natural expression level of Min proteins is resource-optimal and robust to changes in protein concentration. In addition, we observed an invariant wavelength of dynamic Min patterns across the phase diagram. We explain the experimental findings quantitatively with biophysical theory based on reaction–diffusion models that consider the switching of MinE between its latent and active states, indicating its essential role as a robustness module for Min oscillation in vivo. Our results underline the potential of integrating quantitative cell physiology and biophysical modelling to understand the fundamental mechanisms controlling cell division machinery, and they offer insights applicable to other biological processes.

## Abstract (web summary)

Oscillatory Min protein patterns prevent abnormal bacterial cell division. Now it is shown that Min pattern formation is resource efficient and involves wavelength-invariant oscillations that are robust to physiological changes.

## Main

**Fig. 1:**
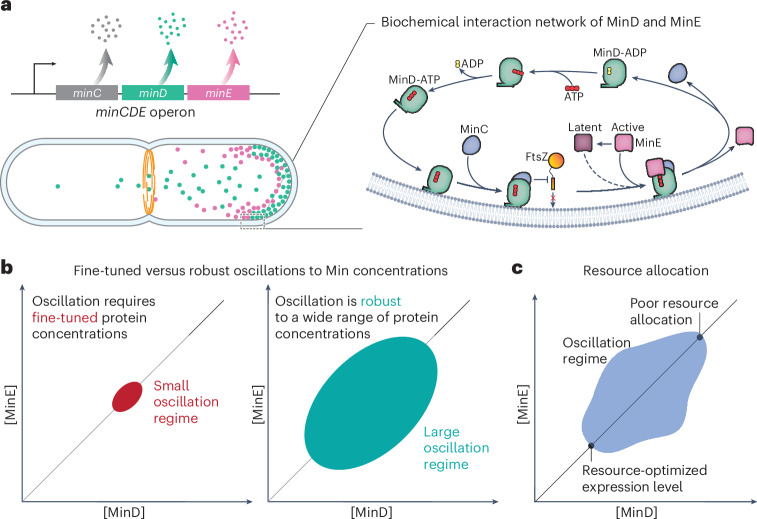
The Min system in the context of quantitative cell physiology. **a**, The *minCDE* genes are organized as an operon (top left). In the bacterial cell, MinD (green) and MinE (magenta) self-organize to perform pole-to-pole oscillations, thereby guiding the bacterial division machinery (Z-ring, shown in orange) to the midplane (bottom left). The active form MinD-ATP binds to the membrane (grey lipid bilayer) and recruits MinC, which inhibits the polymerization of the division protein FtsZ (right). MinE can switch from an active to a latent form^10,26^, and the membrane-bound MinD-ATP mainly recruits the active MinE. MinE stimulates ATP hydrolysis by MinD (MinD-ATP → MinD-ADP), and MinD-ADP is released to the cytoplasm. The pole-to-pole oscillations resulting from this biochemical reaction–diffusion system ensure that the concentration of MinD-ATP is highest at the cell poles, thus inhibiting cell division therein. **b**, So far, it is unknown at what Min protein concentrations pole-to-pole oscillations start, and whether the oscillations are sensitive (left) or robust (right) to physiological changes in or perturbations to the protein concentrations. **c**, A related question is whether the production of Min proteins is resource-optimal in wild-type bacteria.

Pattern formation, a fundamental biological process, drives organized development and functional differentiation. In *Escherichia coli*, the Min protein system exemplifies this through pole-to-pole oscillations that ensure symmetrical cell division (Fig. 1a). Despite extensive studies in genetics^1–5^, structural biology^6–11^, biochemistry^12–19^ and biophysics^19–25^, key questions concerning cell physiology remain unresolved.

We address how physiological perturbations, such as changes in the growth rate and of gene expression levels, affect Min oscillations. Importantly, pattern formation is based on feedback in the protein interactions, which is generally concentration-dependent^24,26,27^. We, therefore, answer two critical questions. Robustness—How robust is the wild-type dynamic pattern formation to changes in Min protein concentrations (Fig. 1b)? Resource-optimality: What are the minimum Min protein concentrations required for oscillations and symmetric cell division, and how close are wild-type concentrations to these concentration thresholds (Fig. 1c)?

To investigate the robustness of Min pattern formation in vivo, we systematically perturbed the MinD and MinE concentrations and studied their impacts on pattern formation, given that their interactions alone drive oscillations. To achieve this, we engineered *E. coli* strains for independent control of *minCD* and *minE* expression levels, enabling us to construct the in vivo MinD versus MinE phase diagram of dynamic pattern formation. Importantly, only the protein numbers were modulated and not the nature of Min protein interactions (namely, the protein ‘interaction network’; Fig. 1a). Our experiments spanned from slow (doubling time *τ* = 55 min) to fast (*τ* = 25 min) growth conditions, broadening the scope beyond previous studies that primarily examined rapidly growing cells^28–32^.

We complemented our experimental study with biophysical theory by applying reaction–diffusion models that are grounded in the known biochemical reactions of the Min system^26^^,^^21^^,^^24^. Our integrative approach—merging theory with extensive in vivo data—provided a comprehensive understanding of Min oscillations in vivo. Here, we present a quantitative model that predicts the pattern types, oscillation periods and pattern wavelengths within living cells.

Our results highlight the role of the rapid conformational switch of MinE^10,26^, which ensures the robustness of the system against fluctuations in expression levels and growth conditions. The resulting interaction network quantitatively explains the notably constant wavelength of Min pattern formation observed across the phase diagram. Although co-expression of MinCDE protein productions is not essential for wild-type pattern formation, our findings indicate that endogenous expression levels closely approach the onset concentrations for pole-to-pole oscillations, indicating that *E. coli* optimizes Min protein concentrations in a resource-efficient manner.

## Construction and characterization of the *E. coli* strains for probing the MinD versus MinE concentration phase diagram

**Fig. 2:**
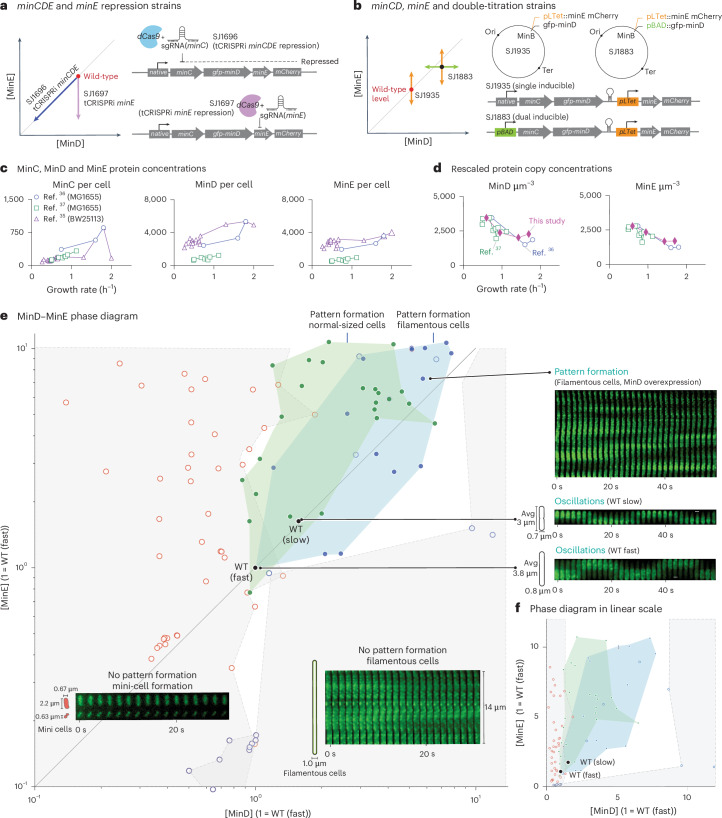
Exploring the [MinD] versus [MinE] phase diagram using titratable strains in vivo. **a**, tCRISPRi strains for gradient repression of the whole *minCDE* operon (SJ1696) or only *minE* (SJ1697). These strains allowed us to decrease the Min concentrations diagonally and vertically in the [MinD] versus [MinE] concentration phase diagram (left). [MinD] and [MinE] refer to MinD and MinE protein concentrations, respectively. **b**, Right, genotypes of the two strains. SJ1935 is a single-inducible strain with the P_LTet_ inducible promoter for gradient expression of *minE*. SJ1883 is a dual-inducible strain with arabinose-inducible promoter P_BAD_ controlling the expression of *minCD* and tetracycline-inducible promoter P_LTet_ controlling the expression of *minE*, independently. Both strains allow the gradient expression of MinE (orange arrows in left panel). The dual-inducible strain additionally allows the independent gradient expression of MinD (green arrows). **c**, MinC, MinD and MinE protein copy numbers per cell versus growth rate measured for *E.coli* strains BW25113 (ref. ^35^) and MG1655 (refs. ^36,37^). **d**, Comparisons of MinD and MinE protein concentrations between the published data and measurements made in this study. To compare the trends, we normalized the fluorescence measurement and the data by Mori et al.^37^ for the slow-growth condition to the Ribo-Seq data^36^. The protein copy number per cell data (**c**) shows the opposite trend compared to the concentration data. **e**, Left, experimentally determined pattern-formation phase diagram in the [MinD] versus [MinE] space. No patterns formed if either both the MinD and MinE concentrations were low or their ratio was severely disproportionate (grey-shaded areas bounded by dashed lines). In the no-pattern regimes, mini-cells formed when [MinE] ≫ [MinD] (red circles; Supplementary Video 4), whereas filamentous cells formed when [MinE] ≪ [MinD] (blue circles, Supplementary Video 2). In the pattern-forming region, we distinguish between normal-sized cells (green-shaded area, green dots) and elongated cells (median length >6 µm; blue-shaded region, blue dots). The concentrations of wild-type cells in fast- and slow-growth conditions are marked. Right, kymographs showing the representative dynamics of GFP-MinD in vivo in different regimes. Throughout the paper, we use the following conversion factor from the fold-changes to protein concentrations: [MinD] = [MinE] = 2,000 µm^−^^3^ in the fast-growth condition (doubling time 25 min). **f**, The same phase diagram is shown on a linear scale. Avg, average; WT, wild type. Source data

To explore and construct the MinD–MinE concentration phase diagram, we developed a variety of *E. coli* strains (Fig. 2 and Methods). Initially, we created two tunable CRISPR interference (tCRISPRi) strains^33^ capable of gradually repressing either the entire operon *minCDE* (SJ1696) or *minE* (SJ1697) (Fig. 2a). These repression strains allowed us to probe the MinD–MinE phase space either diagonally (SJ1696 for [MinE] = [MinD]) or vertically (SJ1697 for [MinD] = [MinD]_wt_), starting from the wild-type expression level with minimal off-target effects^33^ (Fig. 2a) Supplementary Table 1 lists the *E. coli* strains used in this study.

Furthermore, we constructed a dual-inducible strain (SJ1883) on the *E. coli* chromosome to enable the independent, gradient expression of *minCD* and *minE* (Fig. 2b). Specifically, we replaced the native promoter with our previously developed P_BAD_ system^33^ and inserted the P_LTet_ promoter^34^ between native *minD* and *minE*. To calibrate the P_LTet_ promoter under different growth conditions, we created an intermediate strain, SJ1935, which allowed the inducible expression of *minE* (Fig. 2b). Detailed dose-induction curves of the expression system can be found in Supplementary Information Section 1. These *min*-inducible strains enabled us to explore the MinD versus MinE concentration phase diagram for a concentration range around the wild-type level of more than two orders of magnitude.

To determine the Min protein concentrations, we employed fluorescence imaging and calculated the integrated fluorescence intensity normalized by the cell volume (Methods). To calibrate the fluorescence intensity to protein copy numbers, we extracted the MinC, MinD and MinE copy numbers from three published datasets based on mass spectroscopy^35^, ribosome profiling or ribosome sequencing (Ribo-Seq), which quantifies protein levels by counting actively translating ribosomes^36^, as well as a hybrid method that combines both approaches^37^ (Fig. 2c). These published datasets reported a wide range of protein copy numbers per cell, with MinC ranging between 100 and 600, MinD between 1,000 and 5,000, and MinE between 500 and 4,000 (Fig. 2c). The variations observed can be attributed to differences in growth conditions, strains and experimental methods. Among the three datasets, we used the Ribo-Seq and hybrid datasets for comparison with our fluorescence measurements. This choice ensured that we could use our cell size versus growth rate data^38^ to calculate the protein concentrations, because these two studies employed the same *E. coli* strain background (MG1655) as us (Fig. 2d). The trends for MinD and MinE concentrations with respect to the growth rate were consistent among the two datasets and also aligned with our imaging-based measurements (Fig. 2d, Supplementary Fig. 2b and Supplementary Information Section 3). Finally, to construct our phase diagram, we chose to convert our fluorescence intensity measurements into protein concentrations using the Ribo-Seq data for the fastest growth condition^36^, as their per-cell protein copy numbers agreed more closely with the mass spectrometry data (Fig. 2d,e, Supplementary Information Section 3.2 and Supplementary Fig. 3).

We describe strain construction, calibration (conversion of fluorescence to protein concentrations) and controls in detail in Methods and Supplementary Information Sections 1–5.

## The MinD–MinE phase diagram: oscillations were robust to MinD and MinE concentrations

With the four titratable strains we developed, we were able to explore the [MinD] versus [MinE] phase diagram by precisely controlling the expression levels of *minCDE* (SJ1696 and SJ1883), *minCD* (SJ1883) and *minE* (SJ1697, SJ1883 and SJ1935). This allowed us to probe a range of Min protein levels spanning about two orders of magnitude. To investigate the impact of growth physiology on Min pattern formation, we selected two growth conditions with average doubling times of 55 min (slow; minimal glycerol media) and 25 min (fast; synthetic rich media) at 37 °C. To construct the phase diagram and identify different pattern-formation regimes, we tracked the dynamics of GFP-MinD (an N-terminal monomeric superfolder GFP fusion; we refer to it as GFP for brevity) at various MinCDE levels using time-lapse microscopy and classified the observed dynamic patterns (Fig. 2e, right).

The phase diagram reveals a large region where cells exhibit dynamic Min patterns. This pattern-formation regime can be further divided into two categories: cells that divide normally, showing a wild-type phenotype, and those that develop a more filamentous phenotype. Interestingly, the pattern-formation regimes show a distinct vertical elongation in the [MinD]–[MinE] parameter space (Fig. 2e,f). This asymmetry was unexpected, as we initially anticipated oscillations to require a [MinD]/[MinE] ratio of 1, reflecting the presumed co-regulation of *minCDE* gene expression.

Notably, we found that wild-type pole-to-pole oscillations remained robust across a wide range of Min concentrations, with at least sixfold changes in MinD concentrations and tenfold changes in MinE concentration, well beyond physiological fluctuation levels.

## The MinD–MinE phase diagram: the wild-type *minCDE* expression levels are near resource-optimal

Our phase diagram revealed that the wild-type expression levels of *minD* and *minE* are generally within a ×2 range of the minimal protein concentrations required for oscillation, regardless of whether the cells were in slow- or fast-growth conditions. Given that the magnitude of gene expression noise typically falls within the range of 10% to 20% coefficient of variation^39–41^, the Min protein levels at the wild-type expression levels are nearly resource-optimal for sustaining oscillations.

Even more so, the constitutively expressed genes exhibited decreasing protein concentrations as the nutrient-imposed growth rate was increased (Fig. 2d)^34^. Consequently, the maximal achievable growth rate of *E. coli* sets the minimal protein concentrations occurring in wild-type cells growing under distinct conditions. Given that the concentration of MinD under fast-growth conditions is positioned at the onset of pattern formation, the expression of the Min operon seems to be strategically adjusted to be resource-optimal but to allow Min oscillations under all growth conditions while also buffering fluctuations in protein concentrations. This finely tuned balance of the Min levels in the wild-type cells is in contrast to the large oscillation regime that enables robustness of the Min system for a wide range of Min protein concentrations.

## The MinD–MinE phase diagram: loss of pattern formation at high expression of *minD* or *minE*

When the expression levels of *minCD* and *minE* deviated considerably from the wild-type level, the dynamic patterns observed in the system eventually vanished. We identified two distinct ‘no-pattern’ regimes depending on the relative ratio [MinE]/[MinD] (Fig. 2e). The origins of these regimes can be explained as follows.

In the regime where [MinE]/[MinD] ≫ 1, the presence of an excessive amount of MinE proteins strongly inhibited the binding of MinD to the cell membrane throughout the cell. Consequently, most of the MinD proteins remained in the cytoplasm and were unable to inhibit the formation of Z-rings at the cell poles, which are crucial for the division at mid-cell. As a result, mini-cells formed within this regime.

Conversely, in the regime where [MinE]/[MinD] ≪ 1, the insufficient amount of MinE protein led to the binding of MinD proteins all over the inner membrane, thus recruiting MinC from the cytoplasm. The excessive accumulation of MinD-MinC complexes on the inner membrane prevented the formation of the Z-ring, resulting in the filamentation of the cells.

## MinE conformational switch provides robustness to the Min system

**Fig. 3:**
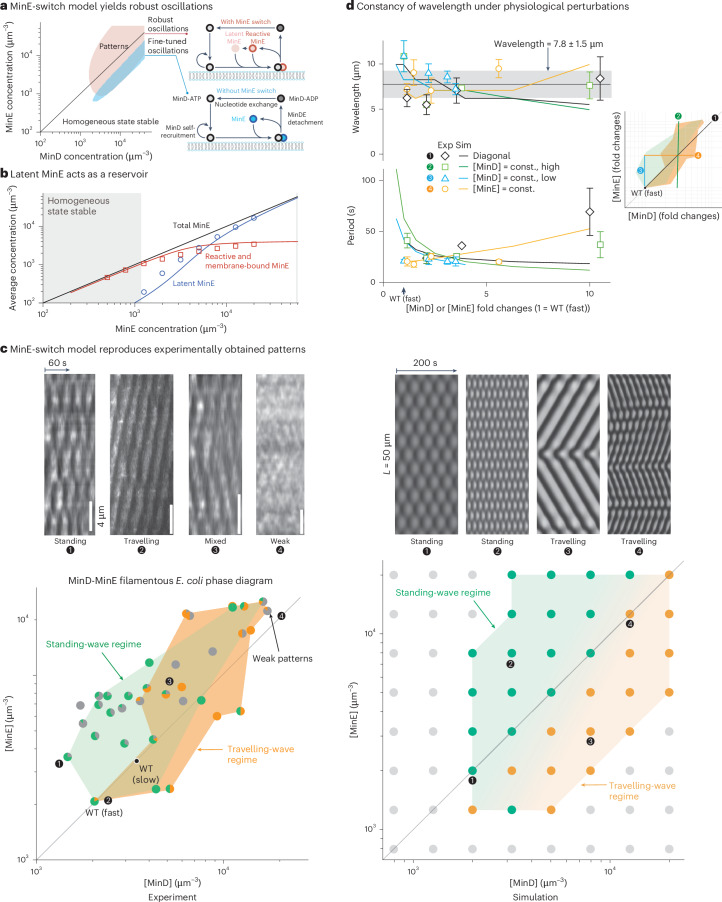
MinE-switch model and comparison between the experimentally obtained and the theoretically simulated phase diagrams. **a**, Comparison between the minimal model without MinE switch (skeleton model) and the MinE-switch model based on a linear stability analysis of the homogeneous steady state (Supplementary Information Section 10). Patterns grow out of small inhomogeneous perturbations of the uniform steady-state concentrations only within the shaded regions. The model without the MinE switch considers the attachment–detachment cycle of MinD at the membrane (schematically shown as a lipid bilayer in blue and grey), as explained in Fig. 1a. The MinE-switch model has another latent MinE conformation that binds weakly to membrane-bound MinD-ATP. The resulting region of pattern formation covers a much larger area of the [MinD] versus [MinE] phase diagram compared to that predicted by the model without the MinE switch, which permits patterns to form only in a narrow diagonal stripe. **b**, The reservoir effect of the latent MinE state. The concentrations of latent MinE (blue) and MinE in the reactive and MinDE states (red) are compared to the total MinE concentration (black). The concentrations in the homogeneous steady state analysed in **a** are depicted as lines. The concentrations in the numerical pattern simulations (cf. **c** and **d**) averaged over the whole simulation domain and 1,000 s of simulated time are shown as symbols. The grey-shaded region denotes the concentrations at which the homogeneous steady state is stable. The total MinD concentration was fixed to 6,000 µm^−3^. **c**, ‘Filamentous’ Min phase diagram. The experimental phase diagram (left) consists of two datasets: filamentous cells from the phase diagram in Fig. 2 and normal-sized cells treated with cephalexin to induce filamentation. In filamentous cells, the type of pattern formation was discernible as standing waves (green), travelling waves (orange), and mixed or weak patterns (grey) with a gradual crossover between different pattern-formation regimes (Supplementary Videos 1–6). The small pie charts represent the number of cells in a given condition with the associated patterns. The experimental phase diagram compares well with the phase diagram obtained with the MinE-switch model (right; Methods). The grey dots (right) denote concentration combinations not resulting in dynamic patterns in the simulations. Representative kymographs of the cross-sectional average MinD pattern at different [MinD] and [MinE] levels from both experiments and simulation are shown in greyscale at the top. **d**, Wavelength and period of the patterns obtained from experiments (mean ± s.d.; symbols) and simulation (lines). The Min protein pattern formation shows wavelength constancy (~8 µm) over a large range of Min protein concentrations (top). The wave period decreases with increasing [MinE] and increases with increasing [MinD] in the simulations, with less clear trends in the experiments (bottom). The experimental data and simulations were obtained (approximately) along four cross sections of the phase diagram (right). The simulations were performed at (1) equal MinD and MinE concentrations, (2) a fixed MinD concentration of 6,000 µm^−3^, (3) a fixed MinD concentration of 2,000 µm^−3^ and (4) a fixed MinE concentration of 6,000 µm^−3^. In the experiment, the concentrations were measured relative to the fast-growth wild-type concentration levels (arrow). const., constant; Exp, experimental data; Sim, simulated data. Source data

We describe the Min protein patterns using a reaction–diffusion model (Fig. 3a, ‘Reaction–diffusion model’ in Methods and Extended Data Table 1) based on the bimolecular reactions proposed in the biochemical literature^10,21,24,26,27^. In this model, MinD-ATP, the active form of MinD, binds to the cell membrane and recruits more MinD-ATP molecules from the cytosol to the membrane. Membrane-bound MinD also recruits cytosolic MinE, forming a MinDE complex where MinE stimulates the ATPase activity of MinD. Upon ATP hydrolysis, both inactive MinD (MinD-ADP) and MinE detach from the membrane^42–44^. In the cytosol, MinD-ADP is reactivated through nucleotide exchange.

Additionally, we incorporated the conformational switch of MinE between its reactive state and the latent state induced by MinD^10,26^. In the reactive state, MinE is readily recruited by MinD (Fig. 3a). Conversely, in the latent state, MinE has a weak affinity for MinD, as its interaction sequence with MinD is conformationally buried. The minimal network without the MinE switch (skeleton model) qualitatively reproduces the pole-to-pole oscillations observed in wild-type cells and the dependence of the dynamic patterns on cell geometry^24,45^ as well as the transition from travelling waves to standing waves and to chaos in vitro^46,47^. However, a linear stability analysis of the homogeneous protein distribution revealed that patterns formed only within a narrow range of the [MinE]/[MinD] ratio (Fig. 3a)^24^, in clear contrast to our experimental observations (Fig. 2e).

The model incorporating the MinE switch explains the robustness of pattern formation observed in in vitro experiments^26^. The underlying mechanism lies in the latent state acting as a reservoir that buffers high concentrations of MinE and enables pattern formation, even at high [MinE]/[MinD] ratios. We simulated this MinE-switch model within a spherocylindrical cellular geometry (Supplementary Information Sections 8 and 9). As depicted in Fig. 3a, the switch model adequately accounts for the robustness of pattern formation in vivo for over one order of magnitude in MinE concentration. Figure 3b shows that most of the MinE resides in the reactive and membrane-bound (MinDE) states at low MinE concentration. At higher MinE concentrations, this amount levels off, and most of the MinE is buffered in the latent state, which reveals the reservoir effect of the latent state. The pattern-forming regime seems to extend to higher MinD concentrations in the model compared to the experiments, which we attribute to the low number of experimental measurements in this region of the phase diagram and the aggregation of MinD not being considered in the model (Supplementary Information Section 7). Frequently, cells with very high MinD levels did not grow.

## The MinD–MinE phase diagram: the Min system shows rich dynamic patterns in vivo

As we increased the expression levels of *minCD* beyond the region of wild-type growth (Fig. 2e), we observed the filamentation of cells due to the inhibition of cell division. In these elongated cells, dynamic patterns emerged that are distinct from what is seen in wild-type cells. The short length of the wild-type cells restricts the type of patterns formed, as only half a wavelength of a pattern is allowed. By contrast, the patterns can develop in filamentous cells over several wavelengths, so that they show several domains of high and low density. Consequently, the patterns observed in the phase diagram (Fig. 2e) were influenced by two intertwined processes: (1) the formation of different pattern types depending on the concentration of the Min proteins and (2) the length at which cells divide. To decouple pattern formation from cell size, we re-explored the phase diagram, analysing exclusively filamentous cells. We did this by treating cells with cephalexin to suppress cell division in those regions of the phase diagram where cells do not become filamentous (Fig. 3c and ‘Growth conditions and media’ in Methods). Notably, we discovered two types of patterns: travelling waves and standing waves (the classification is described in Methods).

At a high [MinE]/[MinD] ratio, the Min proteins formed standing-wave patterns. In standing waves, the GFP-MinD signal localized periodically at fixed points along the cell length (Fig. 3c, kymograph labelled ‘standing’). These patterns can be viewed as neighbouring pole-to-pole oscillations. Because of the defined wavelength of the Min oscillations (see below), in cells longer than the pattern wavelength, the proteins oscillated between several ‘stripes’ rather than between the poles. In addition, travelling-wave patterns formed at a low [MinE]/[MinD] ratio. The travelling waves exhibited a wavefront that moved in a unidirectional manner along the cell (Fig. 3c, kymograph labelled ‘travelling’). Importantly, mixed patterns combining standing-wave and travelling-wave phenomenology occurred for many concentrations between the standing- and travelling-wave regions. First, this indicates that transitions between patterns are not abrupt but rather continuous. Second, inhomogeneities in the membrane or the cytosol due to the nucleoids, for instance, might modulate the patterns and lead to less clear distinctions than in in vitro experiments.

Previous studies, for instance by Raskin and de Boer^31^, also observed the standing-wave behaviour of MinD in FtsZ filamentous cells. Moreover, the arrangement of the phase diagram in our study is like the behaviour observed in recent in vitro experiments on pole-to-pole oscillations and circular travelling-wave patterns in spherical microdroplets^48^ but contrasts with observations on supported lipid bilayers, even at a low bulk height^46^.

Although standing waves have been observed systematically in vivo, travelling waves have been observed previously only at intermediate lengths and in cyanobacteria^49^. By contrast, a large variety of dynamic patterns occur in vitro, including travelling waves, ‘mushrooms’^15^, ‘bursts’^15,50^, standing waves, chaos^46^, ‘amoebas’^50^ and stationary patterns^51^. Systematically observing travelling-wave patterns connects in vivo Min pattern formation to the variety of patterns observed in vitro.

## The MinE-switch model captures the various types of patterns observed in filamentous cells

The success of the switch model in reproducing the robustness of pattern formation prompted us to investigate whether it also explains the pattern types observed in the experimental phase diagram (Fig. 3c). Notably, our simulations using the MinE-switch model closely reproduced the pattern-formation regimes observed in filamentous cells (see Supplementary Fig. 26 for the simulated phase diagram for wild-type cells). We obtained standing-wave patterns at high [MinD] and travelling-wave patterns at low [MinD]. The transition between the two regimes was gradual, consistent with our experimental observations (Fig. 3c). The close resemblance of the experimental phase diagram to the modelling phase diagrams (Fig. 3c) supports the notion that the MinE-switch model encompasses the primary interactions governing Min pattern formation in vivo.

Although the MinE switch enables robust pattern formation across a wide range of model parameters, the arrangement of distinct pattern types in the phase diagram (and the oscillation period and wavelength measurements, see below) constrains the parameter selection (Methods and Supplementary Information Section 11).

## Period and invariant wavelength of Min patterns

We also quantified the oscillation period and wavelength of the patterns. The simulations with filamentous cells show that the oscillation period decreased with increasing MinE concentration and increased with increasing MinD concentration (Fig. 3d). Heuristically, the reason for this is that a reduced (relative) amount of MinE compared to MinD decreases the rate with which sufficient MinE is recruited to a MinD domain to induce its detachment. As a result, the oscillation period increases for increased MinD:MinE ratios.

The oscillation period quantitatively reproduces the measurements in filamentous cells for at least threefold increased concentrations compared to the wild-type concentration levels (Fig. 3d). The measurements show an increase in the oscillation periods at tenfold increased concentrations that is not reproduced by the MinE-switch model. Similarly as for the reduced region of pattern formation (Fig. 3c), the increased experimental oscillation period at high MinD (and MinE) concentrations compared to the simulations may be due to the aggregation of MinD and a thereby reduced detachment rate of MinD from the membrane.

Throughout the entire phase diagram, the pattern wavelength remained notably invariant at 7.8 µm on average with 20% coefficient of variation when the MinD and MinE concentrations were varied tenfold in two growth conditions (Fig. 3d). This observation is supported by our numerical analysis of the switch model, which also demonstrated that the wavelength varied only weakly. Close to the onset of pattern formation at low MinD concentration, the wavelength and oscillation period were determined through a linear stability analysis due to the supercritical nature of the onset^52^. Further inside the region of instability, the linear stability analysis was not informative (Supplementary Information Section 10).

## Spatial distribution of MinD and implications on cell division

**Fig. 4:**
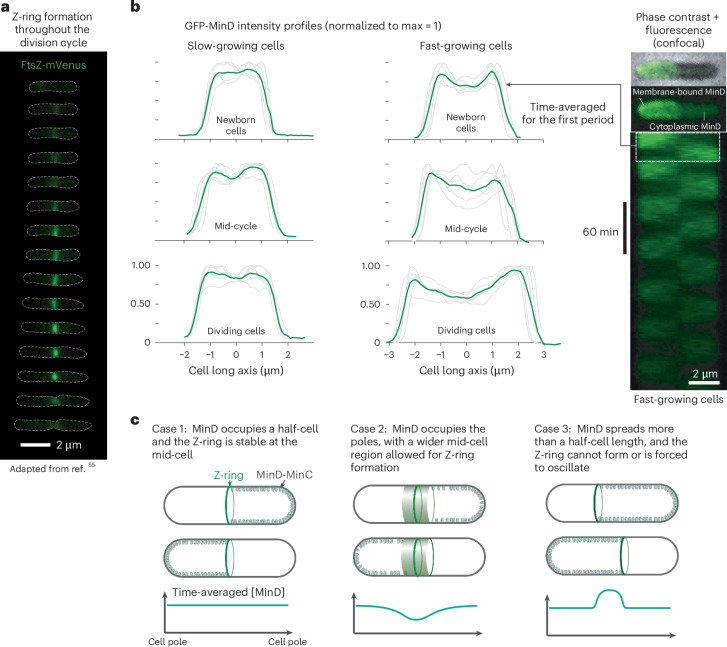
Time-averaged GFP-MinD distribution in fast- and slow-growth conditions. **a**, Growth in MOPS glycerol + 11 amino acids (doubling time ~1 h). The Z-ring (fluorescence signal of FtsZ-mVenus shown) forms at the mid-cell (cell perimeter shown as dashed lines) throughout the division cycle (top to bottom). The data were obtained in the experiments of ref. ^55^. **b**, Time-averaged GFP-MinD intensity profile in fast- and slow-growth conditions in early, mid and late division cycles. These profiles were obtained from confocal images (right) by combining and averaging traces (green) from five cells for each condition. Individual traces are shown in grey. **c**, Three illustrative examples of MinD spatio-temporal patterns and the expected time-averaged distributions along the long axis of the cell. The green-shaded regions (case 2) symbolize a possible variation in the Z-ring position. Panel **a** reproduced with permission from ref. ^55^, Cell Press.

One of the long-standing questions in bacterial cell physiology is what guides the Z-ring to the mid-cell with over 95% precision^53^. Until recently, the consensus was that the Min system prevents mini-cell formation, whereas nucleoid occlusion positions the Z-ring at mid-cell once the duplicated nucleoids separate before division^54^. However, our previous work shows that the Z-ring forms at the mid-cell almost immediately after division, even while the nucleoid is still replicating^55^ (Fig. 4a). This raises the question of whether the Min system may also play a role in guiding the Z-ring to the mid-cell.

A previous theoretical study indicated that MinD (and, thus, MinC) primarily localizes to the cell poles, producing a time-averaged dip in Min protein concentration at the mid-cell along the long axis^23^. Using extensive time-lapse imaging from this study under both slow- and fast-growth conditions, we plotted the time-averaged GFP-MinD distribution across early, mid and late division cycles in both conditions. Surprisingly, we often found no clear dip in GFP-MinD at the mid-cell. Instead, kymographs revealed that GFP-MinD consistently occupied nearly half of the cell along the long axis, such that the Z-ring could, perhaps, stay stably at the mid-cell, as illustrated in Fig. 4c (case 1). Occasionally, we also observed a mild dip at the mid-cell (case 2), but we never found GFP-MinD occupying more than half of the cell (case 3).

Whether the MinD pattern may guide the Z-ring to the cell centre across diverse growth conditions is beyond the scope of this work. However, we believe that the absence of a pronounced and narrow dip motivates future studies on how *E. coli* achieves a notably symmetric division.

## Outlook

The pole-to-pole oscillation of Min proteins is an important example of dynamic pattern formation in biology, which has been extensively studied across disciplines for decades. Yet, several fundamental questions explored in this work had remained open. Our findings emerged from reframing these questions within a physiological framework, and we believe that this perspective can be applied to other long-standing questions in biological physics.

For example, an intriguing finding from our combined study is the invariant wavelength of Min patterns around 8 µm, irrespective of *minCDE* concentrations and growth physiology. Does this invariance provide further robustness to symmetric cell division in *E. coli* at their wild-type length? Importantly, the mid-cell position of the Z-ring is robust only if the Min proteins oscillate between the poles and do not form a standing wave with a MinD node at mid-cell. The transition of the pattern into such a standing wave occurs when the cell length approaches the pattern wavelength. Intriguingly, the average size of the fastest-growing (and, thus, largest) *E. coli* cells at division is approximately 8 µm long^53^. Moreover, the invariance of the Min wavelength could have other biological implications, such as cell-size control^56,57^, which is an exciting avenue for further research. Finally, although a linear stability analysis accounted well for the wavelength of weakly nonlinear patterns, understanding the basis of wavelength selection far away from onset and in the nonlinear regime is a critical open question.

In a broader context, bacteria use a plethora of mechanisms for the spatial control of cell division. We have identified the MinE switch as a robustness module through Min oscillations in the cell division control of *E. coli*. Because accurate cell division is pivotal for proliferation, it is essential to explore molecular mechanisms that ensure robust spatial control in bacteria using different strategies. Moreover, there is a fundamental balance between resource efficiency and robustness against fluctuating gene expression levels. Comprehending the interplay between different spatial control strategies, their robustness and protein-concentration homeostasis will be key to understanding the accurate selection of division sites across generations under various conditions.

## Methods

### Strains used for the MinD–MinE phase diagram

To measure the dynamic Min patterns and quantify the Min protein concentrations using imaging, we N-terminally fused *msfGFP* to *minD* and inserted a transcriptional reporter *mCherry* downstream of *minE*. The fused *msfGFP* enabled us to monitor the Min patterns, and we were able to place points in the phase space without modifying MinE. We did not use MinE fluorescence fusion proteins as they are known to be not fully functional^51,58^.

All our strains used in this work were derived from MG1655 (ref. ^59^). SJ1695 has an N-terminus fusion of msfGFP^60^ to *minD* and mCherry^61^ as a transcriptional reporter encoded downstream of *minE* in our tCRISPRi strain without signal guide RNA^33^. Construction of the gradient repression strains SJ1696 and SJ1697 was based on SJ1695 with signal guide RNA sequences targeting *minC* (SJ1696) and *minE* (SJ1697).

We constructed the *minE* inducible strain SJ1935 from SJ1695 by inserting an inducible promoter P_LTet_ between *minD* and *minE*, the *rrnB* terminator between *minD* and P_LTet_, and the repressor expression system P_LTet_::tetR at the yfdG locus. We subsequently built the dual-inducible strain SJ1883 by replacing the native promoter of the Min operon with the P_BAD_ promoter^33^. Strain construction details are available in Supplementary Information Section 5.

### Growth conditions and media

Strains were kept at −80 °C. Glycerol stocks were immediately streaked on a lysogeny broth (LB) medium plate with proper antibiotics and inducer concentrations to allow recovery at the wild-type Min protein levels. Specifically, we used 50 µg ml^−1^ spectinomycin for SJ1695, SJ1696 and SJ1697; 50 µg ml^−1^ kanamycin plus 80 ng ml^−1^ cTc for SJ1935; and 50 µg ml^−1^ kanamycin, 25 µg ml^−1^ chloramphenicol, 80 ng ml^−1^ cTc plus 0.05% (w/v) arabinose for strain SJ1883. Single colonies were picked and inoculated into a 1 ml LB pre-culture for overnight growth in a 37 °C water bath in the same conditions as described above for the LB nutrient plate.

For the fast-growth condition experiments, we back-diluted the overnight culture 1:1,000 into MOPS-rich EZ 0.2% glycerol^38,62^ with the same amount of antibiotics added. Gradient expression of *minCDE* was achieved by adding differential amounts of inducers after 1:1,000 back-dilution in strain SJ1883 (both arabinose and cTc) or SJ1935 (cTc only). Gradient repression of *minCDE* or *minE* was achieved by adding differential amounts of arabinose after 1:1,000 back-dilution in strain SJ1696 or SJ1697.

For the slow-growth experiments, we used minimal MOPS, minimal glycerol 0.2% media^38,62^. There was a considerable lag period between the back-dilution and the start of growth when using 1:1,000 dilution. After starting an LB culture, we left the cells overnight in MOPS minimal glycerol with 1:1,000 dilution. For each back-dilution step, the procedure was the same as for the fast-growth experiments.

All experiments but one were performed at 37 °C. To generate the profiles in Fig. 4, we used our confocal set-up, which was kept at 30 °C. For that experiment, the cells were given 2 h to equilibrate at 30 °C in liquid culture before imaging.

To create the diagram in Fig. 3b, we needed to decouple the observed patterns from the cell geometry. This was because for cells with a length close to the wild type, the end cap could have affected the observed pattern. Some cells (those with high MinD levels) were already filamentous and forming patterns, so we could use them in Fig. 3b as is. Other cells were naturally short, and we needed to induce filamentation. To do this, we treated the cells with a sublethal concentration of cephalexin (10 µg ml^−1^) and allowed them to grow for 70–80 min. We imaged the cells immediately before and after treatment and took note of the observed patterns (‘Dynamic pattern classification’).

The inducer concentrations and growth conditions for each point in the phase diagram are available in the Source data for Fig. 2 and Fig. 3c, respectively.

### Imaging sample preparation

We started preparing for imaging when the back-diluted cells reached OD600 = 0.3. We used a growth-medium-based 2% agarose pad (Sigma A9539-500G) on a WillCo dish (WillCo-dish KIT-5040), which was pre-warmed in the warm 37 °C chamber before adding agar pads. We prepared different agarose pads on the WillCo dish by varying the inducer concentrations to match the inducer concentrations in the liquid cultures (except that no antibiotics were added for agar-pad imaging). The perimeter of the coverslip was sealed with dielectric grease to prevent the agar pad from drying during imaging at 37 °C.

### Imaging and microscopy

Fluorescence image acquisition was conducted on a Nikon Ti-E inverted microscope using a Prime95B sCMOS camera. For the first 13 experiments, we excited msfGFP and mCherry, and we used coherent OBIS 488 nm (100 mW) and 561 nm (50 mW) lasers, respectively, at 100% power with 200 ms exposure time. For later experiments, we used a Lumencore Spectra in cyan and green, respectively. Each imaging session included a wild-type agar pad for normalization. The msfGFP was imaged every 2 s to capture the dynamics of MinD proteins. To obtain statistics for the fluorescence signals, we imaged both msfGFP and mCherry every 5 min with several fields of view. On average, we analysed 50–500 cells for each inducer condition for the fluorescence intensity analysis. The fluorescence analysis and videos were acquired on the same agar pad but from separate sets of cells due to experimental limitations. Confocal image acquisition was also conducted on a Nikon Ti-E inverted microscope with a Prime95B sCMOS camera, Yokogawa CSU-W1 confocal unit and a Vortran Stradus VersaLase 8 (at 488 nm and 561 nm at 100% intensity) with 200 ms exposure. To control for optical differences, each experiment (set of agar pads) recorded an image of the wild-type strain SJ1695 under identical conditions.

### Image processing and data analysis

We used Omnipose^63^ for cell segmentation during cell growth on the agar pads. After cell segmentation, we quantified the protein concentration using the integrated total fluorescence intensity normalized by the cell volume. We computed the volume by approximating the cell as a cylinder with two hemispherical caps. To categorize patterns in filamentous cells, we generated their kymographs. To do this, we segmented cells using Omnipose^63^ and then determined their medial axis using skeletonization. At each point in the skeleton, we averaged the MinD-msfGFP intensity over a 0.4-μm disc centred at each point. Finally, we corrected for arc length to remove spatial distortion. To generate intensity profiles of wild-type-length cells, kymographs were built using KymoResliceWide v.0.5 plugin for ImageJ (https://github.com/ekatrukha/KymoResliceWide). We then averaged the intensity over only the first period to reduce the effects of photobleaching on the signal-to-noise ratio.

### Dynamic pattern classification

To create the Min phase diagrams (for example, Fig. 2), it was necessary to categorize whether cells form dynamic Min patterns at a particular *minD* and *minE* expression level (or an inducer condition). For the filamentous phase diagram in Fig. 3, it was necessary to categorize the patterns as standing, travelling, mixed or weak within a given field of view. For each agar pad, we sampled ten cells for the statistics (for movies with a low density of cells, we categorized all cells that were available). A per-agar pad breakdown is available in Supplementary Tables 1 and 2. Categorizations were based on kymographs. The kymographs used for the filamentous phase diagram are available in the Source data for Fig. 3c. Owing to the volume of the full dataset, all other kymographs are available upon request.

For the Min phase diagram in Fig. 2, if half or more of the sampled cells exhibited any kind of pattern, we deemed the regime to be pattern-forming. In non-pattern-forming cells with high MinD-msfGFP, we frequently observed fluorescent patches and occasionally transitions from non-pattern-forming to pattern-forming fluorescence (Supplementary Video 7).

For the filamentous phase diagram in Fig. 3, our goal was to produce a phase diagram that is independent of cell length, to allow a more accurate comparison to theory. To achieve this, we examined agar pads where the median cell length was over 8 μm (which is typically more than ×2 longer than the dividing cells in fast-growth conditions) and agar pads containing cells after cephalexin treatment. Using kymographs, we classified the pattern-forming cells as travelling, standing, weak pattern or mixed travelling and standing. These classifications are available in the source data for Fig. 3c.

A travelling wave was indicated by long, diagonal lines of fluorescence in the kymograph, a standing wave by a chequerboard pattern (as shown in Fig. 3c) and the mixed regime by a mixture of the two. More formally, standing-wave patterns are symmetric under the reflection of the spatial coordinate (the cell long axis). By contrast, travelling waves propagate, which breaks reflection symmetry. We used this difference in symmetry to distinguish between travelling- and standing-wave patterns. Importantly, travelling waves can travel in opposite directions in different spatial regions of the cell, leading to ‘source’ and ‘sink’ defects for the travelling waves (see the simulated travelling-wave kymographs in Fig. 3c). Similarly, the cell poles, which act as boundaries, can alter the patterns close to the poles by, for instance, stopping the propagation of travelling waves. As described in the main text, the transition from standing to travelling waves was continuous. We frequently observed cells with an obvious spatial structure reminiscent of the Min patterns, but we were unable to classify these as either standing or travelling. These were marked as weak patterns (Supplementary Video 5).

For the simulated phase diagram, the classification into standing- and travelling-wave patterns was based on the simulated kymographs of the total density of membrane-bound MinD. These simulated kymographs are free of noise and could, therefore, be classified algorithmically (Supplementary Information Section 11.1). Again, travelling waves give rise to diagonal lines of high (low) density, whereas standing waves give rise to disconnected domains of high or low MinD membrane density (Fig. 3c). To distinguish both pattern types, we counted the number of high- and low-density domains per oscillation period and wavelength (Supplementary Information Section 11.1). The threshold between both types corresponds to a pattern showing a travelling wave in approximately half of the cell and a standing wave in the opposite half. The oscillation period and pattern wavelength were determined by Fourier analysis. The positions of the maximum of the spatially averaged power spectrum and temporally averaged structure factor were used as measures for the period and wavelength, respectively (Supplementary Information Section 11.1).

### Reaction–diffusion model

We modelled the geometry of cells as a spherocylinder composed of a cylinder of length *L* = 50 μm for filamentous cells or *L* = 3 μm for wild-type-length cells (Supplementary Information Section 12) and radius *R* = 0.5 μm with two hemispherical caps at its ends with the same radius. The interior of the spherocylinder represents the cytosol. In the switch model, the cytosol contains the inactive MinD-ADP, active MinD-ATP, reactive MinE and latent MinE states (Fig. 3a). The evolution of the concentration fields *c*_DD_(**x**, *t*) (MinD-ADP), *c*_DT_(**x**, *t*) (MinD-ATP), *c*_E,r_(**x**, *t*) (reactive MinE) and *c*_E,l_(**x**, *t*) (latent MinE) is modelled by the reaction–diffusion equations

$${\partial}_{t}{c}_\mathrm{DD}(\mathbf{x},t)={D}_\mathrm{D}\nabla^{2}{c}_\mathrm{DD}-\lambda {c}_\mathrm{DD},$$

$${\partial}_{t}{c}_\mathrm{DT}(\mathbf{x},t)={D}_\mathrm{D}\nabla^{2}{c}_\mathrm{DT}+\lambda {c}_\mathrm{DD},$$

$${\partial}_{t}{c}_\mathrm{E,r}(\mathbf{x},t)={D}_\mathrm{E}\nabla^{2}{c}_\mathrm{E,r}-\mu {c}_\mathrm{E,r},$$

$${\partial}_{t}{c}_\mathrm{E,l}(\mathbf{x},t)={D}_\mathrm{E}\nabla^{2}{c}_\mathrm{E,l}+\mu {c}_\mathrm{E,r}.$$

Here, *D*_D,E_ denote the cytosolic diffusion coefficients of MinD and MinE, λ the nucleotide exchange rate, and μ the rate of the conformational MinE switch. The diffusion coefficients and reaction rates are given in Extended Data Table 1. The surface of the spherocylinder represents the inner cell membrane. The surface concentration fields of membrane-bound MinD proteins *m*_d_(**x**, *t*) and MinDE complexes *m*_de_(**x**, *t*) follow the equations

$${\partial}_{t}{m}_\mathrm{d}(\mathbf{x},t)={D}_\mathrm{d}{\nabla^{2}}_\mathrm{LB}{m}_\mathrm{d}+\left({k}_\mathrm{D}+{k}_\mathrm{dD}{m}_\mathrm{d}\right){c}_\mathrm{DT}|_\mathrm{m}-\left({k}_\mathrm{dE,r}{c}_\mathrm{E,r}|_\mathrm{m}+{k}_\mathrm{dE,l}{c}_\mathrm{E,l}|_\mathrm{m}\right){m}_\mathrm{d},$$

$${\partial}_{t}{m}_\mathrm{de}(\mathbf{x},t)={D}_\mathrm{de}{\nabla^{2}}_\mathrm{LB}{m}_\mathrm{de}+\left({k}_\mathrm{dE,r}{c}_\mathrm{E,r}|_\mathrm{m}+{k}_\mathrm{dE,l}{c}_\mathrm{E,l}|_\mathrm{m}\right){m}_\mathrm{d}-{k}_\mathrm{de}{m}_\mathrm{de}.$$

Here, the Laplace–Beltrami operator $\nabla^{2}_{\rm{LB}}$ is used to describe diffusion along the (curved) membrane. The rate constants are denoted by *k* with the subscripts referring to the respective reaction. The subscripts _dE,r_ and _dE,l_ denote the recruitment rates of reactive and latent MinE by membrane-bound MinD, respectively. For the rate constants, we refer again to Extended Data Table 1. The notation ⋅|_m_ denotes the evaluation on the membrane (on the surface of the spherocylinder). The attachment of proteins onto the membrane implies a depletion of the cytosolic protein concentrations, whereas detachment from the membrane increases the protein concentrations in the cytosol. These interactions are described by reactive boundary conditions:

$$\mathbf{n}\cdot {D}_\mathrm{D}\nabla {c}_\mathrm{DD}{|}_\mathrm{m}={k}_\mathrm{de}{m}_\mathrm{de},$$

$$\mathbf{n}\cdot {D}_\mathrm{D}\nabla {c}_\mathrm{DT}{|}_\mathrm{m}=-\left({k}_\mathrm{D}+{k}_\mathrm{dD}{m}_\mathrm{d}\right){c}_\mathrm{DT}|_\mathrm{m},$$

$$\mathbf{n}\cdot {D}_\mathrm{E}\nabla {c}_\mathrm{E,r}|_\mathrm{m}=-{k}_\mathrm{dE,r}{c}_\mathrm{E,r}|_\mathrm{m}{m}_\mathrm{d}+{k}_\mathrm{de}{m}_\mathrm{de},$$

$$\mathbf{n}\cdot {D}_\mathrm{E}\nabla {c}_\mathrm{E,l}|_\mathrm{m}=-{k}_\mathrm{dE,l}{c}_\mathrm{E,l}|_\mathrm{m}{m}_\mathrm{d},$$

where **n** denotes the outward-pointing unit normal vector of the surface.

The minimal model without the MinE switch^24^ (skeleton model) is recovered from the above equations by summing the reactive and latent MinE states *c*_E_ = *c*_E,r_ + *c*_E,l_ and setting the recruitment rates for both states to the same value *k*_dE,r_ = *k*_dE,l_ = *k*_dE_.

Owing to the small radius of the spherocylinder, the cytosolic concentration gradients were negligible and the dynamics could be approximated by a reduced one-dimensional model that assumes that the cytosolic concentrations are constant within a cross section of the spherocylinder (Supplementary Information Section 9). The dynamics of the concentration fields integrated over each cross section evolved on the one-dimensional line, and we modelled the cell poles as no-flux boundary conditions constraining the evolution to an interval of length *L* = 50 μm. This reduced dynamics was used in an extensive study of the rate constants in the switch model. Moreover, the reduced dynamics on the infinite line was used for the linear stability analysis (Fig. 3a and Supplementary Information Section 10).

### Parameter choice

The reaction parameters employed in ref. ^26^ for the switch model give rise to travelling waves in most of the phase diagram in contrast to the experimentally observed standing- and travelling-wave regions (Supplementary Fig. 20). The parameter set used to describe the experimentally determined phase diagram (Fig. 3b) is given in Extended Data Table 1, which is the result of a broad parameter study because the reaction rates are unknown experimentally (Supplementary Information Section 11.2).

We used the cytosolic diffusion coefficients for MinD and MinE that were determined experimentally in vivo using fluorescence correlation spectroscopy^64^. Estimates for their membrane diffusion constants were obtained in in vitro measurements^65^. Because the cell membrane of a live bacterium is crowded with diverse molecules, we chose low membrane diffusion constants comparable to the values measured in vitro at high MinD densities on the membrane. We increased the value compared to the value used in the minimal model in ref. ^24^ to reproduce the experimentally determined pattern wavelength better. However, the wavelength changed only weakly with the membrane diffusion constant (Supplementary Fig. 24).

The nucleotide exchange rate *λ* = 5 s^−1^ was chosen to meet the lower bound determined in ref. ^64^. The other reaction rates were determined to reproduce the experimental phase diagram. To match the overall region of pattern formation, following Denk et al.^26^, a small value was chosen for the recruitment rate *k*_dE,l_ of the latent MinE state compared to the rate *k*_dE,r_ for the reactive MinE state. This allowed the formation of a MinE reservoir. The switching rate *μ* = 20 s^−1^ was chosen to describe a fast switch. It was reduced compared to the value *μ* = 100 s^−1^ employed in ref. ^26^ to enlarge the phase-space region with standing-wave patterns (Supplementary Fig. 22). The hydrolysis rate *k*_de_ sets the threshold for pattern formation at low total MinE concentrations, and it was fixed to match the experimentally observed oscillation frequency of the Min patterns (Supplementary Fig. 25). The linear MinD attachment rate *k*_D_ influences the onset of pattern formation at low total MinD and MinE concentrations. The MinD self-recruitment rate *k*_dD_ sets the pattern threshold at low total MinD concentrations and the pattern wavelength. Both *k*_D_ and *k*_dD_ were then fixed such that the pattern types and wavelengths matched in the phase diagram (Supplementary Figs. 21 and 23).

For the minimal model without the MinE switch, we used the parameters determined in ref. ^24^ and evaluated the hydrolysis rate *k*_de_ at 37 °C. We scaled the nonlinear reaction rates *k*_dD_ and *k*_dE_ by a factor of 1/60 to rescale the protein concentrations by a factor of 60 and move the region of instability to concentration values comparable with the experimental phase diagram (Fig. 3a).

### Numerical simulation

The spherocylindrical simulation domain was cylindrically symmetric. Moreover, the observed patterns showed the same symmetry after an initial transient period. Employing this symmetry, we simulated the reaction–diffusion dynamics in a two-dimensional radial slice of the spherocylinder for *T* = 3,000 s using COMSOL Multiphysics 6.1 (Supplementary Information Section 9). The simulations used a finite-element discretization on a triangular Delaunay mesh with linear Lagrange elements for the cytosol (bulk) and quadratic elements on the membrane (boundary). Initially, the total protein mass was homogeneously distributed in the cytosolic states *c*_DT_ and *c*_E_ or *c*_E,l_ with weak random perturbations (uniformly distributed within ±1% around the homogeneous concentration). The reduced one-dimensional dynamics (Supplementary Information Section 9) was solved using a uniform finite-differences discretization implemented in Mathematica 13.1 using second-order central differences. Again, the dynamics was simulated for *T* = 3,000 s. In all simulations, we employed the freedom in the choice of units, and the fast-growth wild-type concentrations were normalized to average MinD and MinE concentrations ${\bar{\rho }}_\mathrm{D}=\text{1,000}\,\upmu{}\mathrm{m}^{-3}$ and $\bar{\rho}_\mathrm{E}=\text{1,000}\,\upmu{}\mathrm{m}^{-3}$ by scaling the nonlinear rates *k*_dD_, *k*_dE_, *k*_dE,r_ and *k*_dE,l_ by a factor of 2. Mathematica 13.1 was also used for the linear stability analysis and to analyse the numerical simulations.

### Statistics and reproducibility

To reduce subjectivity, three of the authors classified the experimental data into standing and travelling waves. Phase diagrams were mostly invariant under this operation. In the simulations, we classified and quantified the patterns using the algorithm described in ‘Dynamic pattern classification’ and Supplementary Information Section 11.1. No statistical method was used to predetermine the sample size. No data were excluded from the analyses. The experiments were not randomized. The investigators were not blinded to allocation during the experiments or the outcome assessment.

### Reporting summary

Further information on research design is available in the Nature Portfolio Reporting Summary linked to this article.

## Online content

Any methods, additional references, Nature Portfolio reporting summaries, source data, extended data, supplementary information, acknowledgements, peer review information; details of author contributions and competing interests; and statements of data and code availability are available at 10.1038/s41567-025-02878-w.

## Supplementary information

Supplementary information Supplementary Information Sections 1–12, Figs. 1–26 and Tables 1–3.

Reporting Summary

Supplementary Video 1 Example of an elongated cell with a standing wave.

Supplementary Video 2 Example of an elongated cell with a travelling wave.

Supplementary Video 3 Example of a single cell with both a travelling wave and a standing wave.

Supplementary Video 4 Example of a mini-cell.

Supplementary Video 5 Example of a weak pattern.

Supplementary Video 6 Example of a cell exhibiting typical Min oscillations.

Supplementary Video 7 Example of a cell switching from no pattern to a pattern.

Supplementary Video 8 Example of a cell with patches.

## Source data

Source Data Figs. 2e and 3c Statistical source data for Figs. 2e and 3c and cell kymographs with annotations for a comparison with the theoretical results.

## Figures and Tables

**Extended Data Table 1:**
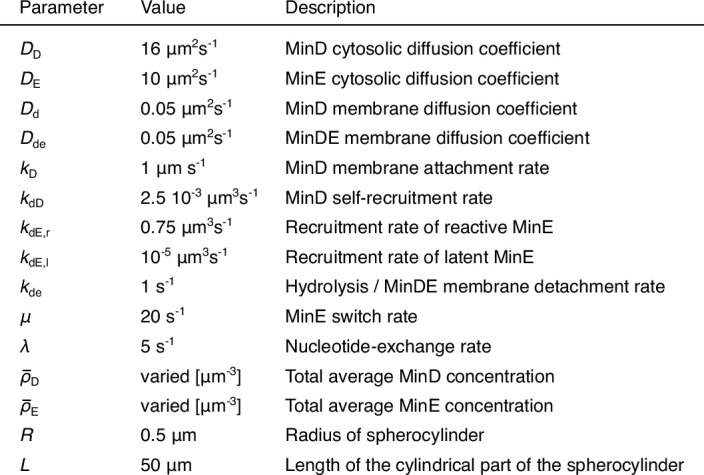
Parameters of the switch model employed in the simulations in Fig. 3

## Data Availability

Owing to the large size (>100 GB) of the underlying data, the full dataset is available upon request. Source data are provided with this paper.

## Code availability

Representative experimental analysis code is available at https://github.com/junlabucsd/min-in-vivo. Representative COMSOL set-up files for the theoretical simulation and the Mathematica notebooks are available at https://github.com/henrikweyer/Min-in-vivo and from ref. ^66^.
