# Supplementary material for: Robust and resource-optimal dynamic pattern formation of Min proteins in vivo
Source: Nat Phys. 2025 May 5;21(7):1160–9. doi: 10.1038/s41567-025-02878-w (PMC12263437; doi:10.1038/s41567-025-02878-w)
Supplement: Supplementary file 1 — Supplementary Information Sections 1–12, Figs. 1–26 and Tables 1–3. [file 41567_2025_2878_MOESM1_ESM.pdf]

nature physics

Supplementary information https://doi.org/10.1038/s41567-025-02878-w

Robust and resource-optimal dynamic pattern formation of Min proteins in vivo

In the format provided by the authors and unedited

Supplemental Information

for "Robust and resource-optimal dynamic pattern formation of Min proteins *in vivo*"

Ziyuan Ren*, Henrik Weyer*, Michael Sandler*, Laeschkir Würthner*,
Haochen Fu, Chanin B. Tangtartharakul,
Dongyang Li, Cindy Sou, Daniel Villarreal,
Judy E. Kim, Erwin Frey**, and Suckjoon Jun**

March 12, 2025

Contents

1 Control of the MinD and MinE levels and the dose-induction curves 2

2 Using mCherry fluorescence as a quantitative proxy of MinE level 3

3 Estimating the MinD and MinE protein concentrations from the fluorescence levels 4
3.1 Estimating concentrations by normalizing by the wildtype . . . 4
3.2 Estimating concentrations using maturation correction . . . 4
3.2.1 Measuring msfGFP and mCherry Maturation Times . . . 4
3.2.2 Estimating Min Protein Concentration Using Wild-Type Fluorescence and Proteomics Data 6

4 Assessing Potential for Off-target effects in tCRISPRi strains 8

5 Strain construction information 10

6 Oscillation period is independent of cell length and growth rate 12

7 Comparison between experimental and theoretical pattern-forming regimes 12

8 Models for the *E. coli* Min system in cellular geometry 12
8.1 The skeleton model . . . 14
8.1.1 Parameter choice . . . 15
8.2 The switch model . . . 15
8.2.1 Parameter choice . . . 16

9 Dimensional reduction of the cellular geometry 16
9.1 Rotational symmetry . . . 17
9.2 Reduction to a one-dimensional model . . . 17
9.3 Numerical simulation . . . 19

10 Linear stability analysis of the homogeneous steady state and the onset of pattern formation 19
10.1 Numerical calculation . . . 20
10.2 Emergence of robust pattern formation due to the MinE switch . . . 20
10.3 Onset of instability in the concentration phase diagram . . . 22
10.4 Quantification of the instability . . . 22

11 Parameter study of the switch model reveals standing- and traveling-wave patterns 24
11.1 Pattern classification . . . 24
11.1.1 Pattern classification in the 1+2D model . . . 26
11.2 Parameter study . . . 26
11.2.1 Wavelength and period of the nonlinear patterns . . . 27

12 The concentration phase diagram for wild-type-length cells 31

1 Control of the MinD and MinE levels and the dose-induction curves

To explore a wide range of MinD and MinE protein levels, we constructed several *E. coli* strains using different inducible promoters. To characterize these promoters, we constructed the dose-induction curves by measuring the fluorescence intensity of the msfGFP translational reporter for MinD (msfGFP-MinD) and the transcriptional reporter mCherry for MinE (MinE mCherry) under different induction levels (Figure S1). We normalized the fluorescence intensity such that the wild-type protein level is 1, which we measured independently using strain SJ1695.

We also characterized gradient repression of *minCDE* by our tCRISPRi strain [1]. In this system, we control the degree of target gene repression by expressing dCas9 using the inducible P_{BAD} promoter at varying arabinose (inducer) concentrations. Shown in Figure S1 (2nd row) is the fold changes of msfGFP-MinD and MinE mCherry vs. inducer concentrations. These results show that we were able to reduce up to 80% of the wild-type protein level (i.e., from 100% to 20%).

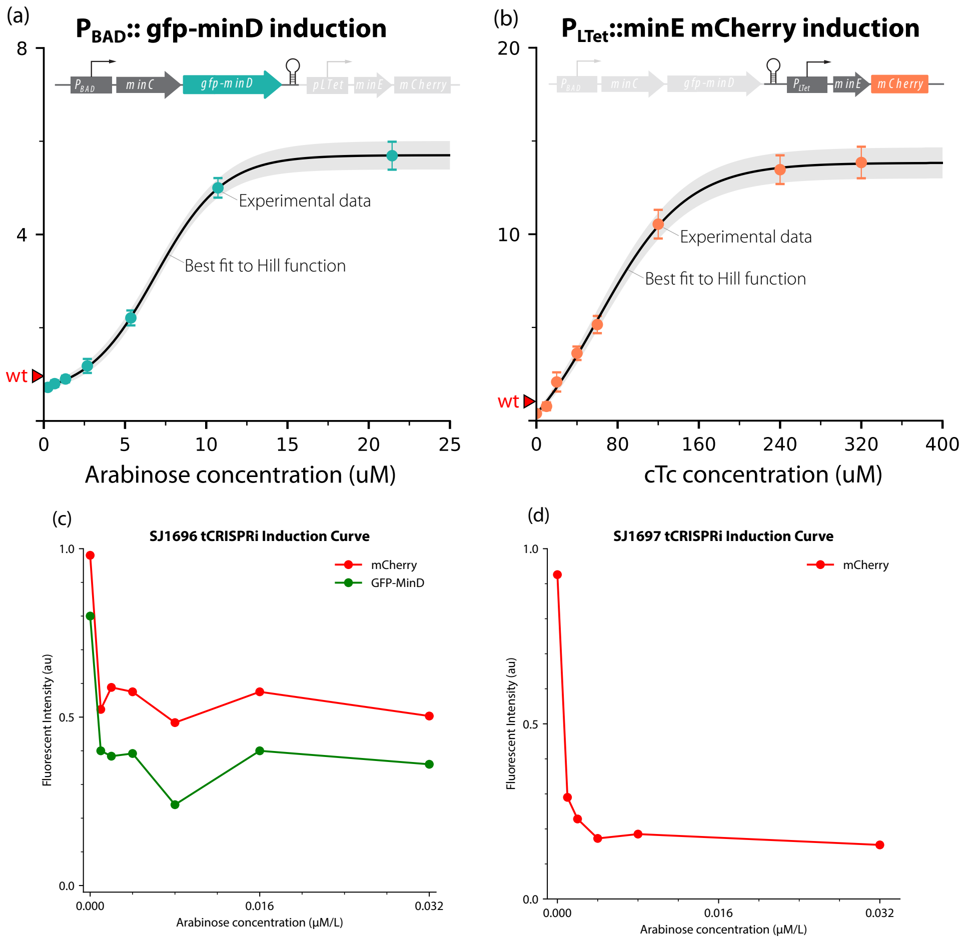

Figure S1. (a) Dose-induction curve of P_{BAD}*::msfGFP-minD. In vivo* msfGFP-MinD expression level increases from below the wild-type level to roughly 6-fold of the wild-type level as the arabinose concentration increases. Data are agar pad means ± SEM. (b) Dose-induction curve of P_{LTet}*::minE mCherry. In vivo* MinE mCherry expression level increases from below the wild-type level to roughly 13-fold of the wild-type level as the cTc concentration increases. Error bars represent the standard deviation of the fluorescence, sampled over a single agar pad's cell population. (c) Dose-induction curve of SJ1696 (*minC* sgRNA). Since this is upstream of *msfGFP-minD* and *minE mCherry*, both fluorescences decrease. (d) Dose-induction curve of SJ1697 (*minE* sgRNA).

2 Using mCherry fluorescence as a quantitative proxy of MinE level

In this work, we used the MinE transcriptional reporter mCherry as a quantitative proxy of MinE proteins within a constant conversion factor, because the MinE function is disrupted when it is translationally fused to fluorescent proteins [2].

The strain SJ2707 was grown in 1L of media with five different inducer concentrations (P_{LTet} 0 ng/ml , 25 ng/ml, 50 ng/ml , 100 ng/ml , 200 ng/ml); a control strain SJ1695 that does not express his$_6$-*minE* his$_6$-*mCherry* was also grown. These growths were performed at 37 C, 200 rpm until samples reached a target OD_{600} of ~0.3. The samples were pelleted into 6 separate pellets and stored overnight in -78 C.

Each pellet was resuspended in 28 ml of buffer A (50 mM sodium phosphate pH 8.0, 500 mM NaCl, and 10 mM imidazole). 2 mL of a stock solution of DNase in 10x reaction buffer (10 mM tris pH 7.5, 25 mM $MgCl_2$, and 5 mM $CaCl_2$) was added to a final concentration of 170 U/mL. To this 30 ml solution, EDTA-free protease inhibitor, lysozyme (final concentration 67 µg/ml), and 2-mercaptoethanol (final concentration 10 mM) were added. This lysate was then probe sonicated for 5 minutes at 50 % duty cycle, half second pulses. The lysate containing crude protein was spun and the supernatant was collected for purification.

A Ni-NTA column was used for purification of his$_6$-MinE and his$_6$-mCherry on an FPLC. Mixtures of buffers B and C were used to vary the imidazole concentration for elution of the his-tag proteins. Both buffers contained 50 mM sodium phosphate pH 8.0, 500 mM NaCl, and 10 mM 2-mercaptoethanol. Buffers B and C additionally contained 20 mM or 500 mM imidazole, respectively. The bound proteins were separated by imidazole concentrations of 70 mM, 260 mM, and 500 mM imidazole. Both his$_6$-MinE and his$_6$-mCherry eluted at 260 mM imidazole. The tubes containing his$_6$-MinE and his6-mCherry were collected and concentrated with 3K centrifugal concentrators, resulting in a relatively pure sample of his$_6$-MinE and his$_6$-mCherry corresponding to each of the five inducer concentrations. An identical procedure was followed for the control though no his$_6$-MinE and his$_6$-mCherry was detectable. The samples were buffer exchanged into a storage buffer containing 50 mM HEPES, 0.1 mM EDTA, and 150 mM KCl (pH 7.2).

The proteins were analyzed with UV-vis absorption and fluorescence spectroscopy as well as SDS PAGE gel electrophoresis. The absorption spectra revealed relatively constant ratios of the absorbance at 587 to 280 nm, where 587 nm reflects only his$_6$-mCherry while 280 nm reflects his6-MinE, his6-mCherry (see Figure S2). The ratios were 1.10, 1.07, 1.08, and 1.07 for 25, 50, 100, and 200 ng/ml, respectively (these values were determined after subtraction of the absorption spectrum of 0 ng/ml inducer). The density of the gel bands were analyzed using GelAnalyzer, and the ratio [his$_6$-mCherry]/[his$_6$-MinE] was 2.1, 1.7, 1.8, 1.4 for 25, 50, 100, and 200 ng/ml, respectively; these ratios took into account the different molecular weights of the proteins.

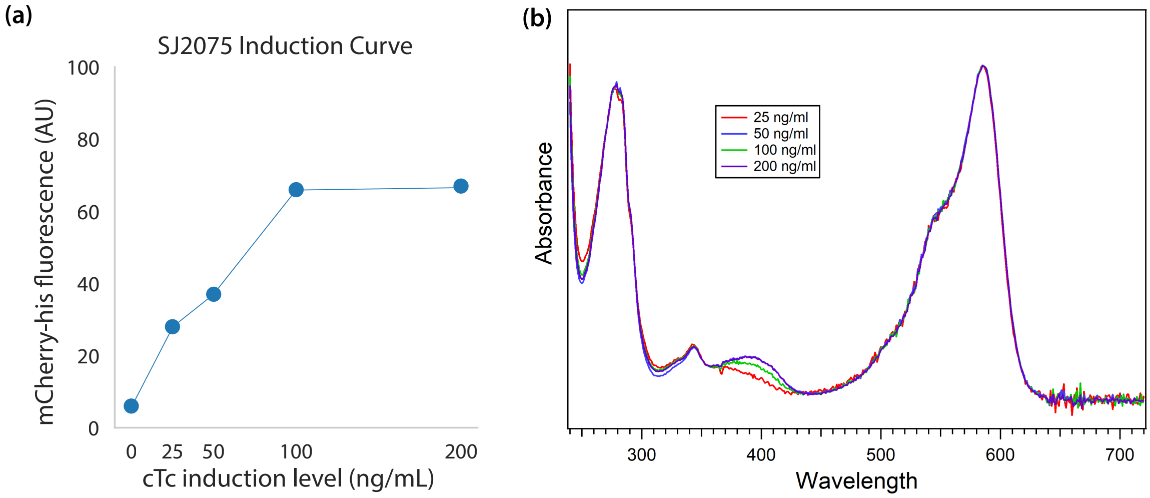

Figure S2. (a) Induction curve of SJ2075. (b) Absorption spectra of purified sample at 4 P_{LTet} inducer concentrations. The spectrum of 0 ng/ml inducer concentration has been subtracted. The spectra were normalized at 587 nm.

3 Estimating the MinD and MinE protein concentrations from the fluorescence levels

3.1 Estimating concentrations by normalizing by the wildtype

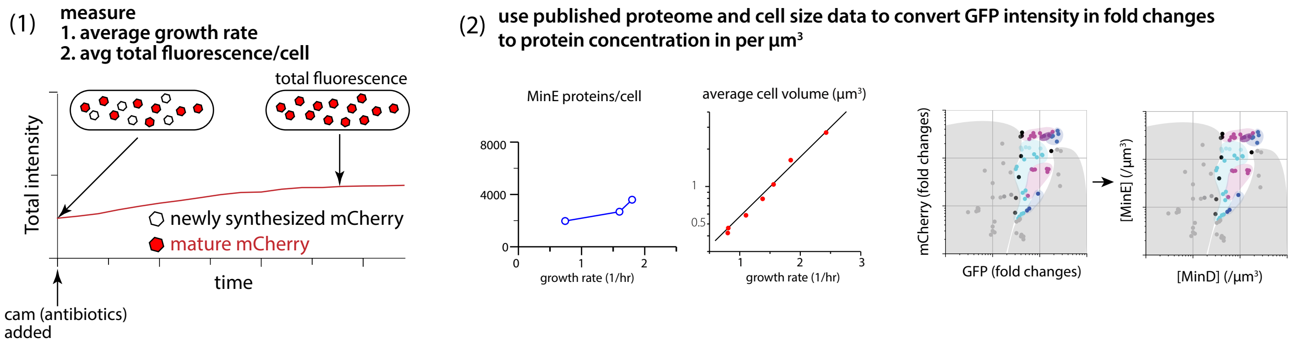

Figure S3. Fluorescent intensity is converted into protein number by 1) correcting for protein maturation [see: Maturation correction] and 2) converting the fluorescent intensity into absolute protein numbers using published numbers [see: Estimating concentrations]

In Section 2, we demonstrated that the transcriptional reporter mCherry can act as a quantitative indicator of MinE levels. Here, we describe the procedure for determining the conversion factor between mCherry and msfGFP fluorescence and MinE/MinD protein concentration respectively.

We performed this conversion in two steps (Figure S3):

1. Fluorescence Measurement: We measured the average total fluorescence intensity, I , of msfGFP and mCherry in vivo by integrating fluorescence across the segmented cell area and normalizing by the calculated cell volume. To quantify the fold changes in fluorescence intensity—and thus in MinD or MinE concentrations—under different induction levels, we normalized I by the wild-type intensity I_{wt}, obtaining $I_{\mathrm{fold}} = I/I_{\mathrm{wt}}$.

2. Conversion to Protein Concentration: To translate I_{fold} into protein concentrations, we referenced published proteomics and RiboSeq data (Fig. 2d) alongside cell size versus growth rate data [3]. Under fast-growth conditions (25-minute doubling time), the conversion factor was 2000 MinD (and MinE) proteins per μm^3. For slower growth conditions (doubling time of 55 minutes), the concentration was approximately 1.5 times higher, at 3000 proteins/μm^3. Using this conversion factor, we estimated average MinD and MinE protein concentrations based on fluorescence measurements of msfGFP or mCherry. This approach allows for direct comparison to theoretical predictions.

3.2 Estimating concentrations using maturation correction

To validate the trends observed in the proteomics dataset, we also wanted to quantify (wildtype) MinE and MinD concentrations without relying on any external sources. To do this, we measured the maturation times of msfGFP and mCherry, which then allowed us to calculate the fold-change of MinD and MinE respectively. Indeed, we find that both our own data and several external sources all display the same trends in MinD and MinE copy numbers. We provide detail for this in sections 3.2.1 and 3.2.2.

3.2.1 Measuring msfGFP and mCherry Maturation Times

To measure the maturation times of the fluorescent proteins, we grew our wild-type msfGFP-MinD and MinE-mCherry strain (SJ1695) to exponential phase. We then added chloramphenicol to halt protein synthesis and monitored the fluorescence per cell over 45 minutes. Fluorescence time traces are shown in Figure S4, with $t = 0\,\mathrm{min}$ marking the cessation of protein synthesis.

The total msfGFP-MinD fluorescence signal remained nearly constant, indicating that msfGFP matures rapidly. By contrast, the mCherry signal increased before plateauing, suggesting that mCherry proteins synthesized prior to the stop required tens of minutes to fully mature and fluoresce. The fraction of non-mature fluorescent proteins should increase proportionally with the ratio of the maturation time to the doubling time. Indeed, our experiments (Figure S4) indeed show that, after full maturation, the total mCherry fluorescence per cell increased by a factor of 1.3 (for a doubling time of 55 minutes) and 1.7 (for a doubling time of 25 minutes).

By correcting the total mCherry fluorescence per cell using these maturation factors, we were able to compare fluorescence levels across different growth conditions. After adjusting for mCherry maturation time, we calculated the MinD/MinE ratio (Figure S5) and found it to be nearly constant across all four growth conditions, consistent with the proteomics data.

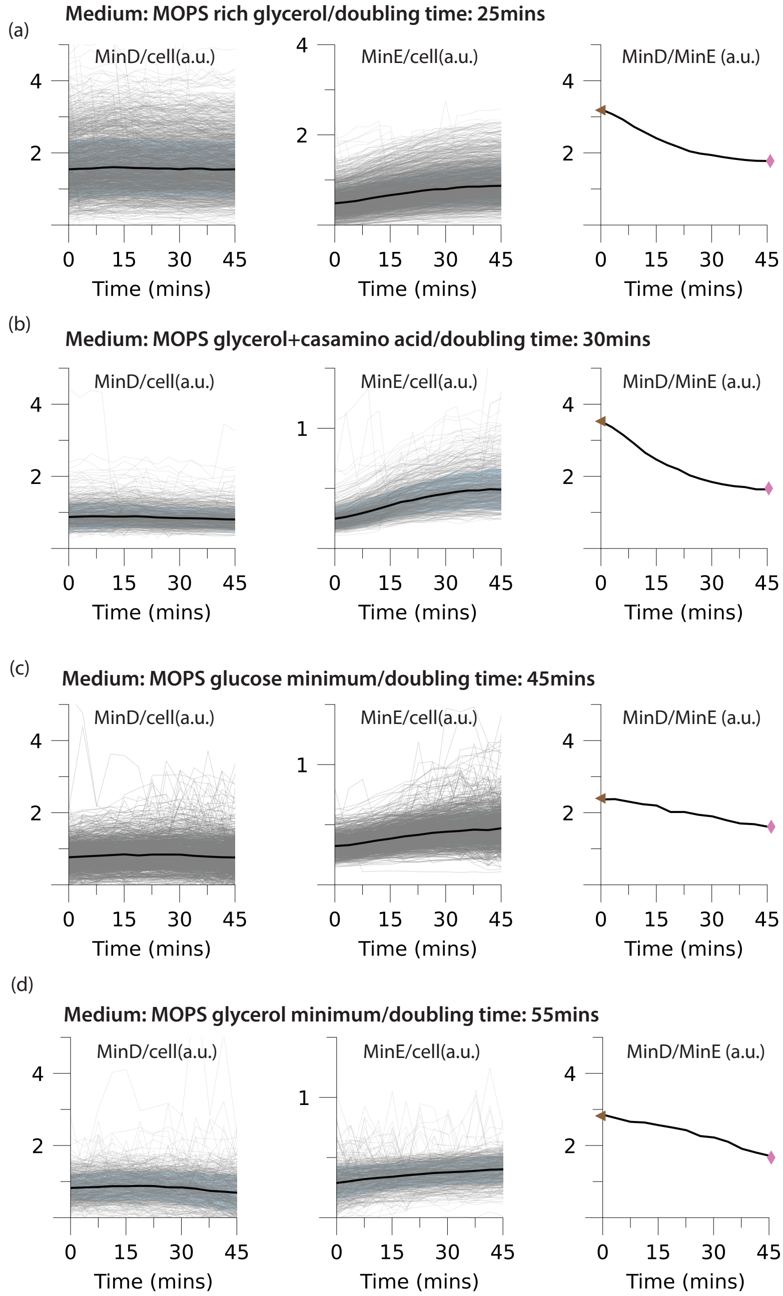

Figure S4. Maturation curves for msfGFP-MinD and MinE mCherry (wild-type strain SJ1695) at different growth conditions. Cells were treated with chloramphenicol 2 mins prior to t=0 mins to stop protein synthesis. The panels show the total fluorescence per cell of msfGFP (left) and mCherry (middle). The average over the different cells (gray) is shown in black. The time evolution of the ratio of the average msfGFP and mCherry fluorescence levels are shown on the right. The different panels show the fluorescence time traces in (a) MOPS Rich Glycerol, (b) MOPS glycerol+casamino acid, (c) MOPS glucose minimal, and (d) MOPS glycerol minimal growth medium.

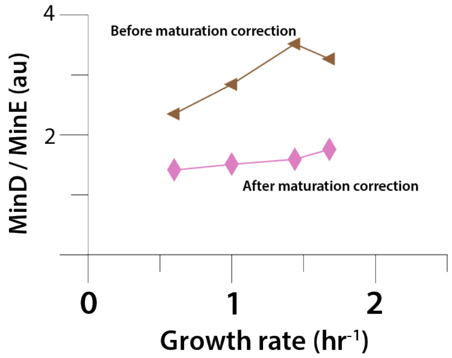

Figure S5. MinD/MinE intensity ratio is roughly constant after maturation correction.

3.2.2 Estimating Min Protein Concentration Using Wild-Type Fluorescence and Proteomics Data

We considered three proteomics datasets for this conversion: one obtained via ribosome sequencing (RiboSeq) [4], another using mass spectrometry [5], and a third combining both methods [6]. For completeness, we provide the growth-rate-dependent copy numbers of MinC, MinD, and MinE per cell in Figure S7a. By inferring cell size from growth rate using our previously published data [3], we then calculated the corresponding protein concentrations. Despite variations in absolute values across datasets, the growth-rate-dependent trends remain consistent. Additionally, the ratio of MinD to MinE remains fairly constant across all datasets, in agreement with our own fluorescence measurements.

Notably, all published data show consistent trends in protein copy numbers per cell and protein concentrations as a function of growth rate (Figure S7). Specifically, the ratio of MinE to MinD remains close to 1 (Figure S6). Using the protein copy numbers from the RiboSeq dataset and the cell-size data [3], we calculated the average concentrations of MinD and MinE for the four growth conditions we tested.

To convert the fluorescent intensity into protein copy numbers, we utilized published proteomics and cell-size data to derive a conversion factor between protein copy number and fluorescence per unit volume. We used protein concentrations from the RiboSeq dataset [4]. This dataset was selected because it employed the same *E. coli* MG1655 strain background as in our study, covered a similar range of growth rates, and provided a reliable estimate for the relatively small protein copy numbers characteristic of Min proteins.

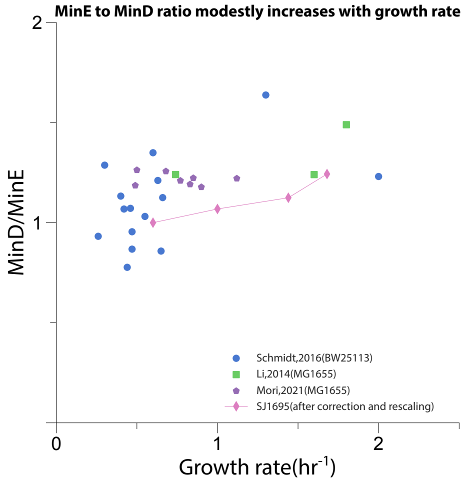

Figure S6. After correcting for the expected relative intensities of MinD and MinE, fluorescent intensity for MinE and MinD has the same arbitrary units. Our measured data replicates trends observed in different data sets.

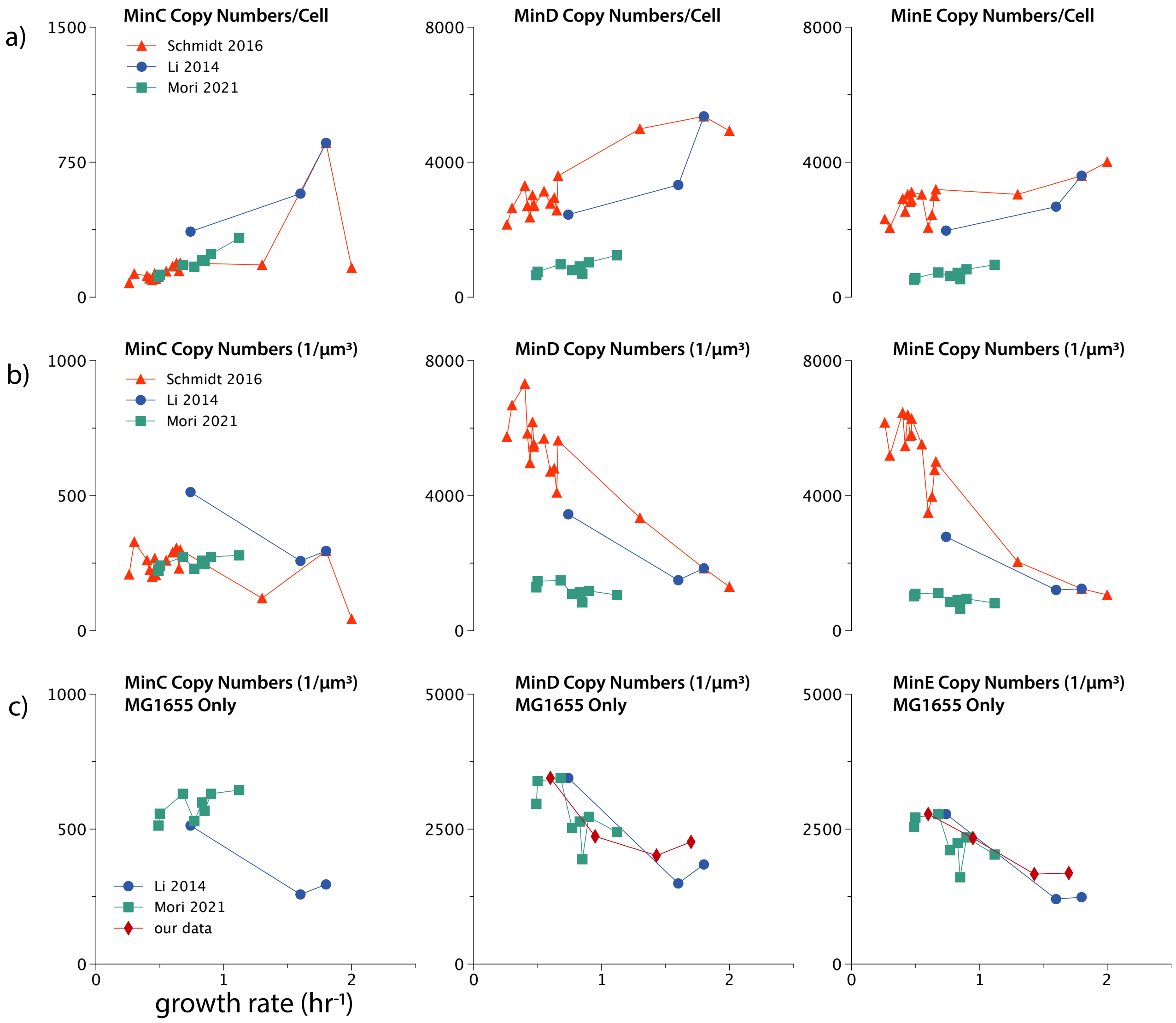

Figure S7. Min protein copy number per cell and micrometer. (a) MinC, MinD and MinE protein copy number per cell under different growth rates. (b) Raw MinC, MinD and MinE protein copy number per cubic micrometer; volume from [3]. (b) Normalized MG1655 MinC, MinD and MinE protein copy number per cubic micrometer, with our fluorescence data overlaid. A downward slope is clearly visible across datasets.

4 Assessing Potential for Off-target effects in tCRISPRi strains

In addition to the dual-inducible strain, we also used a modified version of our tunable CRISPRi (tCRISPRi) strain that we developed together with Don Court's lab at the National Cancer Institute [1]. In this strain, *dCas9* is expressed under the inducible P_{BAD} promoter such that varying concentrations of inducer lead to variable repression of genes targeted by a guide RNA (*sgRNA*). By inserting the appropriate guide RNA (*minC* in SJ1696 and *minE* in SJ1697), we analyzed Min pattern formation under gradient repression of *minCDE* and *minE* in SJ1696 and SJ1697 respectively.

The specificity of tCRISPRi [1] had previously been demonstrated. Specifically, for a 20 base long target sgRNA, we found that even 2-3 bp mismatches resulted in a two-fold reduction in repression efficacy, and 5bp mismatches resulted in a complete loss of repression. In other words, the system is generally robust to off-target effects.

To verify this specifically for our SJ1696 and SJ1697 strains, we performed RNA sequencing for three different inducer concentrations, with two replicates for each induction level.

For SJ1696 (*minC sgRNA*), in which the whole *minCDE* locus is repressed by the sgRNA binding at its beginning (at *minC*), we observed no significant off-target effects for zero to medium induction levels. At high induction levels, an around 8-fold increase in *cysU* and *cysD* expression is observed. However, this is well into the zone of no-pattern formation, and thus does not affect our results (refer to the data table, as well as figure S1).

For SJ1697 (*minE sgRNA*), we observed some off-target effects. However, off-target affected genes are all part of the flagellar system, which recent work has shown is directly regulated by the Min system [7]. Our data supports this observation. Because we do not study the motility of the bacteria, those changes in the flagellar system do not change the conclusions of our work. Similar to the other strain, a significant degree of repression of non-flagellar genes only occured at the highest expression level in Fig. S8. Again, this repression was no more than $2^{2.5}$-fold, less than 1% of the (512-fold) repression of the targeted *minE* itself, and occured well into the no-pattern regime.

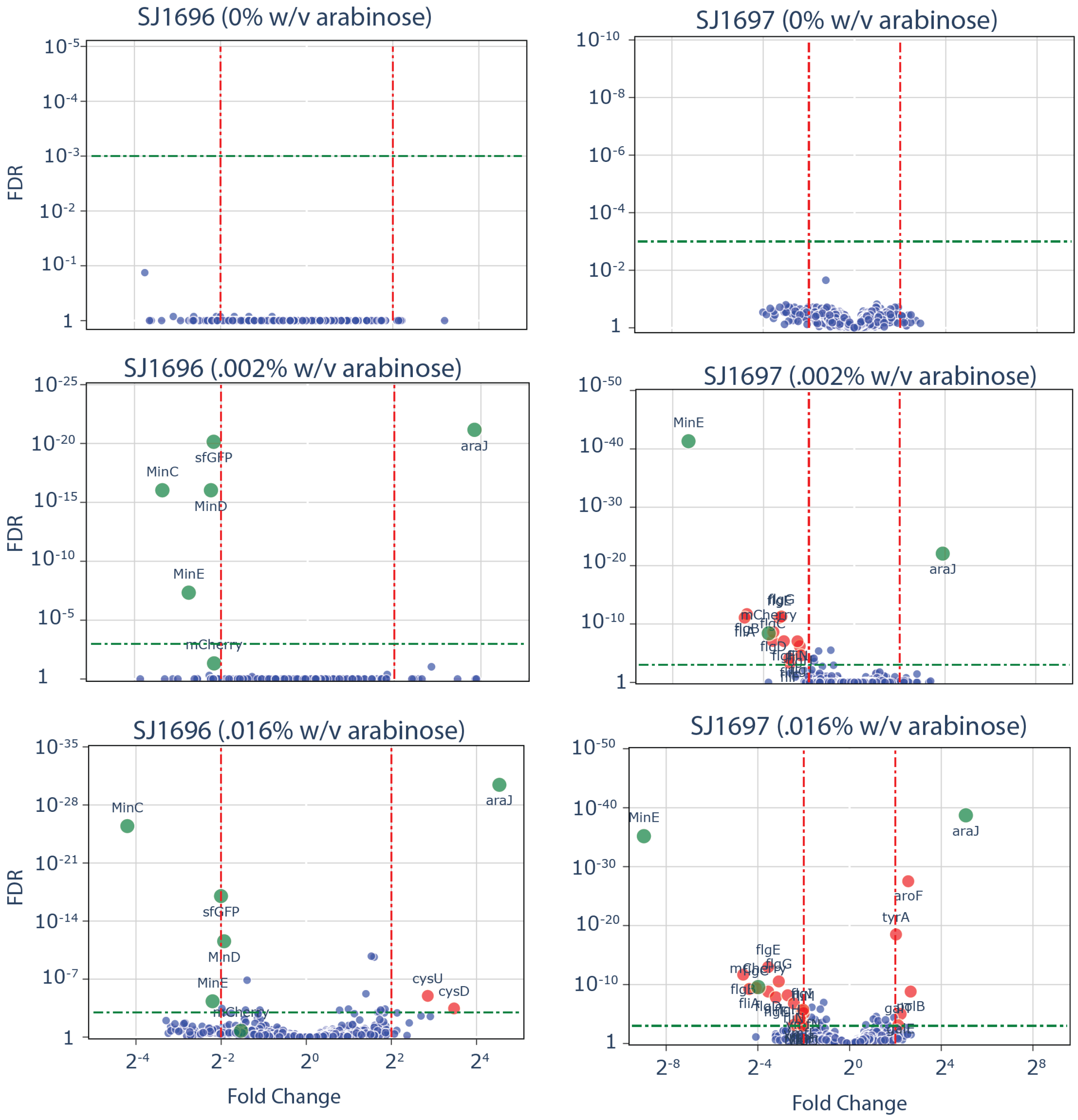

Figure S8. RNA sequencing results of tCRISPRi strains. (a) Volcano plots at different arabinose induction levels are shown for the strand SJ1696. The targeted genes (minCDE, araJ, and msfGFP) are highlighted in green. Off-target genes that meet a standard criteria of an at least 4-fold change in expression level and an FDR of at least .001 are highlighted in red. (b) Volcano plots at different arabinose induction levels are shown for the strain SJ1697.

5 Strain construction information

We generated the strain SJ1883 used in this study by genome engineering endogenous minCDE locus. We first generated msfGFP-minD fusion following the same fusion strategy used in Wu et al., 2015 (strain FW1541 in [8]) based on our tCRISPRi strain in [1]. Then we added a transcriptional reporter for minE by inserting a Shine-Dalgarno sequence followed by mCherry coding sequence downstream of the endogenous minE locus. The strain was then cured of sgRNA and dCas9 insertion loci irrelevant to the current study. This results in strain SJ1878 with fluorescent reporters for minD and minE respectively (minC msfGFP-minD minE mCherry).

To introduce tunable control, a cassette containing a terminator and P_{LTet} promoter was inserted between minD and minE, followed by insertion of P_{BAD} promoter upstream of minC. A copy of tetR was introduced to yfdG locus to regulate P_{LTet} expression and the P_{BAD} is regulated through the modified P_{BAD}* in Li et al., 2016. To generate the single inducible strain, P_{BAD} promoter was replaced with endogenous minCDE promoter in SJ1880 (P_{BAD}::minC msfGFP-minD KmR; P_{LTet}::minE mCherry; Δ araBAD; ΔsgRNA) , and tet-sacB was inserted in yfdG locus, eventually replaced by tetR to generate SJ1935 (minC msfGFP-minD KmR; P_{LTet}::minE mCherry; Δ araBAD; Δ sgRNA; P_{LTet}::tetR<>yfdG).

In order to ensure that we had the same copy numbers of MinE and mCherry, we tried adding his- tags to both so that they could be purified and quantified (see section 2). Unfortunately, we found that cells where the only copy of MinE was his-tagged were very unhealthy, and we were not able to get such a strain to work. To construct strain SJ2707, we placed a separate copy of his-MinE mCherry-his onto the P_{LTet} promoter. A simplified description of strains is in 1, and a more detailed list of all strains used for the sake of reproducibility is available in S9.

**Strain**	**Genotype**
SJ1695	P_{BAD}_dCas9 ΔLacI ΔaraE araFGH<>spec lacYA177C; galM// tet-sacB//gmpA; minC msfGFP-minD minE SD mCherry; pSIM18
SJ1696	P_{BAD}_dCas9 ΔLacI ΔaraE araFGH<>spec lacYA177C; galM// minC sgRNA//gmpA; minC msfGFP-minD minE SD mCherry; pSIM18
SJ1697	P_{BAD}_dCas9 ΔLacI ΔaraE araFGH<>spec lacYA177C; galM// minE sgRNA//gmpA; minC ms-fGFP minD minE SD mCherry pSIM18
SJ1883	P_{BAD}::minC msfGFP-minD KmR P_{LTet}::minE mCherry; araC cat ΔaraBAD; ΔsgRNA; yfdG P_{LTet}::tetR
SJ1935	minC msfGFP-minD KmR; P_{LTet}::minE mCherry; P_{LTet}::tetR<>yfdG ΔaraBAD; ΔsgRNA;

Table 1. Strain information and genotypes

SJ strain #	alternate ID	relevant genotype	procedure	parental strain
546	BEC-56, FW1541	ecosfGFP-minD FRT-aph-FRT		FW1541, from Wu Nature Nanotec h 2015
797	SJ_XTL219	PBAD_dCas9 ΔLacI ΔaraE araFGH<>spec lacYA177C galM//promoter-tet-sacB -handle-terminator//gmpA pSIM18		
1287	CS007	sfGFP-minD pSIM18	Transformation of pSIM18 to SJ546, selected on hygro plates	SJ546
1288	CS008	cm-sfGFP-minD pSIM18	Recombination of cmR into CS007 (primers CS003 and CS004), selected on cm plates	CS007
1289	CS009	PBAD_dCas9 ΔLacI ΔaraE araFGH<>spec lacYA177C galM minC-cm-sfGFP-minD pSIM18	P1 transduction of translational reporter and GFP-minD linker cm-sfGFP to tCRISPRi strain (SJ_XTL219), selected on cm plates and confirmed by PCR (primers CS022 and CS023) and sequencing	SJ_XTL21 9
1291	CS011	PBAD_dCas9 ΔLacI ΔaraE araFGH<>spec lacYA177C galM// minE sgRNA//gmpA minC-cm-sfGFP-minD pSIM18	Recombination of minE sgRNA (oligo DL87) to CS009, selected on sucrose plate and PCR confirmed with primers SJ_XT23 and SJ_XT139	CS009
1293	CS013	PBAD_dCas9 ΔLacI ΔaraE araFGH<>spec lacYA177C galM// minE sgRNA//gmpA minC-cm-sfGFP-minD-minE-tet-sacB pSIM18	Recombination of tet-sacB to be replaced by flourescent reporter for transcriptional reporter. Recombination of tet-sacB (PCR product from template SJ_XTL219 and primers DL88 and CS006) behind minE, selected on tet plate behind minE	CS011
1296	CS016	PBAD_dCas9 ΔLacI ΔaraE araFGH<>spec lacYA177C galM// minE sgRNA//gmpA minC-cm-sfGFP-minD-minE-mCherry pSIM18	Recombination of mCherry transcriptional reporter. Recombination of mCherry (PCR product from template SJ_XTL344 and primers CS007 and CS008) replacing tet-sacB behind minE, selected on sucrose plate and confirmed by negative selection on tet plate	CS013
1682	CS022	PBAD_dCas9 ΔLacI ΔaraE araFGH<>spec lacYA177C galM// minE sgRNA//gmpA minC-tet-sacB-minD-minE-mCherry pSIM18	Recombination of tet-sacB to CS014. Primers CS034 and CS035 from SJXTL99 liquid culture.	CS016
1686	CS026	PBAD_dCas9 ΔLacI ΔaraE araFGH<>spec lacYA177C galM// minE sgRNA//gmpA minC-GFP-minD-minE-mCherry pSIM18	Recombination of GFP-linker-minD to replace tet-sacB. Primers CS036 and CS037, template SJ546.	CS022
1690	CS030	PBAD_dCas9 ΔLacI ΔaraE araFGH<>spec lacYA177C galM// minE sgRNA//gmpA minC-GFP-minD-minE-tet-sacB-mCherry pSIM18	Recombination of tet-sacB behind minE to insert SD sequence. Primers CS031 and CS032, template tet-sacB PCR product.	CS026
1692	CS032	PBAD_dCas9 ΔLacI ΔaraE araFGH<>spec lacYA177C galM// minE sgRNA//gmpA minC-GFP-minD-minE-SD-mCherry pSIM18	Recombination of SD sequence (CS029) to replace tet-sacB behind minE	CS028
1693	CS033	PBAD_dCas9 ΔLacI ΔaraE araFGH<>spec lacYA177C galM// tet-sacB//gmpA minC-GFP-minD-minE-SD-mCherry pSIM18	Recombination to replace minC sgRNA with tet-sacB	CS032
1694	CS034	PBAD_dCas9 ΔLacI ΔaraE araFGH<>spec lacYA177C galM// minE sgRNA//gmpA minC-GFP-minD-minE-SD-mCherry pSIM18	Recombination of SD sequence (CS029) to replace tet-sacB behind minE	CS030
1695	CS035	PBAD_dCas9 ΔLacI ΔaraE araFGH<>spec lacYA177C galM// tet-sacB//gmpA minC-GFP-minD-minE-SD-mCherry pSIM18	Recombination to replace minE sgRNA with tet-sacB	CS034
1694	CS034	PBAD_dCas9 ΔLacI ΔaraE araFGH<>spec lacYA177C galM// minE sgRNA//gmpA minC-GFP-minD-minE-SD-mCherry pSIM18	Recombination of SD sequence (CS029) to replace tet-sacB behind minE	CS030
1696	CS036	PBAD_dCas9 ΔLacI ΔaraE araFGH<>spec lacYA177C galM// minC sgRNA//gmpA minC-GFP-minD-minE-SD-mCherry pSIM18	Recombination of updated minC sgRNA (CS043) to replace tet-sacB	CS033
1697	CS037	PBAD_dCas9 ΔLacI ΔaraE araFGH<>spec lacYA177C galM// minE sgRNA//gmpA minC-GFP-minD-minE-SD-mCherry pSIM18	Recombination of updated minE sgRNA (CS044) to replace tet-sacB	CS035
1695	CS035	PBAD_dCas9 ΔLacI ΔaraE araFGH<>spec lacYA177C galM// tet-sacB//gmpA minC-GFP-minD-minE-SD-mCherry pSIM18	Recombination to replace minE sgRNA with tet-sacB	CS034
1862	DL225	minC GFP-minD minE mCherry; PBAD::dCas9;galM-gpmA	Recombineer with SJ_XT_392/SJO1018 and recover wild type galM-gpmA	SJ1695
1878	DL241	minC GFP-minD minE mCherry; ΔaraBAD; galM-gpmA	Recombineering DL225 with PCR product DL198, DL137 (tet-sacB<>araBAD); then recombineering with SJO1515	SJ1862
1877	DL240	minC GFP-minD KmR; PLTet::minE mCherry; ΔaraBAD; ΔsgRNA	Recombineering with PCR product DL308, DL309	SJ1878
1879	DL242	tet-sacB minC GFP-minD KmR; PLTet::minE mCherry; ΔaraBAD; ΔsgRNA	Recombineering with PCR product DL227, DL228	SJ1877
1880	DL243	PBAD::minC GFP-minD KmR; PLTet::minE mCherry; ΔaraBAD; ΔsgRNA	Recombineering with PCR product DL306, 307	SJ1879
1881	DL244	PBAD::minC GFP-minD KmR; PLTet::minE mCherry; ΔaraBAD; ΔsgRNA; yfdG-[tet-sacB]	Recombineering with PCR product DL263, 264	SJ1880
1882	DL245	PBAD::minC GFP-minD KmR; PLTet::minE mCherry; ΔaraBAD; ΔsgRNA; yfdG-PLTet::tetR	Recombineering with PCR product DL265, 266	SJ1881
1883	DL246	PBAD::minC GFP-minD KmR; PLTet::minE mCherry; ΔaraBAD; ΔsgRNA; yfdG-PLTet::tetR; [araC cat ΔaraBAD]	recombineering with PCR product of DL150, XT122 based on a intermediate strain that inserts pCat::cat into a WT strain using recombineering with PCR from DL305, DL321	SJ1882
1934	DL265	minC GFP-minD KmR; PLTet::minE mCherry; ΔaraBAD; ΔsgRNA; tet-sacB<>yfdG, pSIM18	recombineering to recover endogenous minCDE promoter and reintroduce tet-sacB to yfdG locus	SJ1880
1935	DL266	minC GFP-minD KmR; PLTet::minE mCherry; ΔaraBAD; ΔsgRNA; PLTet:: tetR<>yfdG	Recombineering with PCR product DL265, 266	SJ1934
2705	MS005	MG1655 Δ(lacI, lacY, galK, ryhB) KanR rrnBT PLTet::lacZ Δbla::PLTet:tetR, pSIM18		
2064	HF118	dnaA::msfGFP kanR mCherry-dnaN, ΔlacI ΔlacZ::PLTet:his-minE mCherry-his ampR, tetR<>yfdg, pSIM18	Unstable PLTet promoter. Linker from Glock et al 2018 [56]	
2707	MS007	ΔlacI ΔlacZ::PLTet:his-minE mCherry-his ampR, PLTet::tetR<>yfdg	Recombineering from SJ2075. Fragment from SJ2064 using primers MS001 CJ051, then SJO1791 SJO1792	SJ2705

Figure S9. A list of all strains used to create the strains in this project

6 Oscillation period is independent of cell length and growth rate

In the main text, Fig. 3c showed that the wavelength of the Min oscillations is constant across different Min protein expression levels. In contrast, the oscillation period depends on the Min protein expression levels, in particular the MinE concentration.

The oscillation period is independent of both growth condition and the cell length. To this end, Fig. S10a shows the oscillation periods of cells with MinE at a concentration of about 6000 $/\mu m^3$. Panel b shows the oscillation periods of cells with MinE concentration of about 15000 MinE$/\mu m^3$. Moreover, Fig. S10 clearly shows that the oscillation period is independent of both the growth condition and the cell length.

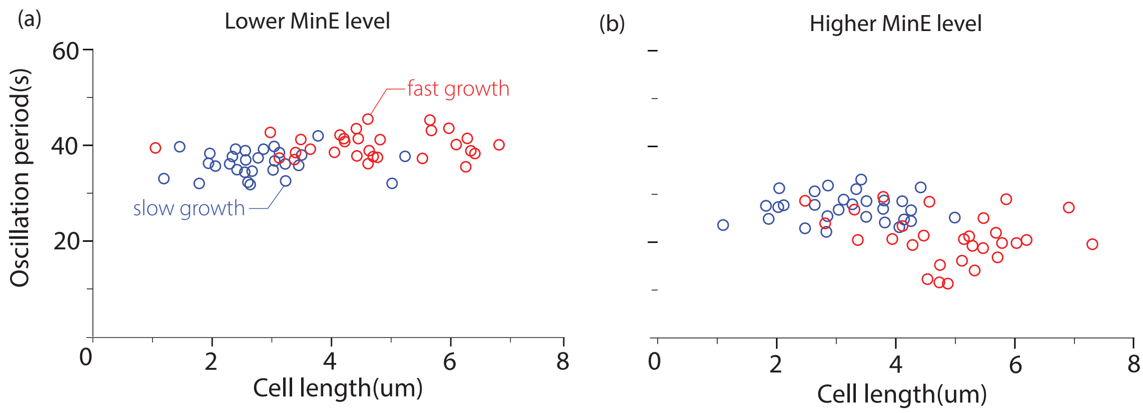

Figure S10. Oscillation period for fast and slow growth conditions at similar Min protein levels. Each data point represents an individual cell. (a) Period measurement of fast- and slow-growing cells with [MinE] $=$ 6000 proteins $/\mu m^3$and wild-type expression levels. (b) Period measurement of fast- and slow-growing cells for [MinE] $=$ 15000 proteins $/\mu m^3$.

7 Comparison between experimental and theoretical pattern-forming regimes

When comparing the experiment and theory phase diagrams, the theoretical pattern-formation regime extends towards higher MinD levels than the experimental one (Fig. 3b in the main text and Fig. S11). Specifically, in the experiment, no dynamic Min patterns are observed at MinD concentrations above about 10 000 μm^{-3}. In contrast, the theoretical model predicts the existence of the dynamic patterns also above this concentration for a wide range of parameters (Fig. S11 and Figs. S15, S21).

We attribute this apparent discrepancy to two effects. First, the experimental points are sparse in this region of the phase diagram. The first measurements showing the lack of dynamic Min patterns lie at much higher MinD concentrations of about 20 000 μm^{-3} and low MinE concentrations of about 3000 μm^{-3}, and one experimental condition at MinD concentration of about 18 000 μm^{-3} and MinE concentrations of about 14 000 μm^{-3} (see Fig. 2e in the main text). At the conditions with lower MinE values, also the simulated phase diagram does not show patterns. Second, we expect that MinD aggregation, which is not considered in the MinE-switch model, reduces the region of pattern formation at high MinD levels in the experiments compared to the model. MinD self-interactions and oligomerization have previously been observed by other groups *in vitro* at high concentrations [9–13]. Within our own data, at high msfGFP-MinD levels, msfGFP-MinD usually forms small bright spots (which we interpret as aggregates) that sometimes move within the cell, or even turn into moving patterns (SI movie 7). At MinD concentrations above the regime of dynamic patterns, the aggregates are often more prominent and barely move (SI movie 8).

8 Models for the *E. coli* Min system in cellular geometry

The basic interaction motives between MinD and MinE have been employed to explain Min protein patterns by a reaction–diffusion mechanism coupling their attachment onto and detachment from the cell membrane to cytosolic and membrane diffusion [14]. Several models that focus on different aspects of the reaction network of the Min proteins have been proposed. The mathematical formulation of the minimal model without the MinE switch ("skeleton model") [15, 16] and with the MinE switch ("switch model") [17] used to explain the experimental

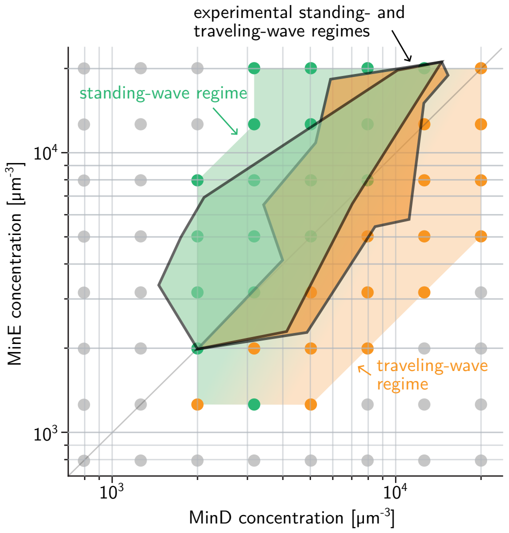

Figure S11. Comparison between the experimental and theoretical phase diagrams. The experimental standing- and traveling-wave regimes (black bordered regions; cf. Fig. 3b in the main text) measured in filamentous cells are overlayed with the simulated phase diagram for filamentous cells (green-orange-shaded region and points; cf. Fig. 3b in the main text).

data obtained in this study is given in Materials and Methods. We restate the reaction–diffusion equations here in a general form which we use below to reduce the dynamics to a one-dimensional description. We also give the expressions for the general terms in the skeleton and switch models.

To simulate the formation of protein patterns in reaction–diffusion models, one describes the different protein states by their spatiotemporal concentration within the cytosol and on the cell membrane [18]. We model the cellular geometry by a spherocylinder consisting of a cylinder of length L and radius R and two spherical caps at both ends with the same radius R [see Fig. S12(a), $2+3$D geometry]. The cell cytosol corresponds to the bulk of the spherocylinder. The cell membrane is modeled as the surface of the spherocylinder.

The reaction–diffusion dynamics of the proteins follows a set of partial differential equations for all the protein concentrations. The protein concentrations $\mathbf{c}(\mathbf{x},t)$ in the cytosol undergo diffusion in the 3D cell interior and fulfill

$$\partial_t \mathbf{c} = \mathbf{D}_\mathrm{c} \nabla^2 \mathbf{c} + \mathbf{r}_\mathrm{c}(\mathbf{c}). \tag{1}$$

The cytosolic diffusion coefficients of the different protein species are contained in the diagonal matrix $\mathbf{D}_\mathrm{c}$. We neglect any inhomogeneities in the cytosol, e.g., due to the nucleoid, and approximate the protein motion by average, spatially uniform diffusion constants. The reaction term $\mathbf{r}_\mathrm{c}(\mathbf{c})$ describes the conversion reactions between the cytosolic species. The membrane concentrations $\mathbf{m}(\mathbf{x},t)$ are similarly determined by

$$\partial_t \mathbf{m} = \mathbf{D}_\mathrm{m} \nabla_\mathrm{m}^2 \mathbf{m} + \mathbf{r}_\mathrm{m}(\mathbf{m}, \mathbf{c}|_\mathrm{m}). \tag{2}$$

Here, the diagonal matrix $\mathbf{D}_\mathrm{m}$ contains the membrane diffusion constants, and ∇_m^2 denotes the Laplace-Beltrami operator necessary to describe the protein diffusion along the curved cell membrane. The reaction term $\mathbf{r}_\mathrm{m}(\mathbf{m}, \mathbf{c}|_\mathrm{m})$ depends both on the membrane concentrations as well as the cytosolic concentrations at the membrane $\mathbf{c}|_\mathrm{m}$ because it describes attachment onto and detachment from the membrane as well as conversion between membrane species.

The attachment and detachment processes induce protein fluxes in the cytosol normal to the cell membrane [see Fig. S12(d)]. This coupling of the cytosolic concentrations to the membrane dynamics is described by the reactive boundary conditions

$$\mathbf{n} \cdot \mathbf{D}_\mathrm{c} \nabla \mathbf{c}|_\mathrm{m} = \boldsymbol{f}(\mathbf{m}, \mathbf{c}|_\mathrm{m}). \tag{3}$$

The vector $\mathbf{n}$ is the outward-pointing normal vector of the cell membrane. The attachment and detachment flows affecting the cytosolic concentrations $\mathbf{c}$ are contained in the reaction term $\boldsymbol{f}$.

This dynamics conserves the total number of proteins of each species because the proteins only diffuse in the cell and switch between cytosolic and membrane-bound states. For each protein species i we can define the "stoichiometric" vectors $\mathbf{s}_i^\mathrm{c}$, $\mathbf{s}_i^\mathrm{m}$ such that $\mathbf{s}_i^\mathrm{c} \cdot \mathbf{c}$ is the sum of the cytosolic components of species i and $\mathbf{s}_i^\mathrm{m} \cdot \mathbf{m}$

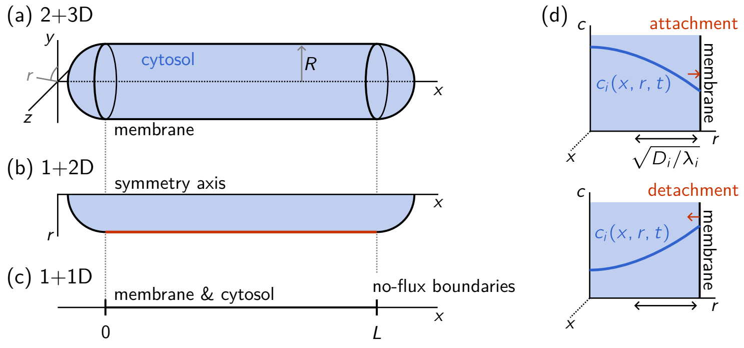

Figure S12. Approximation of the cellular geometry in the reaction–diffusion models. (a) The cell geometry is modeled by a spherocylinder of length L and radius R. (b) Employing the rotational symmetry of the analyzed protein patterns, the dynamics can be simulated in a radial slice of the full geometry. To classify the pattern type, the surface concentrations on the membrane are recorded along the red line. (c) If the radius R is small compared to the length scale of the cytosolic gradients perpendicular to the membrane, the reaction–diffusion dynamics may be projected onto a line. The cell poles are represented by no-flux boundary conditions. (d) Illustration of bulk-boundary coupling: Attachment and detachment of proteins onto and from the membrane induce gradients in the cytosolic concentration perpendicular to the membrane. The cytosolic diffusion constant D_i together with the reactive conversion rate in the cytosol λ_i, i.e., the corresponding reaction rate in $\boldsymbol{r}_\mathrm{c}$, sets the typical length scale $\sqrt{D_i/\lambda_i}$ on which the cytosolic concentration c_i varies.

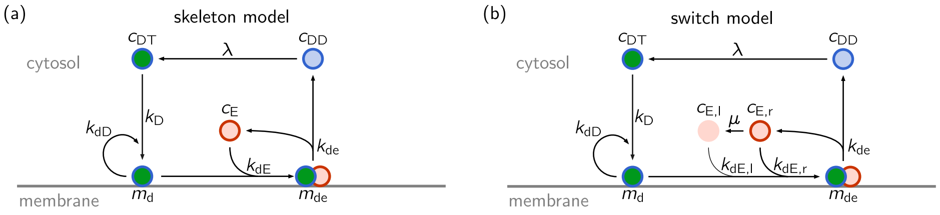

Figure S13. Reaction networks underlying the skeleton (a) and switch (b) models.

is the sum of the membrane components of this species. It follows that the conserved average total protein concentration $\bar{\rho}_i$ of the protein species i is given by

$$\bar{\rho}_i = \frac{1}{V}\left[\int_\mathrm{cytosol} \mathrm{d}^3x\, \mathbf{s}_i^\mathrm{c} \cdot \mathbf{c}(\mathbf{x}, t) + \int_\mathrm{membrane} \mathrm{d}^2x\, \mathbf{s}_i^\mathrm{m} \cdot \mathbf{m}(\mathbf{x}, t)\right], \tag{4}$$

where V is the cytosolic volume, i.e., the volume of the spherocylinder. We now specify the concentration fields and reaction terms for the skeleton and switch models.

8.1 The skeleton model

The skeleton model was introduced in Refs. [15, 16]. This model describes the cytosolic concentrations $\mathbf{c} = (c_\mathrm{DD}, c_\mathrm{DT}, c_\mathrm{E})$ of the ATP- and ADP-bound forms of MinD and cytosolic MinE [see Fig. S13(a)]. In the cytosol, nucleotide exchange reactivates ADP-bound MinD. This results in the reaction term

$$\mathbf{r}_\mathrm{c}(\mathbf{c}) = \begin{pmatrix} -\lambda c_\mathrm{DD} \\ \lambda c_\mathrm{DD} \\ 0 \end{pmatrix}. \tag{5}$$

On the membrane, the concentrations of MinD and MinDE complexes $\mathbf{m} = (m_\mathrm{d}, m_\mathrm{de})$ are modeled which undergo the reactions

$$\mathbf{r}_\mathrm{m}(\mathbf{m}, \mathbf{c}) = \begin{pmatrix} (k_\mathrm{D} + k_\mathrm{dD} m_\mathrm{d}) c_\mathrm{DT} - k_\mathrm{dE} c_\mathrm{E} m_\mathrm{d} \\ k_\mathrm{dE} c_\mathrm{E} m_\mathrm{d} - k_\mathrm{de} m_\mathrm{de} \end{pmatrix}. \tag{6}$$

The bulk-boundary coupling occurs via the reaction term

$$\boldsymbol{f}(\mathbf{m}, \mathbf{c}) = \begin{pmatrix} k_\mathrm{de} m_\mathrm{de} \\ -(k_\mathrm{D} + k_\mathrm{dD} m_\mathrm{d}) c_\mathrm{DT} \\ -k_\mathrm{dE} m_\mathrm{d} c_\mathrm{E} + k_\mathrm{de} m_\mathrm{de} \end{pmatrix}. \tag{7}$$

Parameter	Value	Unit
D_D	16	$\mu\mathrm{m}^2\mathrm{s}^{-1}$
D_E	10	$\mu\mathrm{m}^2\mathrm{s}^{-1}$
D_d	0.013	$\mu\mathrm{m}^2\mathrm{s}^{-1}$
D_de	0.013	$\mu\mathrm{m}^2\mathrm{s}^{-1}$
k_D	0.1	$\mu\mathrm{m}\,\mathrm{s}^{-1}$
k_dD	0.108/60	$\mu\mathrm{m}^3\mathrm{s}^{-1}$
k_dE	0.435/60	$\mu\mathrm{m}^3\mathrm{s}^{-1}$
k_de	1.925	s^{-1}
λ	6	s^{-1}
$\bar{\rho}_\mathrm{D}$	varied	$\mu\mathrm{m}^{-3}$
$\bar{\rho}_\mathrm{E}$	varied	$\mu\mathrm{m}^{-3}$

Table 2. Scaled parameters based on Ref. [16] used here for the skeleton model [see Fig. S13(a)]. Because the experiments presented in this article were performed at 37 °C, we evaluated the hydrolysis rate k_de at this temperature.

Moreover, the diffusion matrices are chosen as

$$\mathbf{D}_\mathrm{c} = \mathrm{diag}(D_\mathrm{D}, D_\mathrm{D}, D_\mathrm{E}), \qquad \mathbf{D}_\mathrm{m} = \mathrm{diag}(D_\mathrm{d}, D_\mathrm{de}). \tag{8}$$

Finally, the stoichiometric vectors are

$$\mathbf{s}_\mathrm{D}^\mathrm{c} = \begin{pmatrix}1\\1\\0\end{pmatrix}, \qquad \mathbf{s}_\mathrm{D}^\mathrm{m} = \begin{pmatrix}1\\1\end{pmatrix}, \qquad \mathbf{s}_\mathrm{E}^\mathrm{c} = \begin{pmatrix}0\\0\\1\end{pmatrix}, \qquad \mathbf{s}_\mathrm{E}^\mathrm{m} = \begin{pmatrix}0\\1\end{pmatrix}. \tag{9}$$

This model conserves the average total MinD and MinE concentrations $\bar{\rho}_\mathrm{D}$ and $\bar{\rho}_\mathrm{D}$:

$$\bar{\rho}_\mathrm{D} = \frac{1}{V}\left[\int_\mathrm{cytosol} \mathrm{d}^3x \left(c_\mathrm{DD}(\mathbf{x},t) + c_\mathrm{DT}(\mathbf{x},t)\right) + \int_\mathrm{membrane} \mathrm{d}^2x \left(m_\mathrm{d}(\mathbf{x},t) + m_\mathrm{de}(\mathbf{x},t)\right)\right], \tag{10}$$

$$\bar{\rho}_\mathrm{E} = \frac{1}{V}\left[\int_\mathrm{cytosol} \mathrm{d}^3x\, c_\mathrm{E}(\mathbf{x},t) + \int_\mathrm{membrane} \mathrm{d}^2x\, m_\mathrm{de}(\mathbf{x},t)\right]. \tag{11}$$

8.1.1 Parameter choice

As stated in Material and Methods, we use the skeleton model with the parameters determined in Ref. [16]. However, the nonlinear rates are rescaled to observe pattern formation at comparable values with the phase diagram found experimentally in this article (see Fig. 4 a). For the reader's convenience, we summarize the parameter values in Table 2.

8.2 The switch model

A striking feature of the skeleton model is that it only forms patterns if the average total concentration of MinE is lower than or roughly equal to the average concentration of MinD proteins (see Fig. 4a in the main text and Ref. [16, 17]). In contrast, the experiments of this study show that patterns form over a range of more than one order of magnitude of MinE concentrations.

A similar robustness of the Min protein patterns was observed in *in vitro* experiments in Ref. [17] The authors showed that the robustness is due to the conformational switch of MinE dimers between a latent (closed) and reactive (open) state. To describe this conformational switch, the "switch model" was introduced as an extension of the skeleton model [17] [see Fig. S13(b)]. Adding a latent MinE conformation to the skeleton model allows this inert state to act as a buffer at high MinE concentrations, which effectively reduces the active MinE concentration and confers robustness on the Min protein patterns.

While the switch model describes the same protein concentrations on the membrane as the skeleton model, two distinct concentration fields $c_\mathrm{E,r}$ and $c_\mathrm{E,l}$ are introduced in the cytosol for the reactive and latent MinE conformations. This results in $\mathbf{c} = (c_\mathrm{DD}, c_\mathrm{DT}, c_\mathrm{E,r}, c_\mathrm{E,l})$. The cytosolic reaction term is modified as

$$\mathbf{r}_\mathrm{c}(\mathbf{c}) = \begin{pmatrix}-\lambda c_\mathrm{DD}\\ \lambda c_\mathrm{DD}\\ -\mu c_\mathrm{E,r}\\ \mu c_\mathrm{E,r}\end{pmatrix}, \tag{12}$$

Parameter	Value	Unit
D_{D}	16	$\mu\mathrm{m}^2\mathrm{s}^{-1}$
D_{E}	10	$\mu\mathrm{m}^2\mathrm{s}^{-1}$
D_{d}	0.05	$\mu\mathrm{m}^2\mathrm{s}^{-1}$
D_{de}	0.05	$\mu\mathrm{m}^2\mathrm{s}^{-1}$
k_{D}	0.3	$\mu\mathrm{m}\,\mathrm{s}^{-1}$
k_{dD}	$7.5{\cdot}10^{-4}$	$\mu\mathrm{m}^3\mathrm{s}^{-1}$
$k_{\mathrm{dE,r}}$	0.75	$\mu\mathrm{m}^3\mathrm{s}^{-1}$
$k_{\mathrm{dE,l}}$	$5{\cdot}10^{-6}$	$\mu\mathrm{m}^3\mathrm{s}^{-1}$
k_{de}	1	s^{-1}
λ	5	s^{-1}
μ	20	s^{-1}
$\bar{\rho}_{\mathrm{D}}$	varied	$\mu\mathrm{m}^{-3}$
$\bar{\rho}_{\mathrm{E}}$	varied	$\mu\mathrm{m}^{-3}$
R	0.5	$\mu\mathrm{m}$
L	50	$\mu\mathrm{m}$

Table 3. Parameters used here for the switch model [see Fig. S13(b)] to describe the experimental concentration phase diagram.

where μ describes the rate of the conformational switch. The membrane reactions in the switch model read

$$\mathbf{r}_{\mathrm{m}}(\mathbf{m},\mathbf{c}) = \begin{pmatrix} (k_{\mathrm{D}} + k_{\mathrm{dD}} m_{\mathrm{d}}) c_{\mathrm{DT}} - (k_{\mathrm{dE,r}} c_{\mathrm{E,r}} + k_{\mathrm{dE,l}} c_{\mathrm{E,l}}) m_{\mathrm{d}} \\ (k_{\mathrm{dE,r}} c_{\mathrm{E,r}} + k_{\mathrm{dE,l}} c_{\mathrm{E,l}}) m_{\mathrm{d}} - k_{\mathrm{de}} m_{\mathrm{de}} \end{pmatrix}. \tag{13}$$

The attachment-detachment flows are given by

$$\boldsymbol{f}(\mathbf{m},\mathbf{c}) = \begin{pmatrix} k_{\mathrm{de}} m_{\mathrm{de}} \\ -(k_{\mathrm{D}} + k_{\mathrm{dD}} m_{\mathrm{d}}) c_{\mathrm{DT}} \\ -k_{\mathrm{dE,r}} c_{\mathrm{E,r}} m_{\mathrm{d}} + k_{\mathrm{de}} m_{\mathrm{de}} \\ -k_{\mathrm{dE,l}} c_{\mathrm{E,l}} m_{\mathrm{d}} \end{pmatrix}. \tag{14}$$

The matrix of cytosolic diffusion constants is extended to

$$\mathbf{D}_{\mathrm{c}} = \mathrm{diag}(D_{\mathrm{D}}, D_{\mathrm{D}}, D_{\mathrm{E}}, D_{\mathrm{E}}). \tag{15}$$

Moreover, the stoichiometric vectors read

$$\mathbf{s}^{\mathrm{c}}_{\mathrm{D}} = \begin{pmatrix} 1 \\ 1 \\ 0 \\ 0 \end{pmatrix}, \quad \mathbf{s}^{\mathrm{m}}_{\mathrm{D}} = \begin{pmatrix} 1 \\ 1 \end{pmatrix}, \quad \mathbf{s}^{\mathrm{c}}_{\mathrm{E}} = \begin{pmatrix} 0 \\ 0 \\ 1 \\ 1 \end{pmatrix}, \quad \mathbf{s}^{\mathrm{m}}_{\mathrm{E}} = \begin{pmatrix} 0 \\ 1 \end{pmatrix}. \tag{16}$$

In the switch model, the conservation of the average total MinD and MinE concentrations reads

$$\bar{\rho}_{\mathrm{D}} = \frac{1}{V}\left[\int_{\mathrm{cytosol}} \mathrm{d}^3x \left(c_{\mathrm{DD}}(\mathbf{x},t) + c_{\mathrm{DT}}(\mathbf{x},t)\right) + \int_{\mathrm{membrane}} \mathrm{d}^2x \left(m_{\mathrm{d}}(\mathbf{x},t) + m_{\mathrm{de}}(\mathbf{x},t)\right)\right], \tag{17}$$

$$\bar{\rho}_{\mathrm{E}} = \frac{1}{V}\left[\int_{\mathrm{cytosol}} \mathrm{d}^3x \left(c_{\mathrm{E,r}}(\mathbf{x},t) + c_{\mathrm{E,l}}(\mathbf{x},t)\right) + \int_{\mathrm{membrane}} \mathrm{d}^2x \, m_{\mathrm{de}}(\mathbf{x},t)\right]. \tag{18}$$

8.2.1 Parameter choice

The reaction parameters employed in the switch model to compare with the experiments in the main text, Fig. 3, and in Fig. S26 are discussed in Materials and Methods. Here in the SI, we analyze a second set of parameters and the change of the phase diagram under changing parameters, showing that the qualitative layout of the phase diagram is robust to parameter changes (see Fig. S14). These parameter values are given in Table 3.

9 Dimensional reduction of the cellular geometry

Because the reaction rates in the Min reaction network are not known from experiments, a large parameter study is necessary to identify a parameter region that reproduces the experimentally observed pattern types. For each parameter set the phase diagram of the average total MinD and MinE concentrations has to be simulated to analyze the patterns in the different concentration regions. To reduce the computational complexity, we reduce the full cellular geometry by using the cylindrical symmetry of the cell geometry.

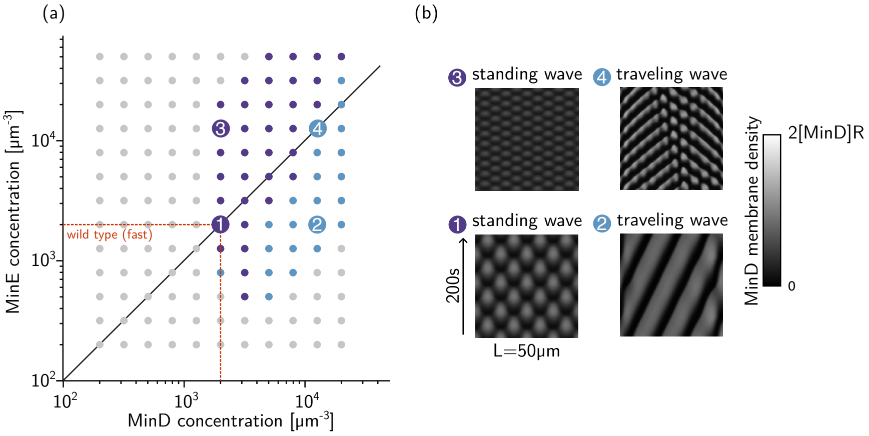

Figure S14. The simulated concentration phase diagram for filamentous cells using the parameters given in Tab. 3. The same simulation as for Fig. 3**b** in the main text (parameters given in Materials and Methods) is performed for the second set of parameters Tab. 3. (a) The qualitative layout of the phase diagram remains unchanged. The overall region of pattern formation is enlarged. The regions of traveling waves (blue) and standing waves (purple) lie at similar positions in the phase diagram. The wild-type concentration levels in fast-growth conditions are marked in red. (b) Four characteristic kymographs for the two pattern types are shown. The simulations are performed at the average total MinD and MinE concentrations labeled in panel (a). The kymograph shows the concentration of membrane-bound MinD (increasing concentrations from black to white).

9.1 Rotational symmetry

The *E. coli* cell geometry is (approximately) rotationally symmetric around the long axis of the bacteria. This is reflected by the rotational symmetry of the spherocylinder in our reaction–diffusion model. Moreover, the studied protein patterns are rotationally symmetric around the bacterial long axis as well after the initial transient of pattern growth. Thus, the protein concentration fields only depend on the axial position x along the long axis of the cell and on the radial position r:

$$\mathbf{c} = \mathbf{c}(x, r, t), \tag{19}$$

$$\mathbf{m} = \mathbf{m}(x, t). \tag{20}$$

Here, the protein concentrations on the membrane $\mathbf{m}$ are given in terms of a Monge parametrization.

The assumption of radial symmetry allows the simulation of the full dynamics including the bulk-boundary coupling between the cytosol and membrane as a 1+2D system instead of simulating the full 2+3D dynamics [see Fig. S12(b)]. Employing the form of the Laplacian in cylindrical coordinates, the cytosolic dynamics reduce to

$$\partial_t \mathbf{c}(x, r, t) = \mathbf{D}_\mathrm{c} \left(\partial_x^2 + \frac{1}{r}\partial_r + \partial_r^2 \right) \mathbf{c} + \mathbf{r}_\mathrm{c}(\mathbf{c}). \tag{21}$$

To determine the corresponding membrane dynamics, the Laplace-Beltrami operator has to be expressed in radial coordinates. The Laplace-Beltrami operator can be expressed using the surface derivative $\nabla_\mathrm{m} = (\mathbf{1} - \mathbf{n}\mathbf{n}^T)\nabla$ where $\mathbf{n}$ is the surface unit normal vector. The matrix $(\mathbf{1} - \mathbf{n}\mathbf{n}^T)$ projects the derivative onto the tangent plane of the surface. The Laplace-Beltrami operator then reads

$$\nabla_m^2 \bullet = (\mathbf{1} - \mathbf{n}\mathbf{n}^T)\nabla \left[(\mathbf{1} - \mathbf{n}\mathbf{n}^T)\nabla \bullet \right]. \tag{22}$$

Expressing every term in cylindrical coordinates and using that the concentration fields are independent of the angular coordinate, one finds from Eq. (2) the reduced membrane dynamics

$$\partial_t \mathbf{m}(x, t) = \mathbf{D}_\mathrm{m}(\nabla_{\mathrm{2D,m}}^2 + \frac{1}{r}\mathbf{e}_r \cdot \nabla_{\mathrm{2D,m}})\mathbf{m} + \mathbf{r}_\mathrm{m}(\mathbf{m}, \mathbf{c}|_\mathrm{m}). \tag{23}$$

The derivative $\nabla_{\mathrm{2D,m}} = (\mathbf{1} - \mathbf{n}\mathbf{n}^T)(\mathbf{e}_r\partial_r + \mathbf{e}_x\partial_x)$ is the tangential derivative of the one-dimensional membrane line in the two-dimensional radial slice (x, r) of the three-dimensional geometry. The vectors $\mathbf{e}_r$ and $\mathbf{e}_x$ are the unit vectors in the radial and axial direction.

9.2 Reduction to a one-dimensional model

In general, the extension of the cytosol in the radial direction cannot be neglected because membrane attachment and detachment induce gradients in the concentrations of the cytosolic protein species normal to the membrane

[see Eq. (3) and Fig. S12(d)]. The reduction onto a 1+2D model exactly reproduces these gradients (under the assumption of cylindrically symmetric patterns). If the gradients are shallow compared to the depth of the cytosol, the dynamics can be approximately reduced onto a line neglecting the extension of the cytosol perpendicular to the membrane [see Fig. S12(c)].

The length scale of the cytosolic gradients can be estimated by the typical diffusion length scales set by the ratio of the cytosolic diffusion coefficients and the cytosolic conversion rates λ and μ [18–20]. For the experimentally measured cytosolic diffusion coefficients and the conversion rates employed in our simulations (see Tables 2, 3), the diffusion length scales for MinD and MinE are $\sqrt{D_\mathrm{D}/\lambda} \approx 1.8\,\mu\mathrm{m}$ and $\sqrt{D_\mathrm{E}/\mu} \approx 0.7\,\mu\mathrm{m}$. Thus, the gradients occur on length scales larger than the typical radius of *E. coli* bacteria of about $0.5\,\mu\mathrm{m}$. Therefore, we approximate the cytosolic concentrations as constant normal to the membrane in realistic cell geometries, and the radial direction can be integrated out. The result is a model in a reduced line geometry where both the membrane and cytosolic concentrations only depend on the coordinate x along the long axis of the bacteria [see Fig. S12(c)].

Neglecting the spherical caps at the ends of the spherocylinder used to approximate the cellular geometry, we can integrate the cytosolic concentration fields over cross sections normal to the long axis x, and we arrive at

$$\begin{aligned} \partial_t 2\pi \int_0^R \mathrm{d}r\, r\mathbf{c}(r,x,t) &= 2\pi R\, \partial_t \tilde{\mathbf{c}}(x,t) && (24)\\ &= 2\pi R \boldsymbol{f}\big(\mathbf{m}(x,t), \mathbf{c}(R,x,t)\big) + 2\pi R D_\mathrm{c} \partial_x^2 \,\tilde{\mathbf{c}} + 2\pi R \mathbf{r}_\mathrm{c}(\tilde{\mathbf{c}}). && (25) \end{aligned}$$

Here, we introduced the cross-sectional (surface) concentrations $\tilde{\mathbf{c}}(x,t)$ that describe the protein content within a cross-section of the cylinder projected onto the circumference, i.e., on the membrane:

$$\tilde{\mathbf{c}}(x,t) = \frac{1}{2\pi R} \int_0^R \mathrm{d}r\, 2\pi r \mathbf{c}(r,x,t). \tag{26}$$

The reaction term $\mathbf{r}_\mathrm{c}$ is linear for the models we consider in this study. This allows it to be rewritten in terms of the reduced concentrations $\mathbf{c}'(x,t)$.

Under the assumption that the cytosolic gradients normal to the membrane are small in the considered cellular geometry, Eq. (26) simplifies to

$$\mathbf{c}(R,x,t) \approx \frac{2\pi R}{\pi R^2} \mathbf{c}'(x,t) = \frac{\mathbf{c}'(x,t)}{\zeta}, \tag{27}$$

introducing the bulk-surface ratio ζ. Inserting this approximation in Eq. (25), one finds the reduced one-dimensional cytosolic dynamics

$$\partial_t \mathbf{c}' \approx \mathbf{D}_\mathrm{c} \partial_x^2 \,\mathbf{c}' + \boldsymbol{f}\left(\mathbf{m}, \frac{\mathbf{c}'}{\zeta}\right) + \mathbf{r}_\mathrm{c}(\mathbf{c}') = \mathbf{D}_\mathrm{c} \partial_x^2 \,\mathbf{c}' + \tilde{\boldsymbol{f}}(\mathbf{m}, \mathbf{c}') + \mathbf{r}_\mathrm{c}(\mathbf{c}'). \tag{28}$$

By the same approximation, one finds from Eq. (2) the reduced membrane dynamics

$$\partial_t \mathbf{m} \approx \mathbf{D}_\mathrm{m} \partial_x^2 \,\mathbf{m} + \mathbf{r}_\mathrm{m}\left(\mathbf{m}, \frac{\mathbf{c}'}{\zeta}\right) = \mathbf{D}_\mathrm{m} \partial_x^2 \,\mathbf{m} + \tilde{\mathbf{r}}_\mathrm{m}(\mathbf{m}, \mathbf{c}'). \tag{29}$$

The reaction terms $\tilde{\boldsymbol{f}}$ and $\tilde{\mathbf{r}}_\mathrm{m}$ include the bulk-surface ratio in the reaction constants.

For the cylindrical geometry considered the bulk-surface ratio is given by $\zeta = R/2$. In a spherocylinder, the bulk-surface ratio is a little smaller due to the additional membrane area at the cell poles. For short cells of wild-type length $L \approx 3\,\mu\mathrm{m}$ and $R = 0.5\,\mu\mathrm{m}$ this results in $\zeta \approx 0.23\,\mu\mathrm{m}$ instead of $R/2 = 0.25\,\mu\mathrm{m}$. For filamentous cells, the bulk-boundary ratio approaches $R/2$. As the difference is small even for short cells, we use the value $\zeta = R/2 = 0.25\,\mu\mathrm{m}$ throughout this study for all cell lengths. Moreover, the cell poles are not modeled explicitly and only included as no-flux boundary conditions of the one-dimensional domain of length L in the simulation of Eqs. (28), (29).

Finally, in the reduced line dynamics the conservation laws for the average total (surface) concentrations $\bar{\rho}_i$ simply read

$$\bar{\rho}_i = \frac{1}{\zeta L} \int_0^L \mathrm{d}x \left(\mathbf{s}_i^\mathrm{c} \cdot \mathbf{c}'(\mathbf{x},t) + \mathbf{s}_i^\mathrm{m} \cdot \mathbf{m}(\mathbf{x},t)\right). \tag{30}$$

9.3 Numerical simulation

The reduced 1+2D model accounting for the realistic cell geometry is simulated using COMSOL Multiphysics 6.0 & 6.1. The simulations employ a finite-element discretization on a triangular Delaunay mesh with linear Lagrange elements for the cytosol (bulk) and quadratic elements on the membrane (boundary). Initially, the total protein mass is homogeneously distributed in the cytosolic states c_{DT} and c_{E} or $c_{\mathrm{E,l}}$ with weak random perturbations (uniformly distributed within $\pm 1\%$ around the homogeneous concentration). The 1+1D dynamics is solved using a uniform finite-differences discretization implemented in Mathematica 13.0 & 13.1 using second-order central differences. In the simulations, we employ the freedom in the choice of units, and the fast-growth wild-type concentrations are normalized to $\bar{\rho}_{\mathrm{D}} = \bar{\rho}_{\mathrm{E}} = 1000\,\mathrm{\mu m}^{-3}$ by scaling the nonlinear rates k_{dD}, k_{dE}, $k_{\mathrm{dE,r}}$, $k_{\mathrm{dE,l}}$ by a factor 1/2. Example notebooks for the simulation, as well as the configuration files for the COMSOL simulation of the switch model, are available at `https://github.com/henrikweyer/Min-in-vivo` [21].

10 Linear stability analysis of the homogeneous steady state and the onset of pattern formation

Denk et al. [17] used a linear stability analysis to show that the introduction of the MinE switch in the switch model increases the range of concentration ratios between MinD and MinE for which patterns can be observed in *in vitro* geometry.

The linear stability analysis of the homogeneous steady state describes the growth rates for weak perturbations around the homogeneous steady state. In the reduced 1+1D dynamics, the homogeneous steady state $(\mathbf{m}^*, \mathbf{c}'^*)$ is determined by

$$0 = \tilde{\boldsymbol{f}}(\mathbf{m}^*, \mathbf{c}'^*) + \mathbf{r}_{\mathrm{c}}(\mathbf{c}'^*), \tag{31a}$$
$$0 = \tilde{\mathbf{r}}_{\mathrm{m}}(\mathbf{m}^*, \mathbf{c}'^*), \tag{31b}$$
$$\rho_i = \mathbf{s}_i^{\mathrm{c}} \cdot \mathbf{c}'^*(\mathbf{x}, t) + \mathbf{s}_i^{\mathrm{m}} \cdot \mathbf{m}^*(\mathbf{x}, t), \tag{31c}$$

for each protein species i (MinD and MinE).

The dynamics of weak perturbations $(\delta\mathbf{m}, \delta\mathbf{c})$ around the homogeneous steady state is determined by the linearized dynamics

$$\partial_t \begin{pmatrix} \delta\mathbf{m} \\ \delta\mathbf{c} \end{pmatrix} = \left[\begin{pmatrix} \mathbf{D}_{\mathrm{m}} & 0 \\ 0 & \mathbf{D}_{\mathrm{c}} \end{pmatrix} \partial_x^2 + \mathbf{J}|_{\mathbf{m}^*, \mathbf{c}'^*} \right] \begin{pmatrix} \delta\mathbf{m} \\ \delta\mathbf{c} \end{pmatrix}, \tag{32}$$

where the Jacobian is given by

$$\mathbf{J} = \begin{pmatrix} \partial_{\mathbf{m}} \tilde{\mathbf{r}}_{\mathrm{m}} & \partial_{\mathbf{c}'} \tilde{\mathbf{r}}_{\mathrm{m}} \\ \partial_{\mathbf{m}} \tilde{\boldsymbol{f}} & \partial_{\mathbf{c}'} (\tilde{\boldsymbol{f}} + \tilde{\mathbf{r}}_{\mathrm{c}}) \end{pmatrix}. \tag{33}$$

The eigenmodes of the linearized dynamics Eq. (32) are Fourier modes with wavenumber q and growth rate $\sigma(q)$

$$\begin{pmatrix} \delta\mathbf{m}_q \\ \delta\mathbf{c}_q \end{pmatrix} = \begin{pmatrix} \delta\mathbf{m} \\ \delta\mathbf{c} \end{pmatrix} \mathrm{e}^{iqx + \sigma(q)t}. \tag{34}$$

The collection of growth rates $\sigma(q)$ is called the dispersion relation. It describes the exponential growth rate of a perturbation mode with wavenumber q. The growth rate is determined by inserting the ansatz Eq. (34) into the linearized dynamics Eq. (32). This yields the eigenvalue equation

$$\sigma(q) \begin{pmatrix} \delta\mathbf{m}_q \\ \delta\mathbf{c}_q \end{pmatrix} = \left[- \begin{pmatrix} \mathbf{D}_{\mathrm{m}} & 0 \\ 0 & \mathbf{D}_{\mathrm{c}} \end{pmatrix} q^2 + \mathbf{J}|_{\mathbf{m}^*, \mathbf{c}'^*} \right] \begin{pmatrix} \delta\mathbf{m}_q \\ \delta\mathbf{c}_q \end{pmatrix}. \tag{35}$$

Consequently, the dimension of the eigenvalue problem Eq. (35) determines the maximal number of solutions $\sigma(q)$, giving different branches $\sigma_i(q)$ of the dispersion relation. In the following, we denote by the dispersion relation always the eigenvalue $\sigma_i(q)$ for a certain wavenumber q which has the largest real part.

If the real part of the dispersion relation $\Re[\sigma(q)]$ is positive for any $q > 0$, the homogeneous steady state is laterally unstable. Perturbation modes with the corresponding wavenumber grow and the protein concentrations become inhomogeneous as a concentration pattern grows out of the homogeneous steady state.

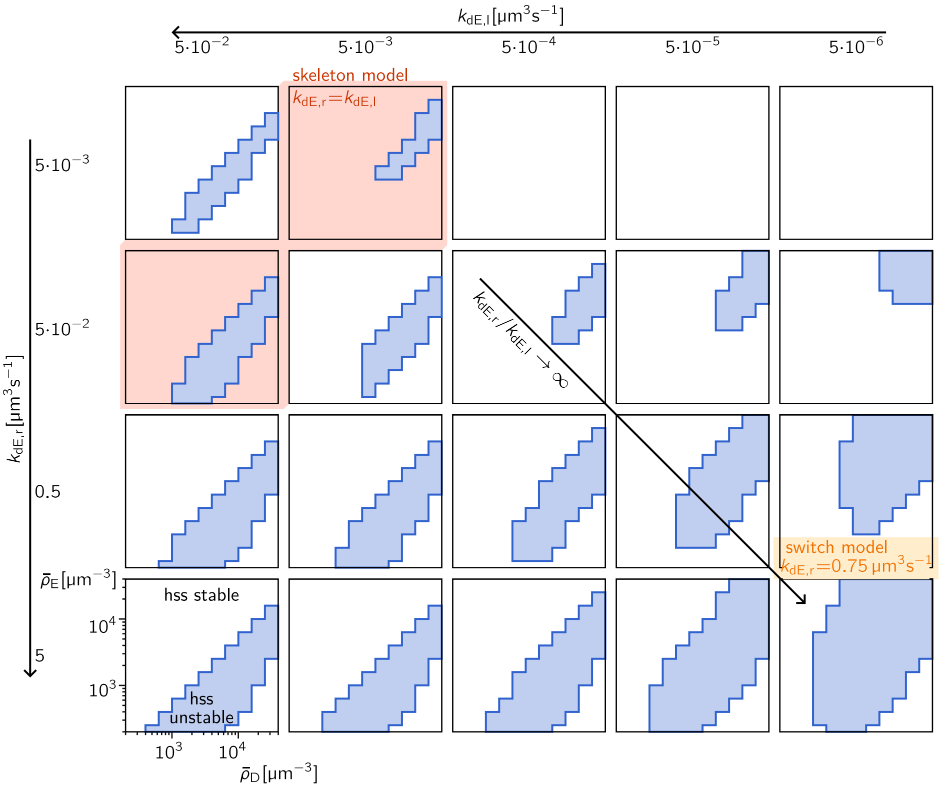

Figure S15. Linear stability analysis of the homogeneous steady state in the switch model. The concentration phase diagrams of the switch model in 1+1D geometry are shown for different combinations of the recruitment rates of the reactive and latent MinE states $k_{\mathrm{dE,r}}$ and $k_{\mathrm{dE,l}}$. The other parameters of the switch model are chosen as given in Table 3. The blue-shaded region denotes the average total MinD and MinE concentrations $\bar{\rho}_{\mathrm{D}}$ and $\bar{\rho}_{\mathrm{E}}$ for which the homogeneous steady state is unstable, i.e., $\Re[\sigma(q)] > 0$ for some wavenumber q. The same logarithmic concentration scale is used for each phase diagram. The switch model reduces to the skeleton model if $k_{\mathrm{dE,r}} = k_{\mathrm{dE,r}}$ (red-shaded phase diagrams). For comparison with the experimental data, we use $k_{\mathrm{dE,r}} = 0.75\,\mu\mathrm{m}^3\mathrm{s}^{-1}$ and $k_{\mathrm{dE,r}} = 5{\cdot}10^{-6}\mu\mathrm{m}^3\mathrm{s}^{-1}$ (green label; cf. Table 3).

10.1 Numerical calculation

The linear stability analysis is performed using Mathematica 13.1. First, Eqs. (31) are solved numerically to determine the homogeneous steady state concentrations $\boldsymbol{m}^*$, $\boldsymbol{c}'^*$. If several homogeneous steady states exist, the homogeneous steady state is selected that is locally stable, i.e., the one which fulfills $\Re[\sigma(0)] = 0$.* Then, the eigenvalue problem Eq. (35) is solved for discrete wavenumbers q. For each wavenumber, the eigenvalue with the largest real part is selected, and their collection for different wavenumbers gives the numerical dispersion relation. The Mathematica notebooks can be found under `https://github.com/henrikweyer/Min-in-vivo` [21].

10.2 Emergence of robust pattern formation due to the MinE switch

Figure S15 (see also Fig. 4 a in the main text) shows that the MinE switch enhances the pattern robustness in the switch model in the reduced 1+1D geometry, i.e., it enlarges the region of pattern formation with $\Re[\sigma(q)] > 0$ for some wavenumber q.† If the MinE recruitment rates $k_{\mathrm{dE,r}}$ and $k_{\mathrm{dE,l}}$ are equal, the skeleton model is recovered. This is seen by defining $c_{\mathrm{E}} = c_{\mathrm{E,r}} + c_{\mathrm{E,l}}$ in the reaction–diffusion equations (see Sec. 8). As the recruitment rate $k_{\mathrm{dE,l}}$ of latent MinE is reduced, the homogeneous steady state becomes laterally unstable at increased total MinE concentrations $\bar{\rho}_{\mathrm{E}}$. The increase of the recruitment rate of reactive MinE $k_{\mathrm{dE,r}}$ ensures that pattern formation is possible even at low total MinE and MinD concentrations. Thus, the pattern robustness can be increased in *in vivo* geometry—modeled here as the reduced 1+1D geometry—similarly as *in vitro* (cf. Ref. [17]).

* Each conservation law leads to one zero eigenvalue at $q = 0$.

† The wavenumber q can only take on multiples of π/L on a domain with finite length L and no-flux boundaries. The dispersion relation becomes continuous for $L \to \infty$. The resulting stability boundary estimates the stability region of finite systems with a length L that is large compared to the wavelength of the unstable wave modes. We choose $L = 50\,\mu\mathrm{m}$ in the numerical simulations, and the pattern wavelength $\Lambda \approx 10\,\mu\mathrm{m}$ is significantly shorter.

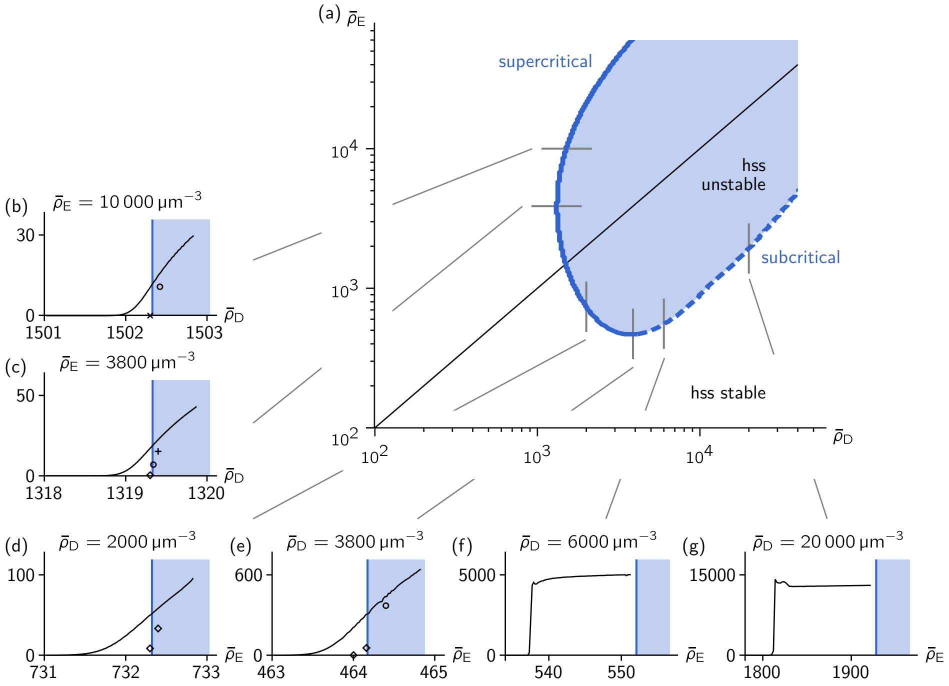

Figure S16. Classification of the onset of pattern formation. (a) Six different points along the onset of linear instability of the homogeneous steady state (hss; dark-blue border of the blue-shaded region) are crossed by tuning the average total MinD or MinE concentrations adiabatically (gray lines). (b–g) The pattern amplitude (of the MinD membrane concentration $m_{\mathrm{d}}+m_{\mathrm{de}}$) is tracked as the total concentration $\bar{\rho}_{\mathrm{D}}$ or $\bar{\rho}_{\mathrm{E}}$ is reduced adiabatically (black line) across the stability boundary of the homogeneous steady state (dark-blue border of the blue-shaded region). For those sweeps where the pattern amplitude decreases to zero continuously (b–e), discrete values of the total concentrations are chosen to perform numerical simulations at fixed average total concentrations for $5 \cdot 10^5$s (plus symbols), 10^6s (circles), $1.5 \cdot 10^6$s (crosses), or $2 \cdot 10^6$s (diamonds) starting from the concentration pattern determined in the adiabatic sweep for these values of the average total concentrations. The observed super- or subcritical behavior is marked in the concentration phase diagram as a solid and dashed dark-blue border of the instability region, respectively (a). The simulations are performed using COMSOL Multiphysics [22] for the 1+1D model with the parameters given in Table 3. The simulation is initialized with all proteins in the cytosolic species and sinusoidal spatial perturbations with the critical wavenumber q_{c} at the onset of pattern formation (see Sec. 10.4). The domain length is chosen as $L = 10 \cdot 2\pi/q_{\mathrm{c}}$. For the adiabatic sweeps, weak, constant source terms are introduced at $t = 10^4$s for species c'_{DD} (b–c) and $c'_{\mathrm{E,l}}$ (d–g). In (b–e), the source term is $-2{\cdot}10^{-6}\zeta\mu\mathrm{m}^{-3}\mathrm{s}^{-1}$, in (f) it is chosen as $-2{\cdot}10^{-5}\zeta\mu\mathrm{m}^{-3}\mathrm{s}^{-1}$, and in (g) as $-2{\cdot}10^{-4}\zeta\mu\mathrm{m}^{-3}\mathrm{s}^{-1}$ with the bulk-boundary ratio $\zeta = 0.25\,\mu\mathrm{m}$ (cf. Sec. 9.2).

10.3 Onset of instability in the concentration phase diagram

The onset of lateral instability can be super- or subcritical. In the first case, a patterned steady state only exists in the parameter region of lateral instability. The pattern amplitude decreases to zero continuously as the onset of lateral instability is approached by tuning some parameter. In contrast, if the onset is subcritical, patterns can form already before the onset of lateral instability where the homogeneous steady state is linearly stable. In this multistable regime (both patterned and homogeneous steady states exist), a sufficiently large stimulus is necessary to form a pattern.

McRD systems are known to show wide regimes of stimulus-induced pattern formation [23]. Therefore, we analyze the onset of pattern formation in the switch model as well for the chosen parameter set (cf. Table 3). To this end, the nonlinear dynamics of the 1+1D model is simulated using COMSOL Multiphysics [22] for concentration values just inside the region of lateral instability. The simulation is initialized with al proteins in the cytosolic species and sinusoidal spatial perturbations with the wavelength $2\pi/q_\mathrm{c}$ given by the critical wavenumber q_c at the onset of pattern formation (see Sec. 10.4). Then a weak, constant degradation term is introduced at $t = 10^4$s either for c'_DD or $c'_\mathrm{E,1}$ that slowly reduces the average total MinD or MinE concentration $\bar{\rho}_\mathrm{D}$ or $\bar{\rho}_\mathrm{E}$, respectively. Thereby, the average total concentration is adiabatically varied across the onset of linear instability (see Fig. S16).* For different paths in the concentration phase diagram [see Fig. S16(a)], we track the amplitude of the pattern amplitude while varying the average total concentration [see Figs. S16(b–g)]. As the steady-state pattern is oscillatory (standing or traveling wave), the amplitude is determined as the difference between the maximum and minimum pattern concentrations determined over a time period much larger than the local oscillation period. The setup files for the COMSOL simulation as well as the Mathematica notebook containing the data analysis are available at `https://github.com/henrikweyer/Min-in-vivo` [21].

Close to a supercritical onset of linear instability, the relaxation of the pattern onto the steady state becomes very slow. To determine the pattern amplitude more carefully, discrete values of the average total concentration are chosen, and simulations at fixed average total concentrations are performed starting from the concentration profiles determined in the adiabatic sweep for these concentration values [symbols in Figs. S16(b–e)].

These simulations show that the onset is supercritical for all tested concentrations at the onset of instability if the average total MinD concentration $\bar{\rho}_\mathrm{D}$ is low. In contrast, we find multistability and a subcritical onset of pattern formation at the onset of instability at a high average total MinD concentration. We propose that the onset of instability at low $\bar{\rho}_\mathrm{D}$ is indeed supercritical for all values of $\bar{\rho}_\mathrm{E}$ while the onset at high $\bar{\rho}_\mathrm{D}$ is subcritical [solid and dashed blue lines in Fig. S16(a)].

10.4 Quantification of the instability

The initial pattern formation process is dominated by the fastest-growing eigenmode with wavenumber q_c (see Fig. S17). This mode outgrows modes with other wavenumbers due to its larger exponential growth rate. Close to a supercritical onset of pattern formation the wavelength of the final pattern is as well determined by $2\pi/q_c$. The pattern behavior close to such an onset is formalized by the amplitude-equation approach [24, 25]. In the switch model, the onset of pattern formation at small average total concentration of MinD is supercritical (cf. previous section Sec. 10.3). Close to this onset, the final pattern wavelength is well described by the fastest-growing mode (see Fig. S18). It is an interesting future research direction to analyze this onset in detail and connect the numerical findings with the appropriate amplitude equations for systems with conservation laws and an oscillatory onset of instability [26–28].

While the real part of the dispersion relation determines the exponential growth or decay of an eigenmode, the imaginary part gives its oscillation frequency. Figure S18 shows that the period of the standing-wave oscillations in the switch model is again well described by $2\pi/\Im[\sigma(q_c)]$. However, further inside the pattern-forming region far away from the supercritical onset of pattern formation, neither the wavelength nor the oscillation period of the final Min patterns can be determined from the properties of the fastest-growing mode. To analyze the pattern properties throughout the whole phase diagram, an analysis of the fully developed nonlinear patterns is necessary.

* The data points from the initial period of the simulations are omitted in (b–g) as these only show the initial relaxation of the pattern from the chosen initial condition onto the steady-state pattern.

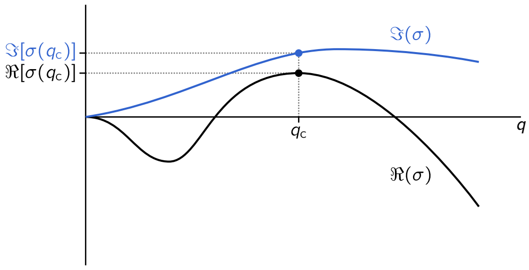

Figure S17. Quantification of the linear instability of the homogeneous steady state. The fastest-growing mode has the wavenumber q_c. The real part of the dispersion relation $\Re[\sigma(q_c)]$ determines its growth rate while the imaginary part $\Im[\sigma(q_c)]$ determines the oscillation frequency of the mode.

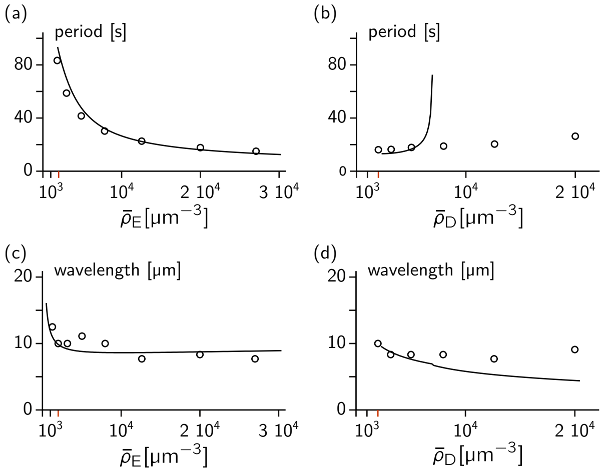

Figure S18. Comparison of the numerically measured wavelengths and periods (circles) with the wavelengths and periods determined from the fastest-growing mode in the linear stability analysis (lines). (a, b) The oscillation periods are analyzed for varying average total MinD and MinE concentrations. (c, d) The wavelengths are compared for varying average total MinD and MinE concentrations. In panels (a, c), the average total concentration of MinD is fixed at $\bar{\rho}_D = 3000\,\mu m^{-3}$. In panels (b, d), the average total concentration of MinE is fixed at $\bar{\rho}_E = 27\,000\,\mu m^{-3}$.

11 Parameter study of the switch model reveals standing- and traveling-wave patterns

After the discussion of the region of lateral instability in the $(\bar{\rho}_\mathrm{D}, \bar{\rho}_\mathrm{E})$-phase diagram in the last section, we go beyond the linear stability analysis here. In this section, we explore the type of patterns formed by the switch model by numerical simulation of the 1+1D model. The reduced 1+1D model is computationally less expensive and thus allows simulating the concentration phase diagram for different parameter combinations.

To determine a parameter region that not only reproduces the robustness of pattern formation but also the pattern types, we classify the patterns obtained in the numerical simulations and compare the resulting $(\bar{\rho}_\mathrm{D}, \bar{\rho}_\mathrm{E})$-phase diagrams for different reaction rates. In the following, we first explain the classification of standing- and traveling-wave patterns. Afterward, the results of the parameter study are presented.

11.1 Pattern classification

For each set of rate parameters and average total concentration values $\bar{\rho}_\mathrm{D}, \bar{\rho}_\mathrm{E}$ the time evolution of the 1+1D switch model is simulated for 3000 s starting from small random perturbations around the homogeneous steady state. The domain length is $L = 50\,\mu\mathrm{m}$. A notebook with the simulation setup in Mathematica is available under `https://github.com/henrikweyer/Min-in-vivo` [21]. The pattern type obtained at the end of this simulation is classified by analysis of the kymograph of the total MinD membrane concentration $m_\mathrm{d}(x,t) + m_\mathrm{de}(x,t)$ over the last 1000 s. We observe standing- and traveling-wave patterns.

Importantly, the transition from traveling-wave towards standing-wave patterns occurs gradually: The peak concentration of the traveling wave starts oscillating until it decomposes into single oscillating stripes. Moreover, extended standing-wave regimes appear where traveling waves collide with each other or with the domain boundary close to the transition between the pattern types.

Because the transition is gradual, the exact position of the transition line depends on the specific classification criterion. Therefore, in the main text (see Fig. 4 b) the standing- and traveling-wave regions are depicted to gradually transition into each other. This is in agreement with the experimental observation. For simplicity, we will specify a sharp transition here, separating regions of the concentration phase diagrams that show more standing wave-like from those showing more traveling wave-like patterns.

To distinguish traveling-wave patterns from stripe oscillations, we build on the observation that traveling waves moving through the system result in diagonal lines of high surface concentration in the kymograph while stripe oscillations produce isolated spatiotemporal domains of high concentration (see Fig. S19). Consequently, for each oscillation period of length T there is one high-concentration domain expected for a traveling-wave pattern [see Fig. S19(b)]. The total number of high-concentration domains N in a kymograph for a temporal period ΔT fulfills $N \approx \Delta T/T$.* In contrast, for a standing-wave pattern forming one stripe at midplane, three disconnected high-concentration domains are expected during one oscillation cycle. Shorter wavelengths Λ of the standing-wave pattern result in more disconnected domains for each oscillation period [see Fig. S19(a)]. One expects a total number of high-concentration domains $N \approx 2L/\Lambda\, \Delta T/T$. Accordingly, we classify a pattern as a traveling wave if

$$N < (L/\Lambda + 2)\Delta T/T \tag{36}$$

because a domain showing a standing-wave pattern in half the domain and a traveling-wave pattern in the other half fulfills $N \approx (L/\Lambda + 1)\Delta T/T$ if one has $\Delta T/T \gg 1$ and $L/\Lambda \gg 1$.

Instead of showing disconnected high-concentration domains, an (inverted) standing-wave pattern can manifest in disconnected low-concentration domains while the high-concentration domains are connected [see Fig. S19(c)]. This type of standing-wave pattern occurs for average total concentrations very close to the onset of pattern formation at high average total MinD concentrations. Therefore, if the number of high-concentration domains lies below the threshold Eq. (36), we test the same threshold for the number of low-concentration domains [see Fig. S19(b, c)].

To determine the wavelength Λ, for each time slice of the kymograph the individual structure factor is calculated via the discrete Fourier transform (DFT). The overall structure factor is then determined by averaging the individual structure factors over time. Because the simulation domain has reflective boundaries while the DFT assumes a periodic function, the DFT of each time slice is calculated for the concatenation of the pattern and its reflection. From the structure factor, we determine the dominant Fourier mode $q = 2\pi/\Lambda$ of the pattern as the mode giving the largest value in the structure factor.

* The finite temporal length of the kymograph leads to the cutoff of a few domains which increases N. Moreover, boundary effects at the ends of the domain lead to a weak oscillation of the concentration maximum of the traveling wave for some parameters, resulting in more than one high-concentration domain for each traveling wave.

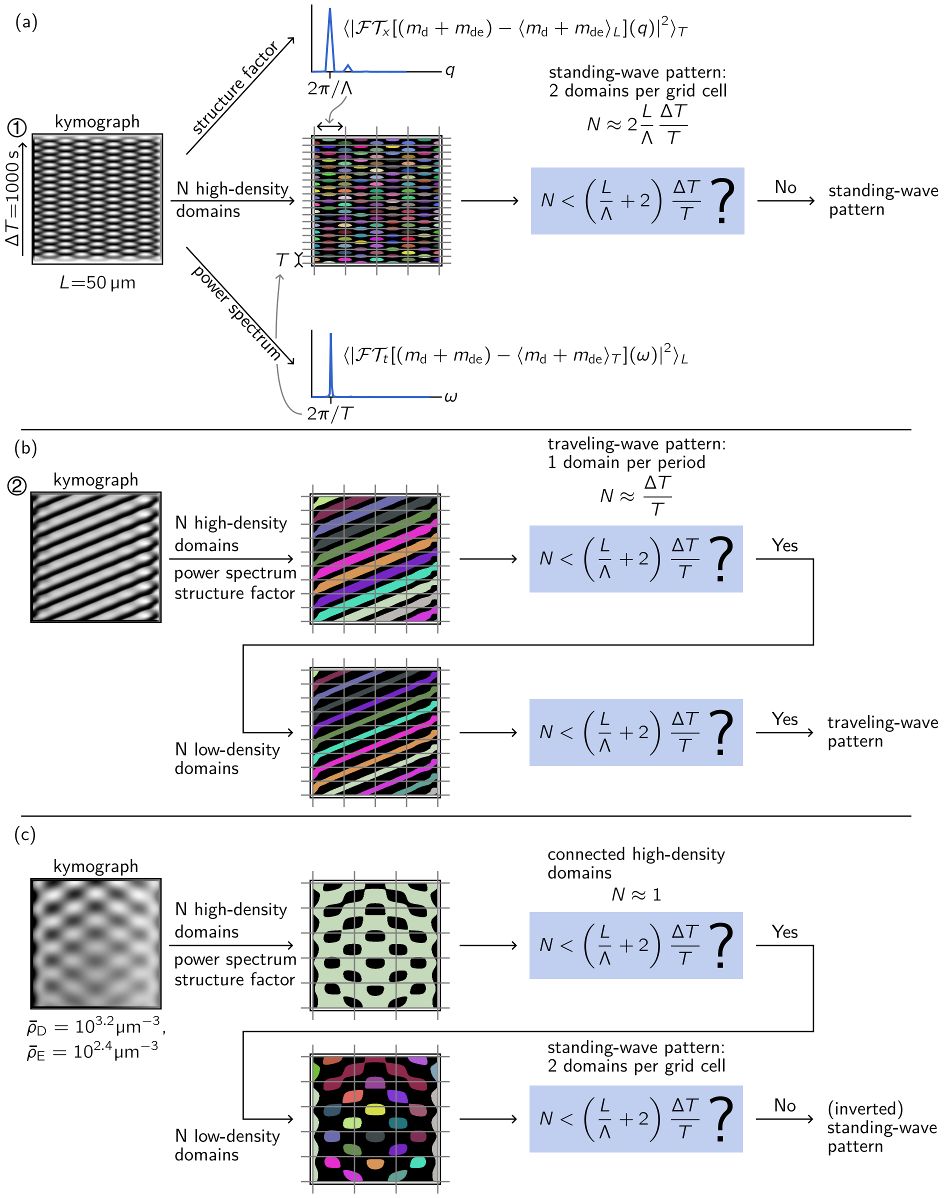

Figure S19. Classification algorithm distinguishing (inverted) standing-wave (a, c) from traveling-wave patterns (b), exemplified for the data points 2 and 1 as well as the simulation with $\bar{\rho}_D = 2{\cdot}10^{3.2}\mu m^{-3}$, $\bar{\rho}_D = 2{\cdot}10^{2.4}\mu m^{-3}$ in Fig. 4 b. The wavelength and period of the pattern are determined by discrete Fourier transforms of the kymograph of the MinD membrane concentration $m_d + m_{de}$ (shown in grayscale with white denoting regions of high surface concentration). Then, the kymograph is binarized, and the distinct high-concentration domains are counted. Each distinct high-concentration domain is colored differently. If a standing-wave pattern fills half of the domain while the other half shows a traveling-wave pattern, one expects $N \approx (L/\Lambda + 1)\Delta T/T$ disconnected high-concentration domains if the pattern fulfills $\Delta T/T \gg 1$ and $L/\Lambda \gg 1$. Accordingly, we classify a pattern as (predominantly) standing-wave type if it gives $N \geq (L/\Lambda + 2)\Delta T/T$. To account for standing-wave patterns whose high-concentration domains are connected while the low-concentration domains are disconnected, in a second step the number of the low-concentration domains is tested against the same criterion.

Similarly, the power spectrum of the kymograph is calculated to determine the oscillation period T. It is computed as the average of the individual power spectra of the time traces at each single spatial point of the pattern which are calculated using the DFT (no concatenation with the reflected time trace). Again the period T is determined from the frequency $\omega = 2\pi/T$ showing the largest value in the power spectrum.

To count the high-concentration domains in the kymograph, the surface concentration is binarized using the standard implementation in Mathematica 13.1 based on cluster variance maximization. From the binarized kymograph, the disconnected high-concentration domains are determined and counted. To count the number of low-concentration domains, the binarized concentrations are inverted before the disconnected domains are counted.

Lastly, if the oscillation period is larger than $\Delta T/2 = 500\,\mathrm{s}$, the pattern is not classified. Moreover, a parameter combination is marked as not showing a pattern if the amplitude in the kymograph is lower than 5% of the minimal pattern membrane concentration:

$$\frac{\max_{\Delta T,L}(m_\mathrm{d} + m_\mathrm{de}) - \min_{\Delta T,L}(m_\mathrm{d} + m_\mathrm{de})}{\min_{\Delta T,L}(m_\mathrm{d} + m_\mathrm{de})} < 0.05\,. \tag{37}$$

In summary, the classification exploits the shape differences of high- and low-concentration domains in the pattern kymographs. This classification only becomes unreliable close to the onset of pattern formation at high average total MinD concentrations because of the long wavelength and period of the patterns. Thus, the kymographs only contain a few pattern domains to compare. Therefore, the inverted standing-wave patterns that occur close to this onset of pattern formation are not clearly distinguished from traveling waves. If less than two periods are contained in the kymograph, the pattern is not classified into either of the two categories.

11.1.1 Pattern classification in the 1+2D model

For the pattern classification in the 1+2D model (see Fig. 4 b), the same classification is used. For the kymograph the membrane concentration $m_\mathrm{d} + m_\mathrm{de}$ is recorded along the flat membrane of the cylinder (red line in Fig. S12). One has to change the criterion for the stationary state Eq. (37). Due to the reduced local bulk-surface ratio at the cell poles (the spherical caps of the spherocylinder), the stationary state of the system shows inhomogeneous membrane concentrations, even if the system does not undergo a dynamic instability [29]. Therefore, the criterion Eq. (37) is tested for the MinD membrane concentration $m_\mathrm{d} + m_\mathrm{de}$ only in the middle third of the domain $x \in [L/3, 2L/3]$.

11.2 Parameter study

To find model parameters that reproduce the observed pattern types, our starting points were the study of the skeleton model in *in vivo* geometry performed in Ref. [16] and the linear stability analysis of the switch model for a *in vitro* geometry given in Ref. [17].

Let us first discuss the parameters employed by Denk et al. [17] for the switch model. In their work, the recruitment rates of the reactive and latent MinE states $k_\mathrm{dE,r}$ and $k_\mathrm{dE,l}$ were tuned over several orders of magnitudes. Moreover, we note that a uniform scaling of all nonlinear rates k_dD, $k_\mathrm{dE,r}$, and $k_\mathrm{dE,l}$ by a factor ε corresponds to a scaling of all densities by a factor $1/\varepsilon$, i.e., a rescaling of the units for measuring the densities. We use this freedom of scaling to move the pattern-forming region to similar densities as observed in the experiments. To this end, we set $k_\mathrm{dD} = 0.01\,\mathrm{\mu m^3 s^{-1}}$ (divided by a factor two compared to Ref. [17]) as well as choose $k_\mathrm{dE,r} = 1\,\mathrm{\mu m^3 s^{-1}}$ and $k_\mathrm{dE,r}/k_\mathrm{dE,l} = 10^{-4}$. In addition, we multiply the hydrolysis rate k_de by 5 for the same reason, resulting in $k_\mathrm{de} = 5 \cdot 0.34\mathrm{s^{-1}}$. Moreover, the cytosolic diffusion coefficients measured *in vivo* are used [30] (cf. Table 3). These parameters produce traveling waves throughout almost the whole concentration phase diagram [see Fig. S20(a)]. Only close to the onset of instability at low average total MinD and high MinE concentrations, a standing-wave pattern is observed.

Similarly, we only observe traveling waves when starting from the skeleton model parameters [see Fig. S20(b)]. To use the skeleton model parameters, we extend the parameters from Table 2 by the MinE switch. In order to reproduce the overall instability region, the concentrations are only scaled by a factor of 4 instead of 60 as in Table 2. Moreover, the (scaled) MinE-recruitment rate $k_\mathrm{dE} = 0.435/4\mathrm{\mu m^3 s^{-1}}$ is replaced by the rate $k_\mathrm{dE,r} = 10k_\mathrm{dE}$ for the reactive MinE state and $k_\mathrm{dE,l} = 10^{-3}k_\mathrm{dE}$ for the latent MinE state. The rate of the conformational switch $\mu = 100\,\mathrm{s^{-1}}$ is chosen as by Denk et al. [17]. In the following, we denote this parameter set as "extended skeleton model".

For the skeleton model, it was argued that the MinD self-recruitment rate has to be high to form pronounced pole-to-pole oscillations in wild-type geometry [16]. We find that this constraint does not carry over to the switch

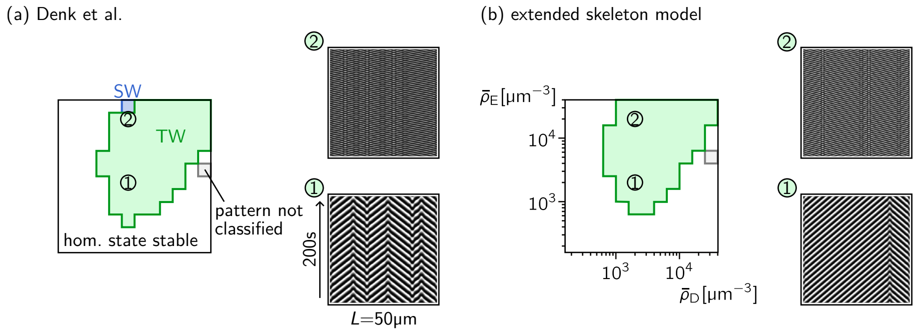

Figure S20. Pattern classification as standing (SW, blue-shaded region) and traveling waves (TW, green-shaded region) in the concentration phase diagrams of the switch model (a) with parameters following Denk et al. [17] and the extended skeleton model (b). In the white region of the phase diagrams the numerical simulation evolves towards the homogeneous stationary state. Patterns not classified are shown as gray-shaded regions (cf. 11.1). (a) The switch model is simulated with the parameters used by Denk et al. [17]. In contrast to the choice of the *in vitro* bulk diffusion coefficients in this work, we choose the cytosolic diffusion coefficients measured *in vivo* (cf. Tables 2, 3). Moreover, the MinE recruitment rates were varied in the study by Denk et al.. We choose $k_{\mathrm{dE,r}} = 1\,\mu\mathrm{m}^3\mathrm{s}^{-1}$, $k_{\mathrm{dE,r}}/k_{\mathrm{dE,l}} = 10^{-4}$, as well as set $k_{\mathrm{dD}} = 0.01\,\mu\mathrm{m}^3\mathrm{s}^{-1}$. These values as well as the scaled hydrolysis rate $k_{\mathrm{de}} = 5 \cdot 0.34\mathrm{s}^{-1}$ are employed to approximately reproduce the overall region of lateral instability in the concentration phase diagram. (a) The extended skeleton model describes a parameter set for the switch model which is obtained by extending the parameters of the skeleton model given in Table 2 by rates for a MinE switch. First, the nonlinear rates are scaled by a factor of 4 instead of 60 as in Table 2 in order to move the pattern-forming region to MinD and MinE concentrations comparable with the experimental phase diagram. Second, the (scaled) MinE-recruitment rate $k_{\mathrm{dE}} = 0.435/4\mu\mathrm{m}^3\mathrm{s}^{-1}$ is split into the rate $k_{\mathrm{dE,r}} = 10k_{\mathrm{dE}}$ for the reactive MinE state and $k_{\mathrm{dE,l}} = 10^{-3}k_{\mathrm{dE}}$ for the latent MinE state. For both parameter sets example kymographs of traveling wave patterns for two different concentration combinations are shown. The MinD membrane concentration $m_{\mathrm{d}} + m_{\mathrm{de}}$ is shown in grayscale with white denoting high membrane concentration.

model. Figure S21 shows that the switch model only forms standing-wave patterns (giving rise to pole-to-pole oscillations in short cells of wild-type length) in large regions of the phase diagram if the MinD self-recruitment rate k_{dD} is sufficiently low compared to the rate of spontaneous MinD membrane attachment k_{D}. Consequently, the MinE switch allows for standing-wave patterns already if the MinD self-recruitment is weak. The values of the parameters k_{D} and k_{dD} used by Denk et al. and in the extended skeleton model are marked in Fig. S21 for comparison. Note however that the other parameters not varied are fixed to the values given in Table 3 different from the values used by Denk et al. and in the extended skeleton model.

Moreover, Figure S22 shows that a reduced rate of the conformational switch μ—although still larger than all other rates—also enlarges the standing-wave region in the concentration phase diagram.

Taken together, this selection of the parameter sweeps explains the most important parameter changes that lead to the final parameter set given in Table 3: The nonlinear MinD attachment, i.e., the MinD self-recruitment is strongly reduced as well as the rate of the conformational MinE switch. It will be an interesting task for future research to uncover the mechanistic processes underlying standing-wave and traveling-wave formation based on the overview of the parameter space we give here.

11.2.1 Wavelength and period of the nonlinear patterns

Finally, after matching the region of pattern formation as well as the observed pattern types, the third layer of detail is the quantitative comparison of the pattern wavelength and period.

The analysis of the pattern wavelength for different rate parameters shows that it is almost constant throughout the concentration phase diagram (see Figs. S23, S24). The parameter study Fig. S23 shows that the wavelength is strongly influenced by the self-recruitment rate of MinD k_{dD}. This behavior is similar to the observation in the skeleton model that the self-recruitment rate sets the position where the new polar zone starts to grow [16]. Thus, the value of k_{dD} is chosen to reproduce the experimentally determined wavelength. The value of the linear attachment rate k_{D} then follows from the matching of the pattern types. Increasing the membrane diffusion coefficients $D_{\mathrm{d}} = D_{\mathrm{de}}$ leads to a weak increase in the pattern wavelength (see Fig. S24).

While reproducing the experimental wavelength, also the oscillation period should fit the experiment. The length

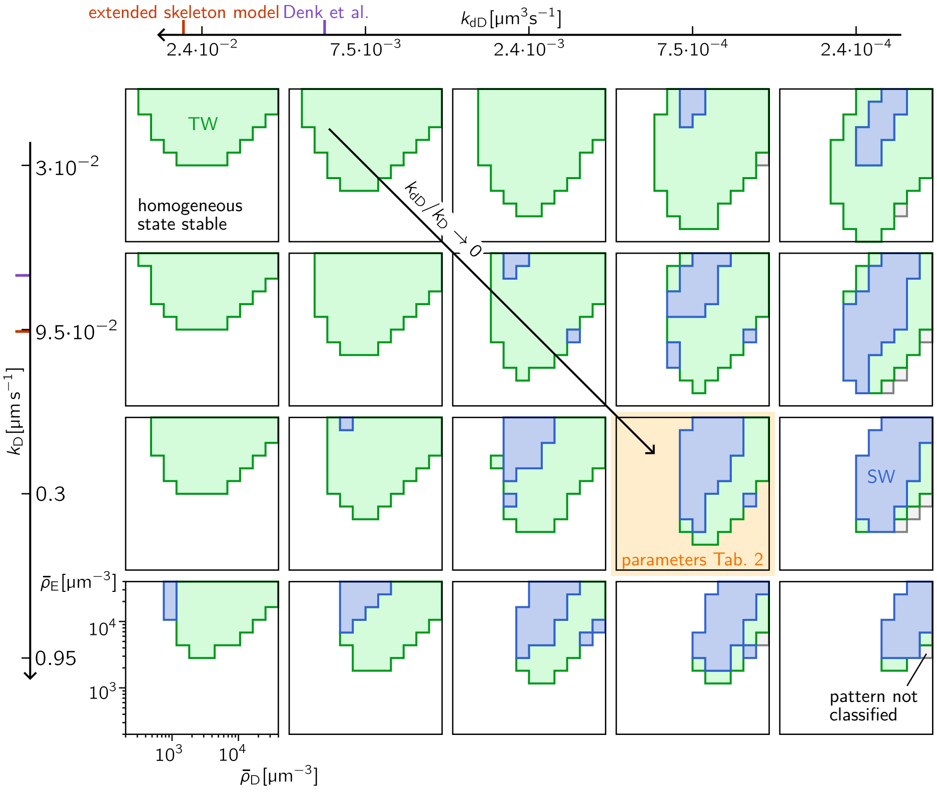

Figure S21. The effect of MinD attachment rates k_D and k_{dD} on the pattern types in the concentration phase diagram. For each combination of values for the rates k_D and k_{dD}, the 1+1D model is simulated for different combinations of average total concentrations $\bar{\rho}_D$ and $\bar{\rho}_E$. For each combination of concentrations, the pattern type is determined as explained in Sec. 11.1. The result is a concentration phase diagram for each rate combination showing the region of standing waves (blue-shaded), traveling waves (green-shaded), and patterns not classified (gray-shaded). For the other combinations of average total concentrations, the homogeneous state is stable. The parameters kept constant are set according to Table 3. The phase diagram corresponding to the parameter set given in Table 3 is shaded in orange. Additionally, the values of k_D and k_{dD} based on the analysis by Denk et al. and in the extended skeleton model are marked on the parameter axes in purple and red, respectively (see Sec. 11.2).

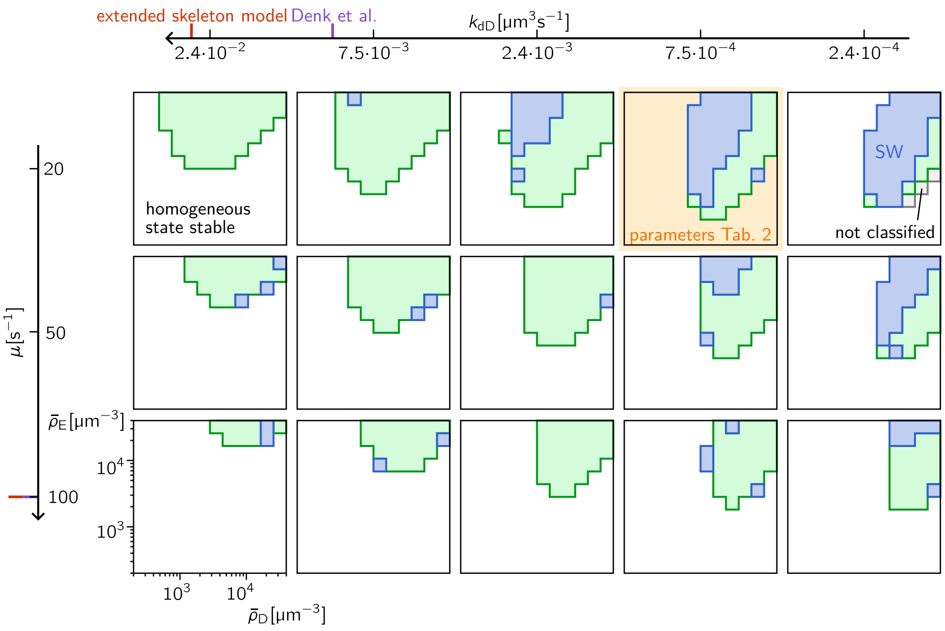

Figure S22. The effect of the rate of the conformational switch on the pattern types in the concentration phase diagram. The color code is the same as in Fig. S21. Again, the parameters not varied are given in Table 3. The values of μ and k_{dD} based on the analysis by Denk et al. and in the extended skeleton model are marked on the parameter axes in purple and red, respectively (see Sec. 11.2).

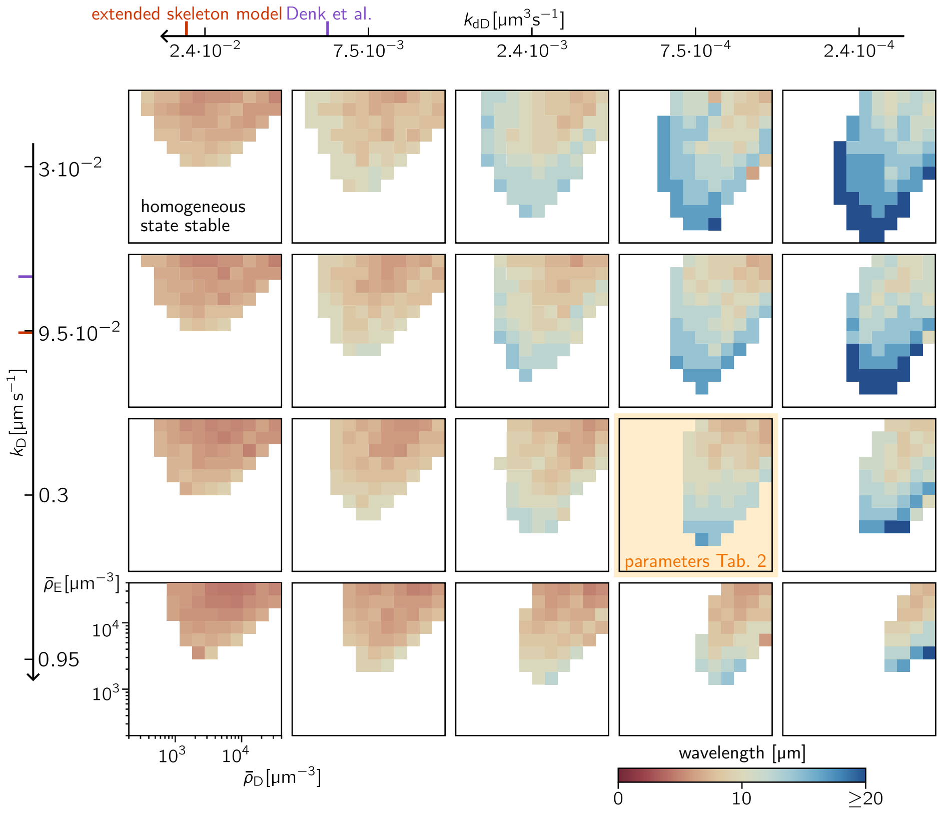

Figure S23. Dependence of the pattern wavelength on the linear attachment and self-recruitment rates k_{D}, k_{dD} of MinD. The fill color denotes the observed pattern wavelength according to the color legend (bottom right). An increasing self-recruitment rate k_{dD} decreases the observed pattern wavelength. The phase diagram corresponding to the chosen parameter set (cf. Table 3) is marked in orange. Again, the parameters not varied are given in Table 3. The values of k_{D} and k_{dD} based on the analysis by Denk et al. and in the extended skeleton model are marked on the parameter axes in purple and red, respectively (see Sec. 11.2).

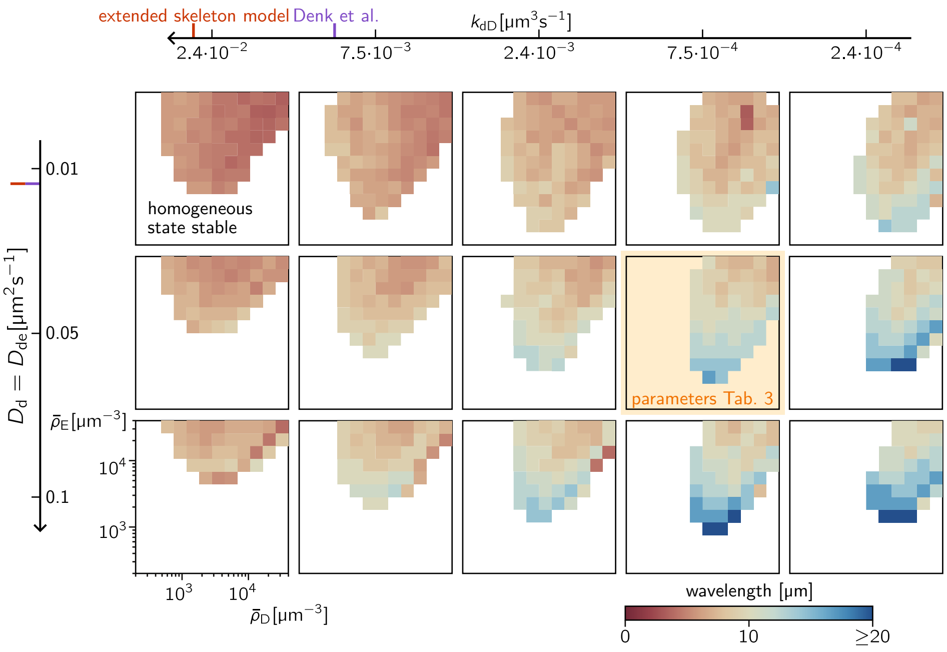

Figure S24. Dependence of the pattern wavelength on the membrane diffusion coefficients $D_d = D_{de}$ and the self-recruitment rate k_{dD} of MinD. The fill color denotes the observed pattern wavelength according to the color legend (bottom right). The phase diagram corresponding to the parameter set Table 3 is marked in orange. The parameters not varied are given in Table 3. The values of $D_d = D_{de}$ and k_{dD} based on the analysis by Denk et al. and in the extended skeleton model are marked on the parameter axes in purple and red, respectively (see Sec. 11.2).

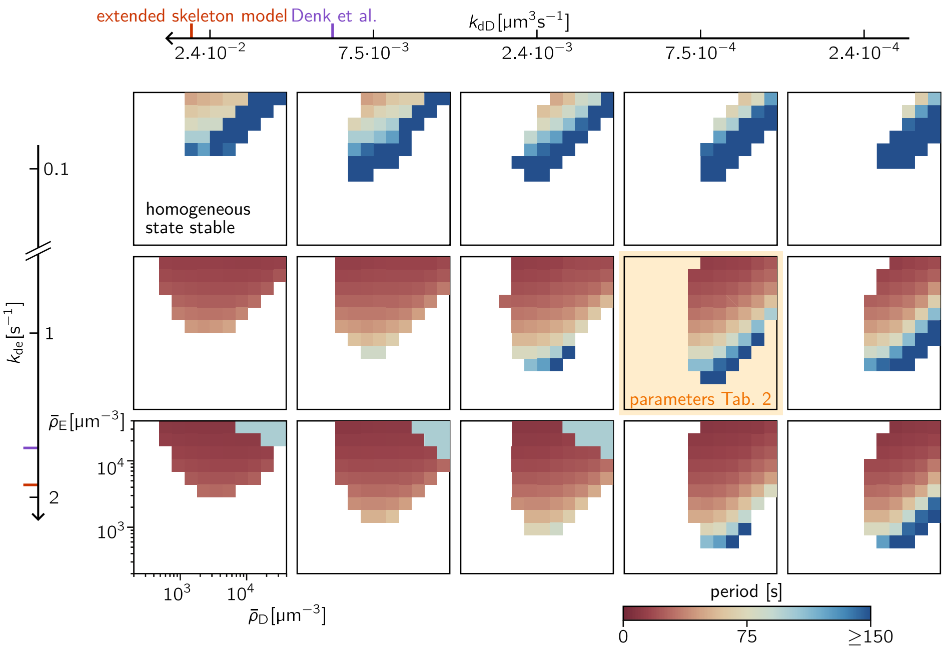

Figure S25. Dependence of the local oscillation period on the hydrolysis rate k_{de} and the MinD self-recruitment rate k_{dD}. The fill color denotes the oscillation period according to the color legend (bottom right). The hydrolysis rate influences both the onset of pattern formation at low average total MinE concentrations and the oscillation period. At the high hydrolysis rate, $k_{de} = 2\,\mathrm{s}^{-1}$ and high average total MinD and MinE concentrations an abrupt change in the pattern oscillation period can be observed. Again, the parameters not varied are given in Table 3. The values of k_{de} and k_{dD} based on the analysis by Denk et al. and in the extended skeleton model are marked on the parameter axes in purple and red, respectively (see Sec. 11.2).

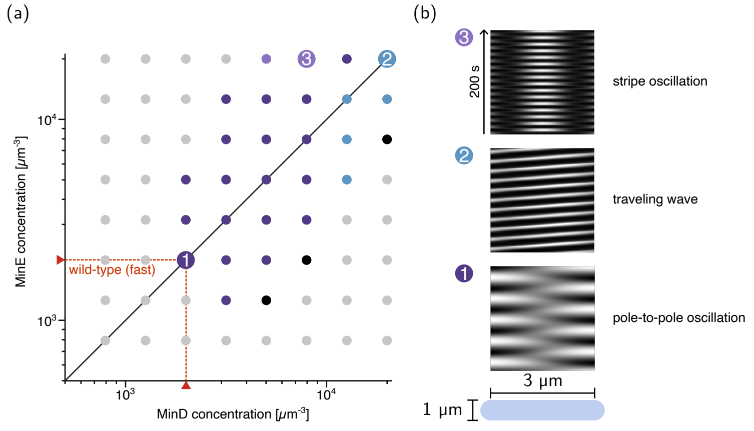

Figure S26. The simulated concentration phase diagram for cells of wild-type length. The same simulation as for Fig. 3**b** in the main text (parameters given in Materials and Methods) is performed in a simulation domain with a cell length $L = 3\,\mu\text{m}$ [see panel (b)]. (a) The concentration phase diagram shows pole-to-pole oscillations (dark purple), stripe oscillations (standing-wave-like patterns, light purple), and traveling waves (blue). The shading indicates that the transition between pole-to-pole oscillations and traveling waves is gradual. The points at concentrations $([\text{MinD}], [\text{MinE}]) = 2(10^{3.4}, 10^{2.8}), 2(10^{3.6}, 10^{3}), 2(10^{4}, 10^{3.6})\mu\text{m}^{-3}$ (black) were classified as inverted standing-wave pattern (see Sec. 11.1). Their kymographs cannot be clearly attributed to pole-to-pole oscillations or traveling waves. The wild-type concentration levels in fast-growth conditions are marked in red. (b) Three characteristic kymographs for the three pattern types are shown. The simulations are performed at the average total MinD and MinE concentrations labeled in panel (a). The kymograph shows the concentration of membrane-bound MinD (increasing concentrations from black to white). The simulated cell geometry is depicted at the bottom.

and time scales of the system are connected via the diffusion coefficients, and it is not obvious that both can be tuned independently as the values of the cytosolic diffusion coefficients are fixed to their experimentally measured values. We find in the switch model that the hydrolysis rate k_{de} tunes the oscillation period (see Fig. S25) while it does not strongly affect the pattern wavelength. The value chosen for k_{de} is determined such that the region of pattern formation, as well as the oscillation period, is reproduced.

12 The concentration phase diagram for wild-type-length cells

Figure 3 in the main text compares the experimental and simulated phase diagram in filamentous cells in order to analyze the intrinsic pattern-formation behavior of the *in vivo* Min system. Here, we show that for most concentrations in the phase diagram a short, wild-type cell length set to $L = 3\,\mu\text{m}$ in the simulation leads to the formation of a pole-to-pole oscillation of the Min proteins. Figure S26 shows that traveling waves occur in short cells only at high MinD overexpression. At high MinE overexpression, stripe oscillations (standing-wave pattern) can occur even in short cells.

References

[1] X.-t. Li, Y. Jun, M. J. Erickstad, S. D. Brown, A. Parks, D. L. Court, and S. Jun, "tCRISPRi: tunable and reversible, one-step control of gene expression", Scientific Reports **6** (2016).

[2] N. Palanisamy, M. A. Öztürk, E. B. Akmeriç, and B. Di Ventura, "C-terminal eYFP fusion impairs MinE function", Open Biol. **10**, 200010 (2020).

[3] F. Si, D. Li, S. E. Cox, J. T. Sauls, O. Azizi, C. Sou, A. B. Schwartz, M. J. Erickstad, Y. Jun, X. Li, and S. Jun, "Invariance of Initiation Mass and Predictability of Cell Size in Escherichia coli", Curr. Biol. **27**, 1278–1287 (2017).

[4] G.-W. Li, D. Burkhardt, C. Gross, and J. S. Weissman, "Quantifying absolute protein synthesis rates reveals principles underlying allocation of cellular resources", Cell **157**, 624–635 (2014).

[5] A. Schmidt, K. Kochanowski, S. Vedelaar, E. Ahrné, B. Volkmer, L. Callipo, K. Knoops, M. Bauer, R. Aebersold, and M. Heinemann, "The quantitative and condition-dependent Escherichia coli proteome", Nat. Biotechnol. **34**, 104–110 (2016).

[6] M. Mori, Z. Zhang, A. Banaei-Esfahani, J.-B. Lalanne, H. Okano, B. C. Collins, A. Schmidt, O. T. Schubert, D.-S. Lee, G.-W. Li, R. Aebersold, T. Hwa, and C. Ludwig, "From coarse to fine: the absolute Escherichia coli proteome under diverse growth conditions", Mol. Syst. Biol. **17**, e9536 (2021).

[7] P Pradhan, A. Taviti, and T. Beuria, "The bacterial division protein MinDE has an independent function in flagellation", Journal of Biological Chemistry **300**, Epub 2024 Feb 23, 107117 (2024).

[8] F Wu, B. G. C. van Schie, J. Keymer, and C. Dekker, "Symmetry and scale orient Min protein patterns in shaped bacterial sculptures", Nature Biotechnology **10**, 719–726 (2015).

[9] Z. Hu, E. P. Gogol, and J. Lutkenhaus, "Dynamic Assembly of MinD on Phospholipid Vesicles Regulated by ATP and MinE", Proceedings of the National Academy of Sciences **99**, 6761–6766 (2002).

[10] K. Suefuji, R. Valluzzi, and D. RayChaudhuri, "Dynamic Assembly of MinD into Filament Bundles Modulated by ATP, Phospholipids, and MinE", Proceedings of the National Academy of Sciences **99**, 16776–16781 (2002).

[11] V. Ivanov and K. Mizuuchi, "Multiple Modes of Interconverting Dynamic Pattern Formation by Bacterial Cell Division Proteins", Proceedings of the National Academy of Sciences **107**, 8071–8078 (2010).

[12] A. Miyagi, B. Ramm, P. Schwille, and S. Scheuring, "High-Speed Atomic Force Microscopy Reveals the Inner Workings of the MinDE Protein Oscillator", Nano Letters **18**, 288–296 (2018).

[13] T. Heermann, F. Steiert, B. Ramm, N. Hundt, and P. Schwille, "Mass-Sensitive Particle Tracking to Elucidate the Membrane-Associated MinDE Reaction Cycle", Nature Methods **18**, 1239–1246 (2021).

[14] J. Halatek, F. Brauns, and E. Frey, "Self-Organization Principles of Intracellular Pattern Formation", Philosophical Transactions of the Royal Society B: Biological Sciences **373**, 20170107 (2018).

[15] K. C. Huang, Y. Meir, and N. S. Wingreen, "Dynamic Structures in Escherichia Coli: Spontaneous Formation of MinE Rings and MinD Polar Zones", Proceedings of the National Academy of Sciences **100**, 12724–12728 (2003).

[16] J. Halatek and E. Frey, "Highly Canalized MinD Transfer and MinE Sequestration Explain the Origin of Robust MinCDE-Protein Dynamics", Cell Reports **1**, 741–752 (2012).

[17] J. Denk, S. Kretschmer, J. Halatek, C. Hartl, P. Schwille, and E. Frey, "MinE Conformational Switching Confers Robustness on Self-Organized Min Protein Patterns", Proceedings of the National Academy of Sciences **115**, 4553–4558 (2018).

[18] E. Frey and F. Brauns, "Self-Organization of Protein Patterns", in *Active Matter and Nonequilibrium Statistical Physics*, edited by J. Tailleur, G. Gompper, M. C. Marchetti, J. M. Yeomans, and C. Salomon, 1st ed. (Oxford University Press, Oxford, UK, Nov. 2022), pp. 347–445.

[19] J. Halatek and E. Frey, "Rethinking Pattern Formation in Reaction–Diffusion Systems", Nature Physics **14**, 507–514 (2018).

[20] T. Burkart, M. C. Wigbers, L. Würthner, and E. Frey, *Control of Protein-Based Pattern Formation via Guiding Cues*, Preprint (Biophysics, Feb. 2022).

[21] Z. Ren, H. Weyer, M. Sandler, L. Würthner, H. Fu, C. B. Tangtartharakul, D. Li, C. Sou, D. Villarreal, J. E. Kim, E. Frey, and S. Jun, *Supplementary Simulation Code*, Zenodo, Dec. 2024.

[22] *COMSOL Multiphysics*, COMSOL AB, Stockholm, Sweden, 2019.

[23] F. Brauns, J. Halatek, and E. Frey, "Phase-Space Geometry of Mass-Conserving Reaction-Diffusion Dynamics", Physical Review X **10**, 041036 (2020).

[24] M. C. Cross and P. C. Hohenberg, "Pattern Formation Outside of Equilibrium", Reviews of Modern Physics **65**, 851–1112 (1993).

[25] M. Cross and H. Greenside, *Pattern Formation and Dynamics in Nonequilibrium Systems* (Cambridge University Press, Cambridge, UK, 2009).

[26] D. M. Winterbottom, P. C. Matthews, and S. M. Cox, "Oscillatory Pattern Formation with a Conserved Quantity", Nonlinearity **18**, 1031–1056 (2005).

[27] T. Frohoff-Hülsmann and U. Thiele, "Nonreciprocal Cahn-Hilliard Model Emerges as a Universal Amplitude Equation", Physical Review Letters **131**, 107201 (2023).

[28] D. Greve and U. Thiele, *An Amplitude Equation for the Conserved-Hopf Bifurcation – Derivation, Analysis and Assessment*, 2024.

[29] D. Thalmeier, J. Halatek, and E. Frey, "Geometry-Induced Protein Pattern Formation", Proceedings of the National Academy of Sciences **113**, 548–553 (2016).

[30] G Meacci, J Ries, E Fischer-Friedrich, N Kahya, P Schwille, and K Kruse, "Mobility of Min-proteins in *Escherichia Coli* Measured by Fluorescence Correlation Spectroscopy", Physical Biology **3**, 255–263 (2006).
