# Supplementary material for: Robust and resource-optimal dynamic pattern formation of Min proteins in vivo
Source: Nat Phys. 2025 May 5;21(7):1160–9. doi: 10.1038/s41567-025-02878-w (PMC12263437; doi:10.1038/s41567-025-02878-w)

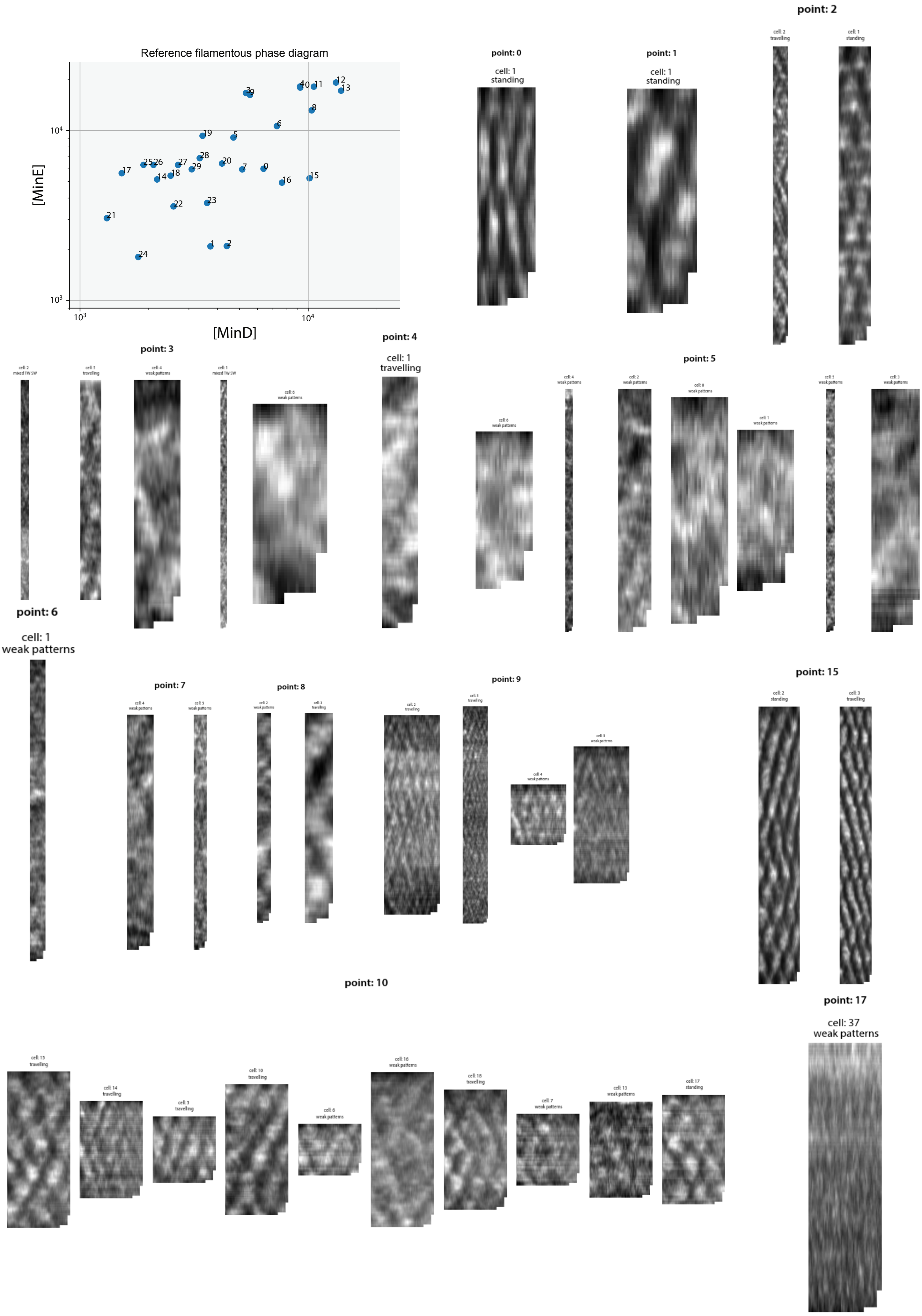

Reference filamentous phase diagram
[MinE]
[MinD]
point: 0
cell: 1 standing
point: 1
cell: 1 standing
point: 2
cell: 2 travelling
cell: 1 standing
point: 3
cell: 2 mixed TW SW
cell: 3 travelling
cell: 4 weak patterns
cell: 1 mixed TW SW
cell: 6 weak patterns
point: 4
cell: 1 travelling
point: 5
cell: 6 weak patterns
cell: 4 weak patterns
cell: 2 weak patterns
cell: 8 weak patterns
cell: 1 weak patterns
cell: 5 weak patterns
cell: 3 weak patterns
point: 6
cell: 1 weak patterns
point: 7
cell: 4 weak patterns
cell: 5 weak patterns
point: 8
cell: 2 weak patterns
cell: 3 travelling
point: 9
cell: 2 travelling
cell: 3 travelling
cell: 4 weak patterns
cell: 5 weak patterns
point: 15
cell: 2 standing
cell: 3 travelling
point: 10
cell: 15 travelling
cell: 14 travelling
cell: 5 travelling
cell: 10 travelling
cell: 6 weak patterns
cell: 16 weak patterns
cell: 18 travelling
cell: 7 weak patterns
cell: 13 weak patterns
cell: 17 standing
point: 17
cell: 37 weak patterns

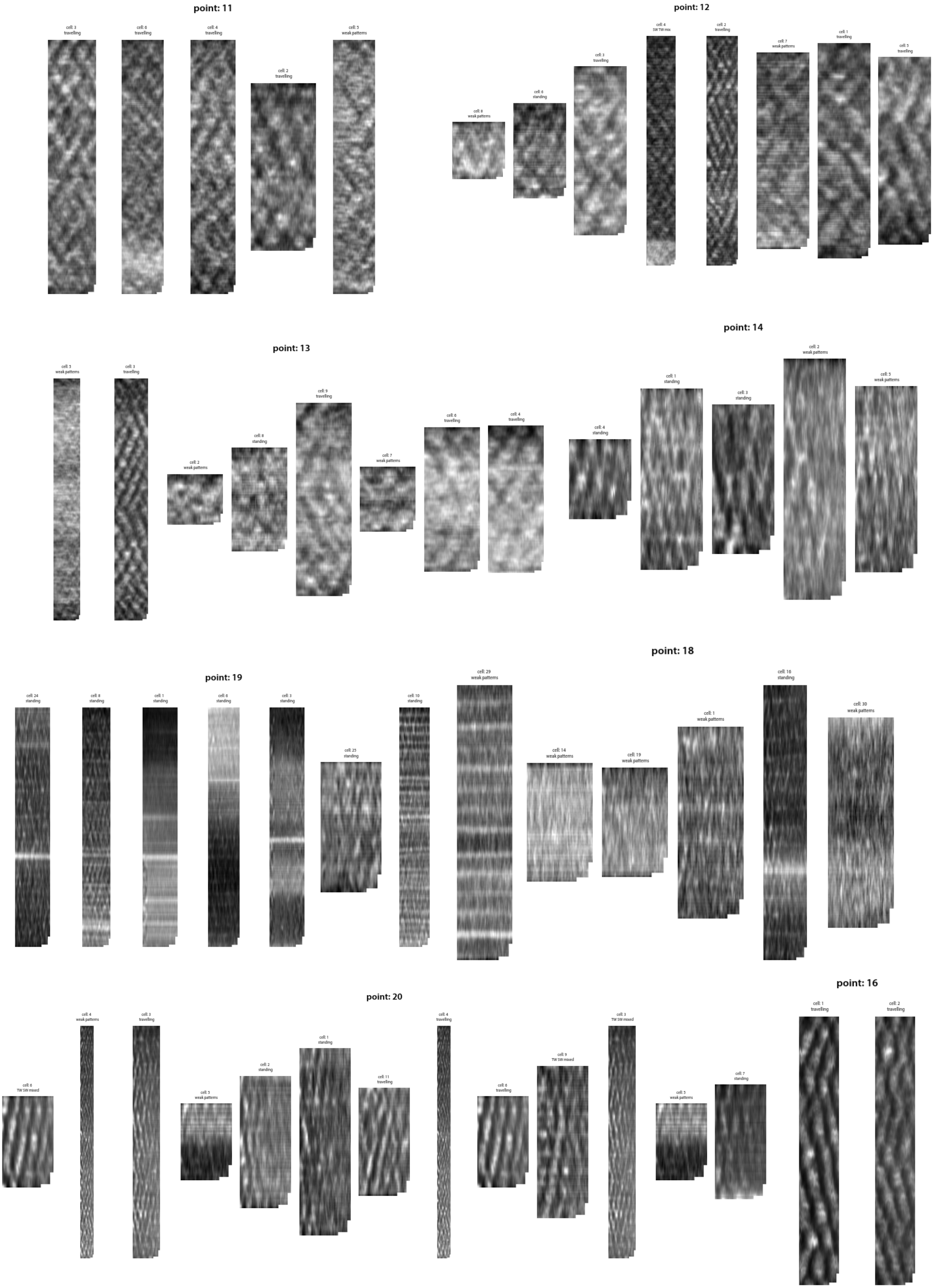

point: 11
cell: 3 travelling
cell: 6 travelling
cell: 4 travelling
cell: 2 travelling
cell: 5 weak patterns
point: 12
cell: 8 weak patterns
cell: 6 standing
cell: 3 travelling
cell: 4 SW TW mix
cell: 2 travelling
cell: 7 weak patterns
cell: 1 travelling
cell: 5 travelling
point: 13
cell: 5 weak patterns
cell: 3 travelling
cell: 2 weak patterns
cell: 8 standing
cell: 9 travelling
cell: 7 weak patterns
cell: 6 travelling
cell: 4 travelling
point: 14
cell: 4 standing
cell: 1 standing
cell: 3 standing
cell: 2 weak patterns
cell: 5 weak patterns
point: 19
cell: 24 standing
cell: 8 standing
cell: 1 standing
cell: 6 standing
cell: 3 standing
cell: 25 standing
cell: 10 standing
point: 18
cell: 29 weak patterns
cell: 14 weak patterns
cell: 19 weak patterns
cell: 1 weak patterns
cell: 16 standing
cell: 30 weak patterns
point: 20
cell: 6 TW SW mixed
cell: 4 weak patterns
cell: 3 travelling
cell: 5 weak patterns
cell: 2 standing
cell: 1 standing
cell: 11 travelling
cell: 4 travelling
cell: 6 travelling
cell: 9 TW SW mixed
cell: 3 TW SW mixed
cell: 5 weak patterns
cell: 7 standing
point: 16
cell: 1 travelling
cell: 2 travelling

point: 21

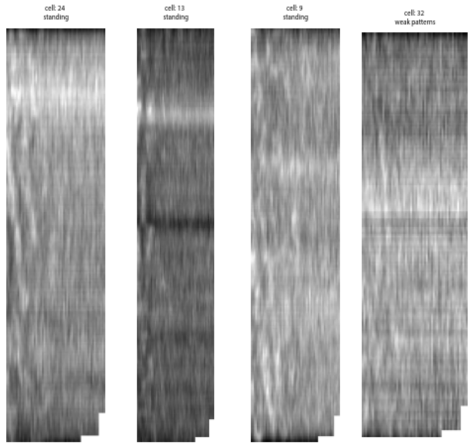

point: 22

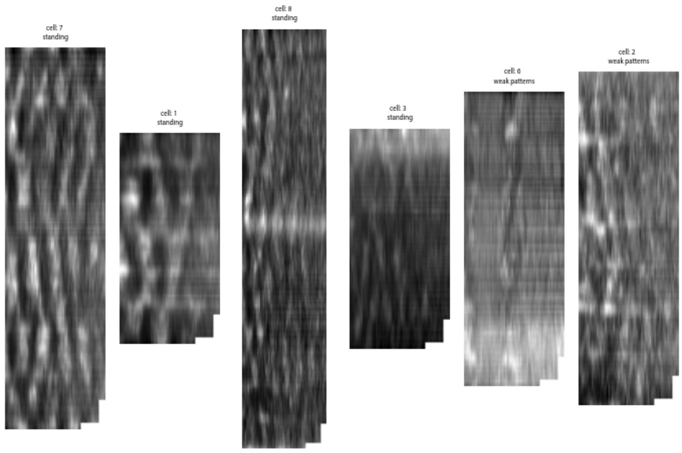

point: 23

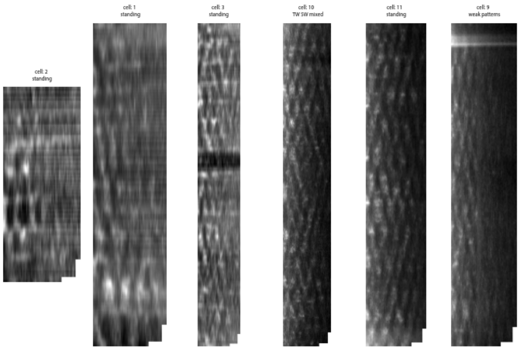

point: 25

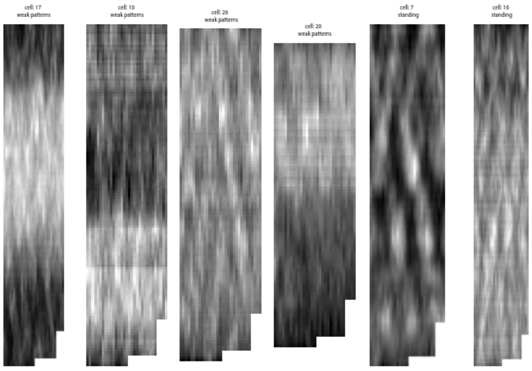

point: 26

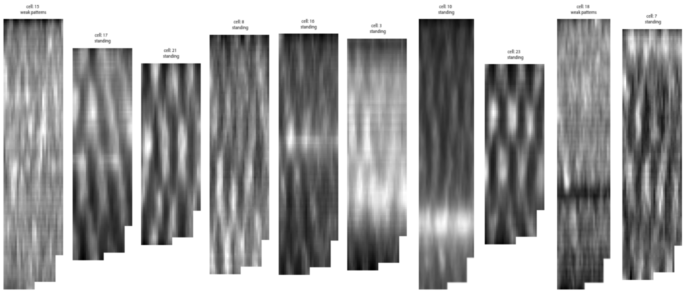

point: 27

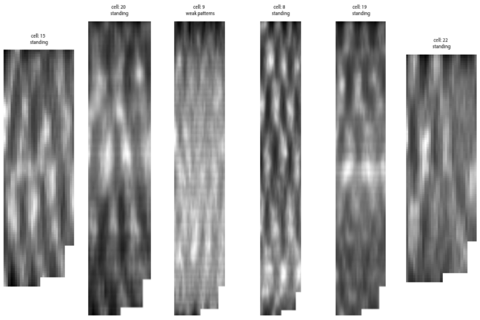

point: 28

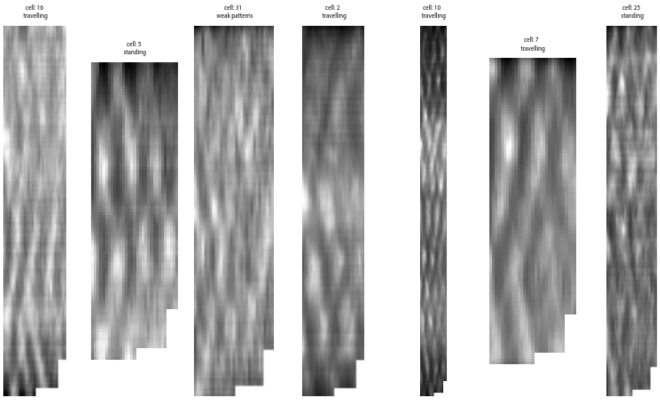

point: 29

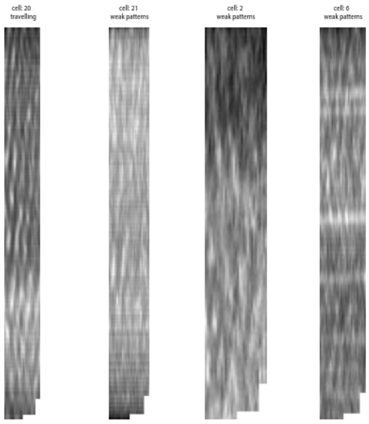

Supplement: Supplementary file 11 — Statistical source data for Figs. 2e and 3c and cell kymographs with annotations for a comparison with the theoretical results. [file 41567_2025_2878_MOESM11_ESM.zip › Source Data/Fig3/Fig3c_filamentous-kymos/summary_filamentous-reference-kymos.pdf]
