# Supplementary figures and images for: Robust and resource-optimal dynamic pattern formation of Min proteins in vivo
Source: Nat Phys. 2025 May 5;21(7):1160–9. doi: 10.1038/s41567-025-02878-w (PMC12263437; doi:10.1038/s41567-025-02878-w)

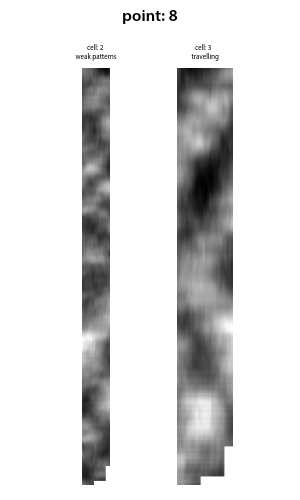

Supplement: Supplementary file 11 — Statistical source data for Figs. 2e and 3c and cell kymographs with annotations for a comparison with the theoretical results. [file 41567_2025_2878_MOESM11_ESM.zip › Source Data/Fig3/Fig3c_filamentous-kymos/8.png]

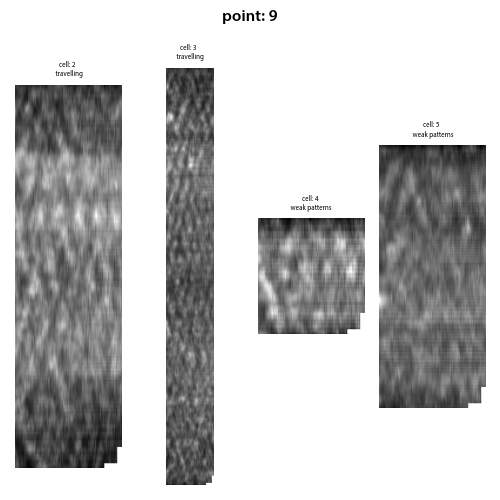

Supplement: Supplementary file 11 — Statistical source data for Figs. 2e and 3c and cell kymographs with annotations for a comparison with the theoretical results. [file 41567_2025_2878_MOESM11_ESM.zip › Source Data/Fig3/Fig3c_filamentous-kymos/9.png]

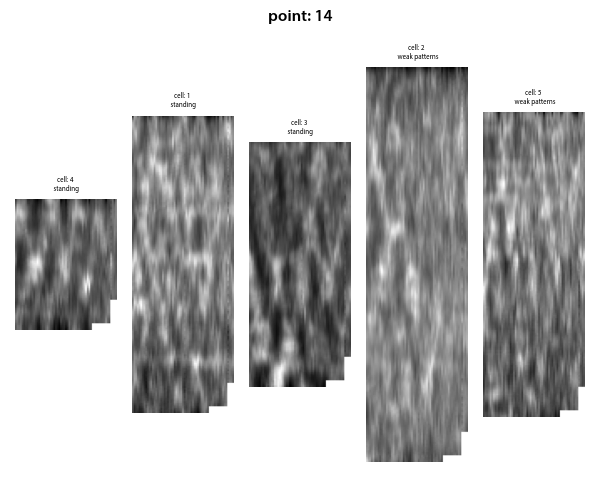

Supplement: Supplementary file 11 — Statistical source data for Figs. 2e and 3c and cell kymographs with annotations for a comparison with the theoretical results. [file 41567_2025_2878_MOESM11_ESM.zip › Source Data/Fig3/Fig3c_filamentous-kymos/14.png]

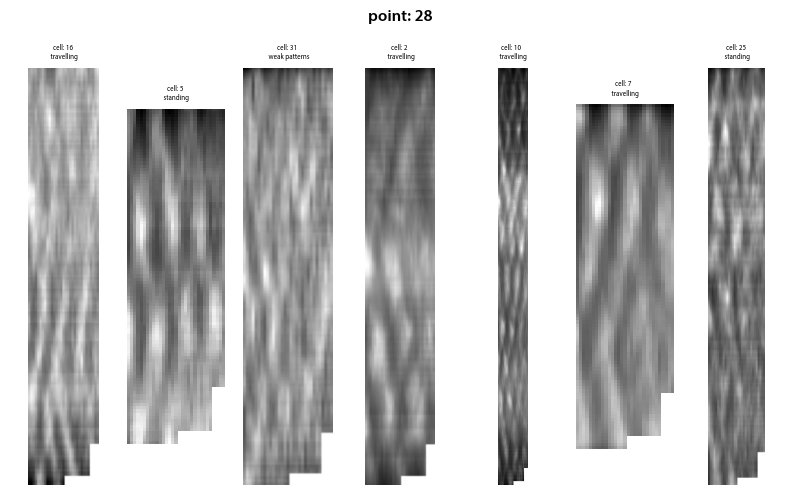

Supplement: Supplementary file 11 — Statistical source data for Figs. 2e and 3c and cell kymographs with annotations for a comparison with the theoretical results. [file 41567_2025_2878_MOESM11_ESM.zip › Source Data/Fig3/Fig3c_filamentous-kymos/28.png]

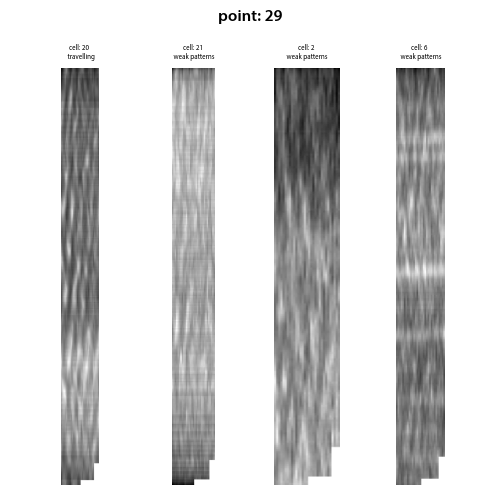

Supplement: Supplementary file 11 — Statistical source data for Figs. 2e and 3c and cell kymographs with annotations for a comparison with the theoretical results. [file 41567_2025_2878_MOESM11_ESM.zip › Source Data/Fig3/Fig3c_filamentous-kymos/29.png]

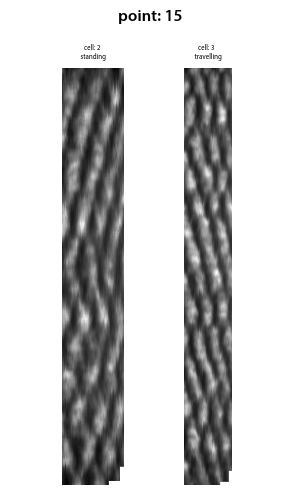

Supplement: Supplementary file 11 — Statistical source data for Figs. 2e and 3c and cell kymographs with annotations for a comparison with the theoretical results. [file 41567_2025_2878_MOESM11_ESM.zip › Source Data/Fig3/Fig3c_filamentous-kymos/15.png]

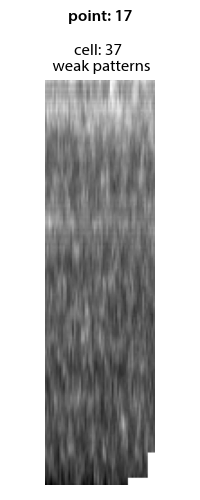

Supplement: Supplementary file 11 — Statistical source data for Figs. 2e and 3c and cell kymographs with annotations for a comparison with the theoretical results. [file 41567_2025_2878_MOESM11_ESM.zip › Source Data/Fig3/Fig3c_filamentous-kymos/17.png]

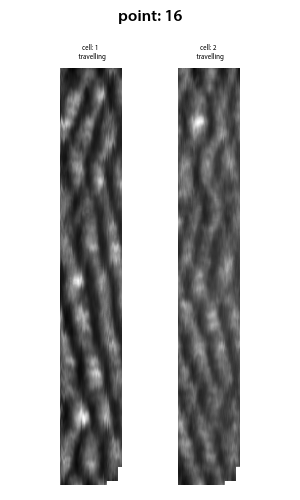

Supplement: Supplementary file 11 — Statistical source data for Figs. 2e and 3c and cell kymographs with annotations for a comparison with the theoretical results. [file 41567_2025_2878_MOESM11_ESM.zip › Source Data/Fig3/Fig3c_filamentous-kymos/16.png]

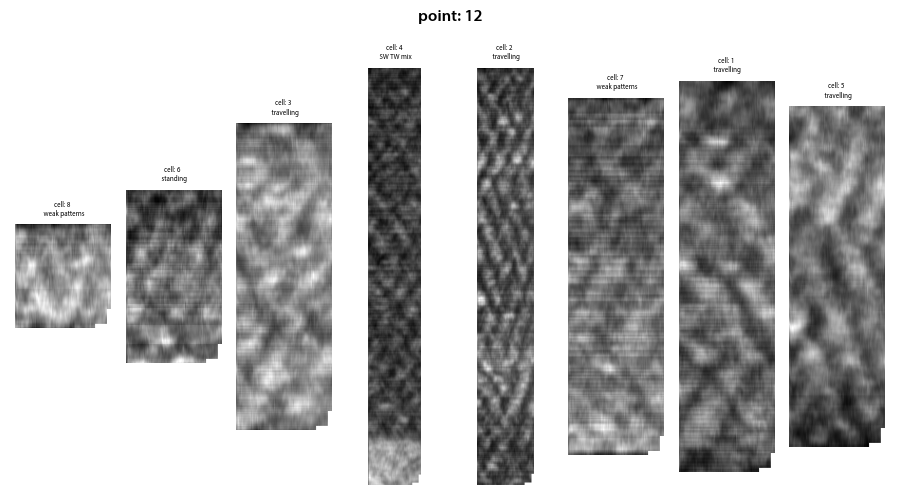

Supplement: Supplementary file 11 — Statistical source data for Figs. 2e and 3c and cell kymographs with annotations for a comparison with the theoretical results. [file 41567_2025_2878_MOESM11_ESM.zip › Source Data/Fig3/Fig3c_filamentous-kymos/12.png]

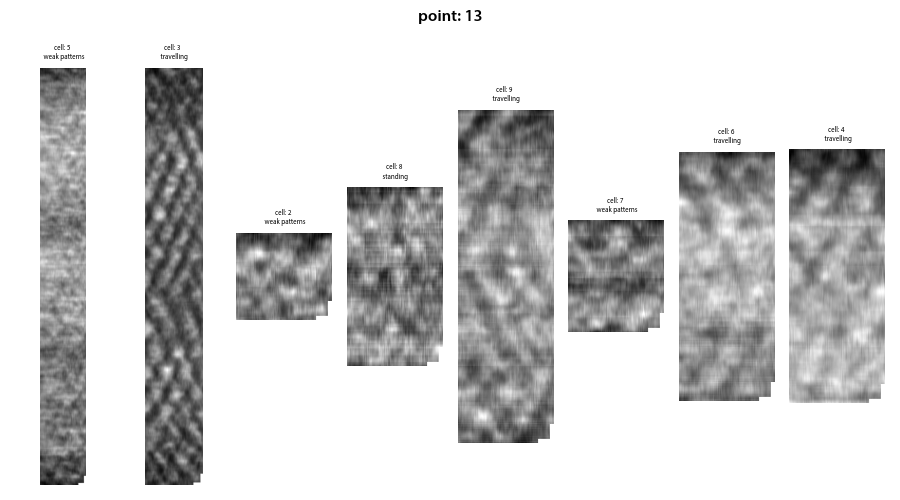

Supplement: Supplementary file 11 — Statistical source data for Figs. 2e and 3c and cell kymographs with annotations for a comparison with the theoretical results. [file 41567_2025_2878_MOESM11_ESM.zip › Source Data/Fig3/Fig3c_filamentous-kymos/13.png]

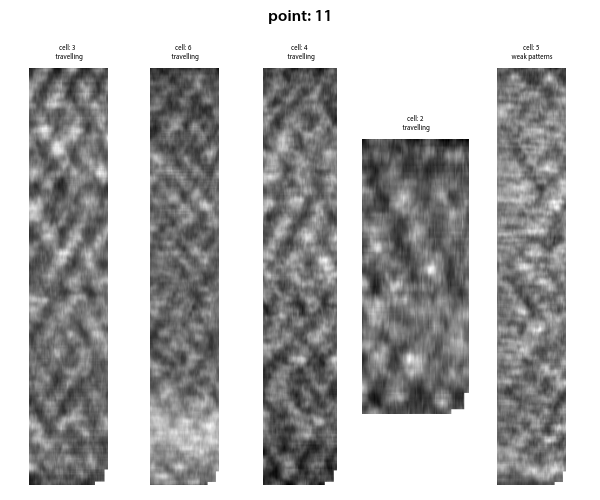

Supplement: Supplementary file 11 — Statistical source data for Figs. 2e and 3c and cell kymographs with annotations for a comparison with the theoretical results. [file 41567_2025_2878_MOESM11_ESM.zip › Source Data/Fig3/Fig3c_filamentous-kymos/11.png]

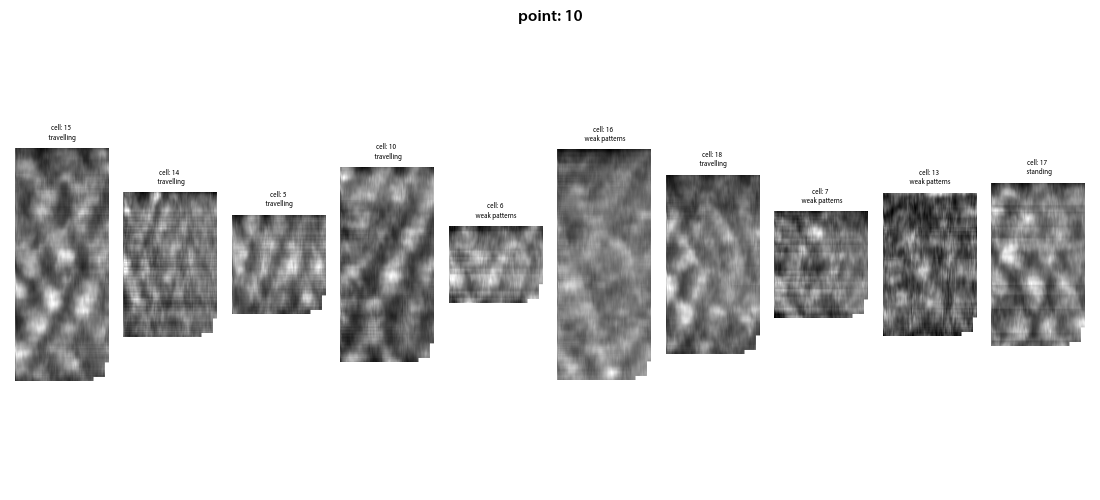

Supplement: Supplementary file 11 — Statistical source data for Figs. 2e and 3c and cell kymographs with annotations for a comparison with the theoretical results. [file 41567_2025_2878_MOESM11_ESM.zip › Source Data/Fig3/Fig3c_filamentous-kymos/10.png]

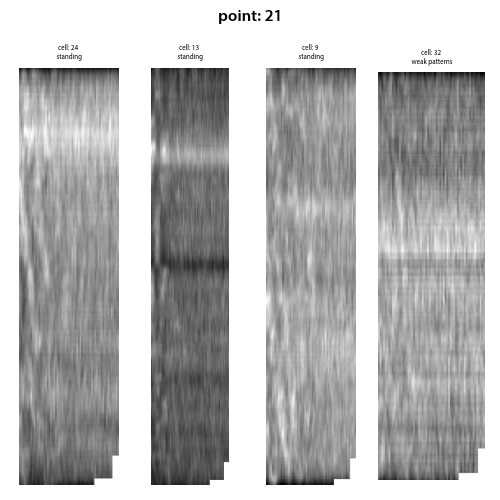

Supplement: Supplementary file 11 — Statistical source data for Figs. 2e and 3c and cell kymographs with annotations for a comparison with the theoretical results. [file 41567_2025_2878_MOESM11_ESM.zip › Source Data/Fig3/Fig3c_filamentous-kymos/21.png]

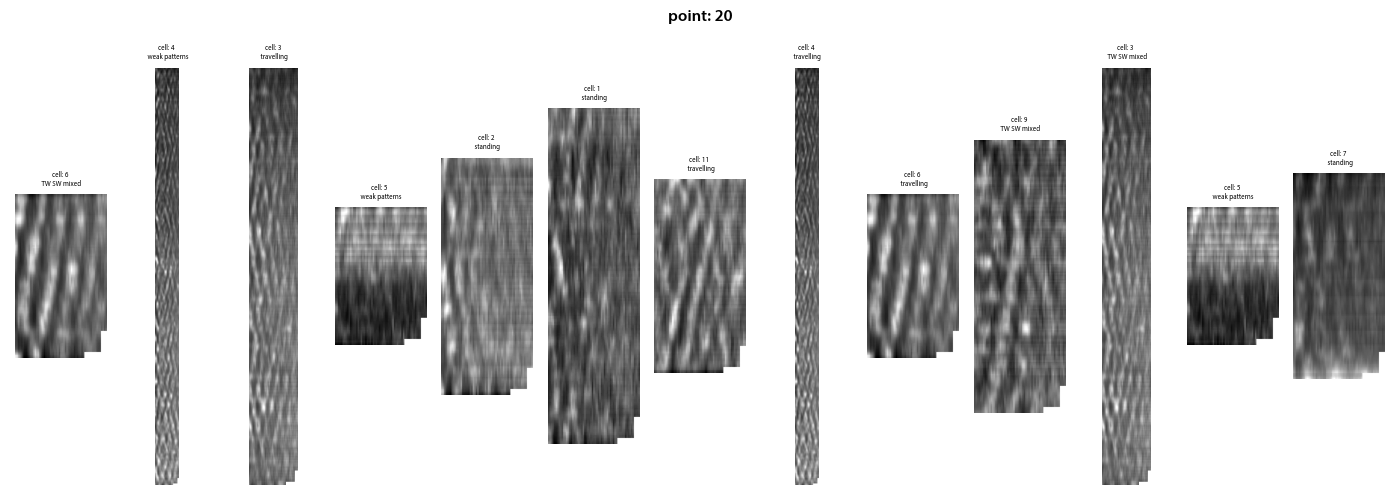

Supplement: Supplementary file 11 — Statistical source data for Figs. 2e and 3c and cell kymographs with annotations for a comparison with the theoretical results. [file 41567_2025_2878_MOESM11_ESM.zip › Source Data/Fig3/Fig3c_filamentous-kymos/20.png]

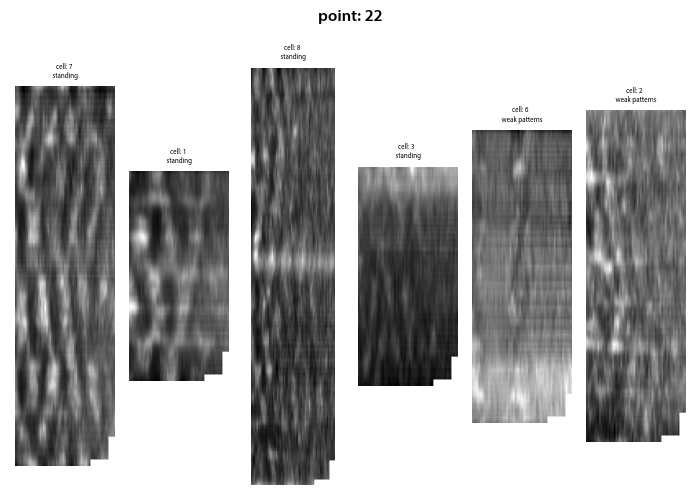

Supplement: Supplementary file 11 — Statistical source data for Figs. 2e and 3c and cell kymographs with annotations for a comparison with the theoretical results. [file 41567_2025_2878_MOESM11_ESM.zip › Source Data/Fig3/Fig3c_filamentous-kymos/22.png]

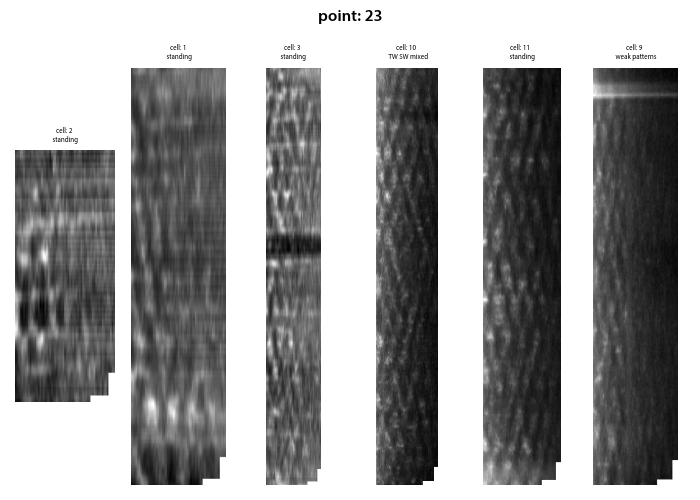

Supplement: Supplementary file 11 — Statistical source data for Figs. 2e and 3c and cell kymographs with annotations for a comparison with the theoretical results. [file 41567_2025_2878_MOESM11_ESM.zip › Source Data/Fig3/Fig3c_filamentous-kymos/23.png]

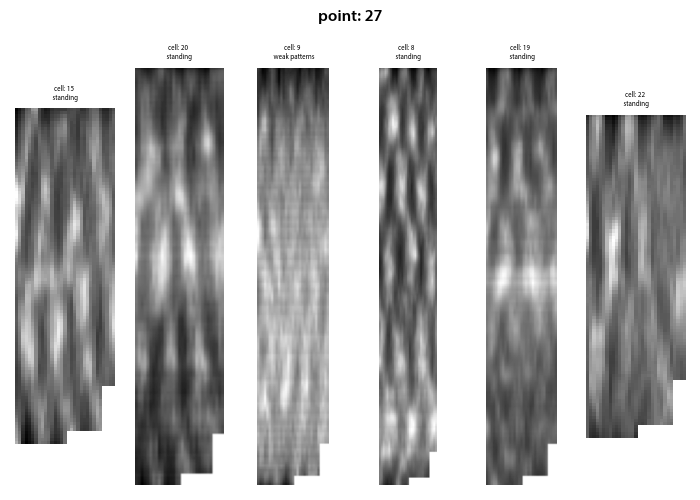

Supplement: Supplementary file 11 — Statistical source data for Figs. 2e and 3c and cell kymographs with annotations for a comparison with the theoretical results. [file 41567_2025_2878_MOESM11_ESM.zip › Source Data/Fig3/Fig3c_filamentous-kymos/27.png]

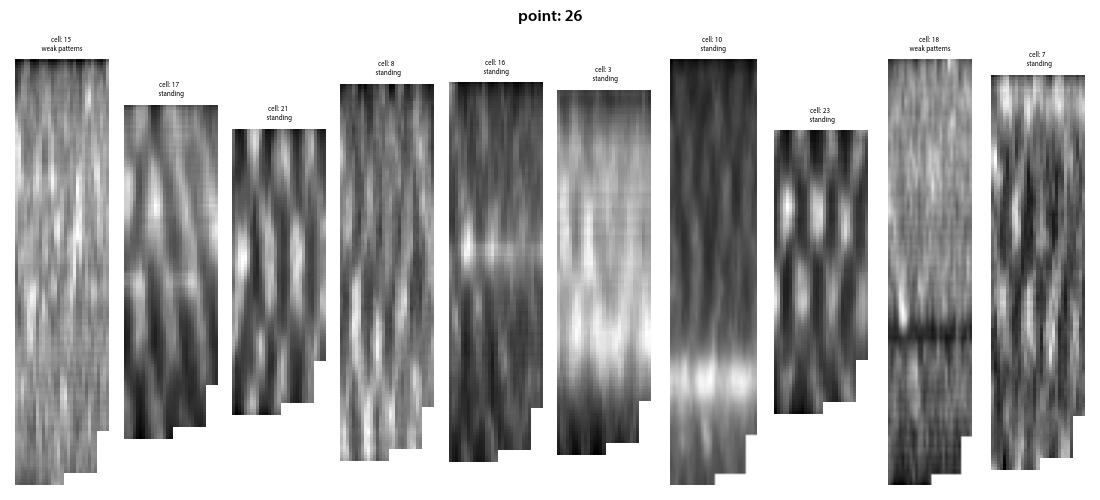

Supplement: Supplementary file 11 — Statistical source data for Figs. 2e and 3c and cell kymographs with annotations for a comparison with the theoretical results. [file 41567_2025_2878_MOESM11_ESM.zip › Source Data/Fig3/Fig3c_filamentous-kymos/26.png]

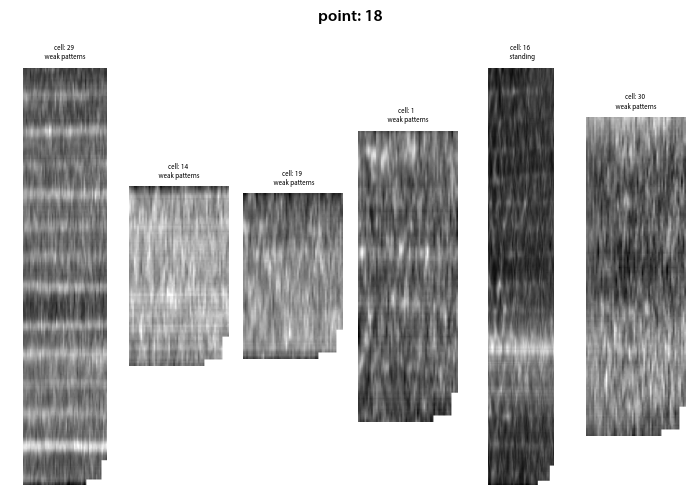

Supplement: Supplementary file 11 — Statistical source data for Figs. 2e and 3c and cell kymographs with annotations for a comparison with the theoretical results. [file 41567_2025_2878_MOESM11_ESM.zip › Source Data/Fig3/Fig3c_filamentous-kymos/18.png]

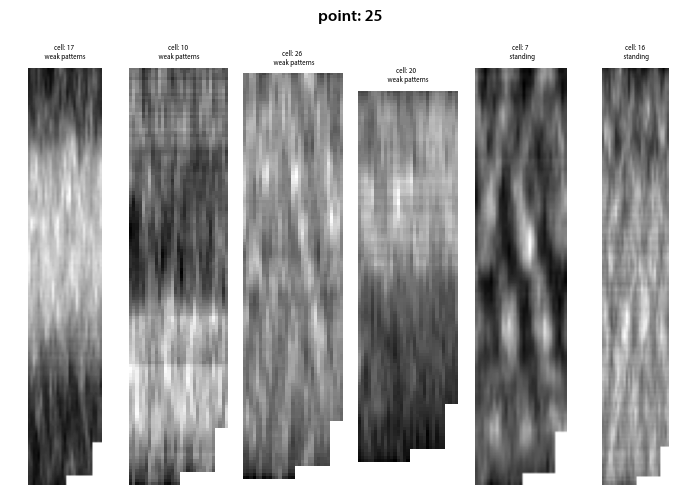

Supplement: Supplementary file 11 — Statistical source data for Figs. 2e and 3c and cell kymographs with annotations for a comparison with the theoretical results. [file 41567_2025_2878_MOESM11_ESM.zip › Source Data/Fig3/Fig3c_filamentous-kymos/25.png]

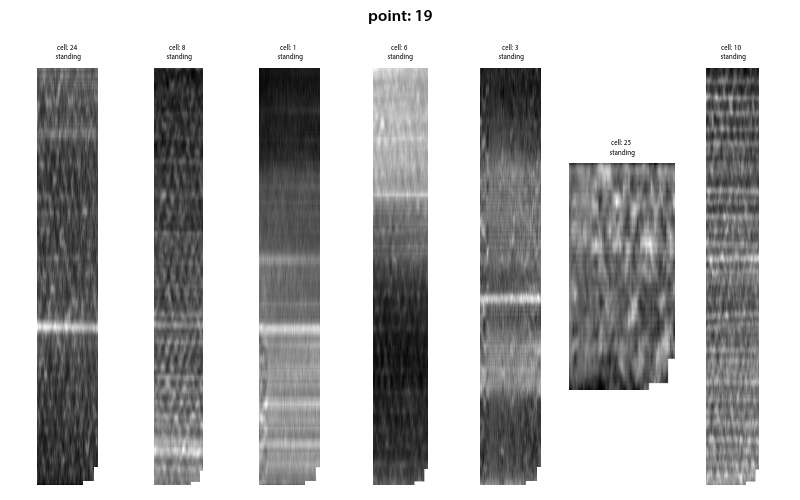

Supplement: Supplementary file 11 — Statistical source data for Figs. 2e and 3c and cell kymographs with annotations for a comparison with the theoretical results. [file 41567_2025_2878_MOESM11_ESM.zip › Source Data/Fig3/Fig3c_filamentous-kymos/19.png]

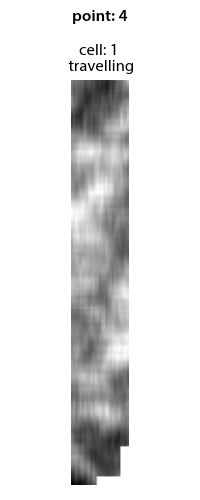

Supplement: Supplementary file 11 — Statistical source data for Figs. 2e and 3c and cell kymographs with annotations for a comparison with the theoretical results. [file 41567_2025_2878_MOESM11_ESM.zip › Source Data/Fig3/Fig3c_filamentous-kymos/4.png]

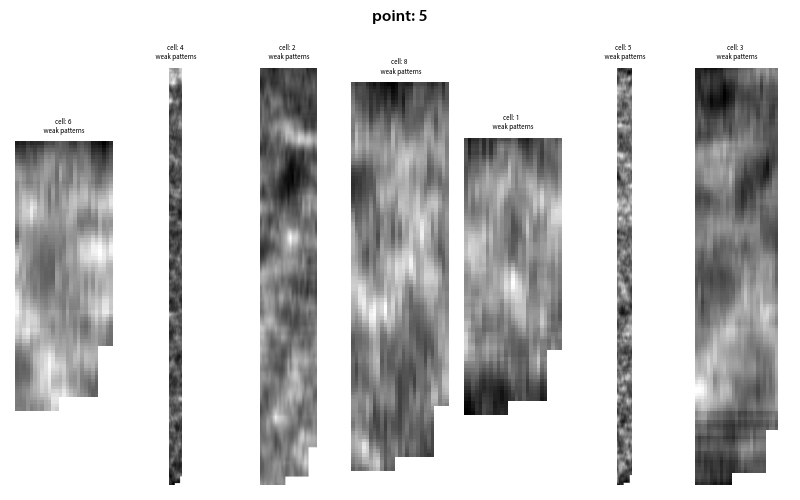

Supplement: Supplementary file 11 — Statistical source data for Figs. 2e and 3c and cell kymographs with annotations for a comparison with the theoretical results. [file 41567_2025_2878_MOESM11_ESM.zip › Source Data/Fig3/Fig3c_filamentous-kymos/5.png]

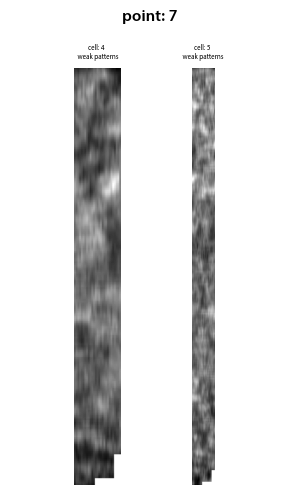

Supplement: Supplementary file 11 — Statistical source data for Figs. 2e and 3c and cell kymographs with annotations for a comparison with the theoretical results. [file 41567_2025_2878_MOESM11_ESM.zip › Source Data/Fig3/Fig3c_filamentous-kymos/7.png]

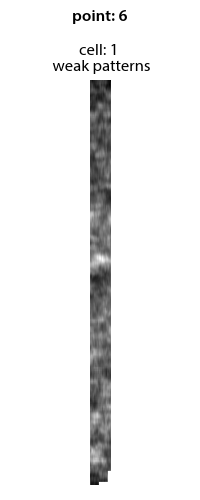

Supplement: Supplementary file 11 — Statistical source data for Figs. 2e and 3c and cell kymographs with annotations for a comparison with the theoretical results. [file 41567_2025_2878_MOESM11_ESM.zip › Source Data/Fig3/Fig3c_filamentous-kymos/6.png]

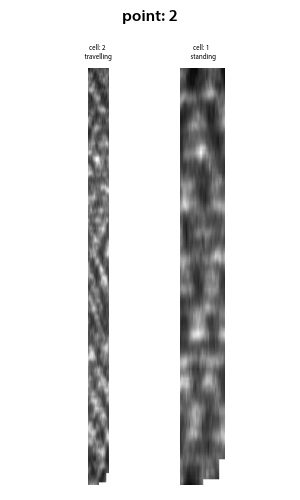

Supplement: Supplementary file 11 — Statistical source data for Figs. 2e and 3c and cell kymographs with annotations for a comparison with the theoretical results. [file 41567_2025_2878_MOESM11_ESM.zip › Source Data/Fig3/Fig3c_filamentous-kymos/2.png]

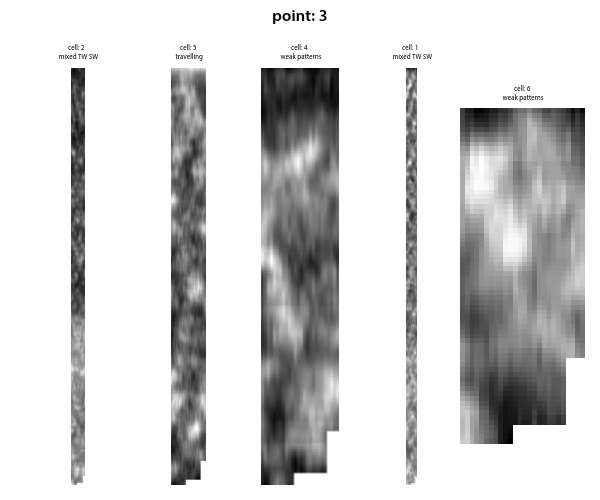

Supplement: Supplementary file 11 — Statistical source data for Figs. 2e and 3c and cell kymographs with annotations for a comparison with the theoretical results. [file 41567_2025_2878_MOESM11_ESM.zip › Source Data/Fig3/Fig3c_filamentous-kymos/3.png]

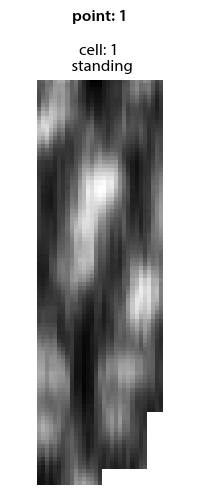

Supplement: Supplementary file 11 — Statistical source data for Figs. 2e and 3c and cell kymographs with annotations for a comparison with the theoretical results. [file 41567_2025_2878_MOESM11_ESM.zip › Source Data/Fig3/Fig3c_filamentous-kymos/1.png]

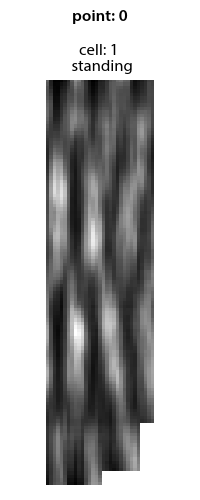

Supplement: Supplementary file 11 — Statistical source data for Figs. 2e and 3c and cell kymographs with annotations for a comparison with the theoretical results. [file 41567_2025_2878_MOESM11_ESM.zip › Source Data/Fig3/Fig3c_filamentous-kymos/0.png]
